# Epidemiology of Dengue Virus in Iquitos, Peru 1999 to 2005: Interepidemic and Epidemic Patterns of Transmission

**DOI:** 10.1371/journal.pntd.0000670

**Published:** 2010-05-04

**Authors:** Amy C. Morrison, Sharon L. Minnick, Claudio Rocha, Brett M. Forshey, Steven T. Stoddard, Arthur Getis, Dana A. Focks, Kevin L. Russell, James G. Olson, Patrick J. Blair, Douglas M. Watts, Moises Sihuincha, Thomas W. Scott, Tadeusz J. Kochel

**Affiliations:** 1 Department of Entomology, University of California Davis, Davis, California, United States of America; 2 Naval Medical Research Center Detachment, Washington, D. C., United States of America; 3 Department of Geography, San Diego State University, San Diego, California, United States of America; 4 Infectious Disease Analysis, Gainesville, Florida, United States of America; 5 Loreto Regional Reference Laboratory, Loreto Regional Health Department, Iquitos, Peru; University of Texas Medical Branch, United States of America

## Abstract

**Background:**

Comprehensive, longitudinal field studies that monitor both disease and vector populations for dengue viruses are urgently needed as a pre-requisite for developing locally adaptable prevention programs or to appropriately test and license new vaccines.

**Methodology and Principal Findings:**

We report the results from such a study spanning 5 years in the Amazonian city of Iquitos, Peru where DENV infection was monitored serologically among ∼2,400 members of a neighborhood-based cohort and through school-based absenteeism surveillance for active febrile illness among a subset of this cohort. At baseline, 80% of the study population had DENV antibodies, seroprevalence increased with age, and significant geographic variation was observed, with neighborhood-specific age-adjusted rates ranging from 67.1 to 89.9%. During the first 15 months, when DENV-1 and DENV-2 were co-circulating, population-based incidence rates ranged from 2–3 infections/100 person-years (p-years). The introduction of DENV-3 during the last half of 2001 was characterized by 3 distinct periods: amplification over at least 5–6 months, replacement of previously circulating serotypes, and epidemic transmission when incidence peaked at 89 infections/100 p-years.

**Conclusions/Significance:**

Neighborhood-specific baseline seroprevalence rates were not predictive of geographic incidence patterns prior to the DENV-3 introduction, but were closely mirrored during the invasion of this serotype. Transmission varied geographically, with peak incidence occurring at different times among the 8 geographic zones in ∼16 km^2^ of the city. The lag from novel serotype introduction to epidemic transmission and knowledge of spatially explicit areas of elevated risk should be considered for more effective application of limited resources for dengue prevention.

## Author Summary

To develop prevention (including vaccines) and control programs for dengue fever, a significant mosquito-borne disease in the tropics, there is an urgent need for comprehensive long term field epidemiological studies. We report results from a study that monitored ∼2,400 school children and some adult family members for dengue infection at 6 month intervals from 1999 to 2005, in the Amazonian city of Iquitos, Peru. At enrollment, ∼80% of the participants had a previous infection with DENV serotypes 1 and 2 or both. During the first 15 months, about 3 new infections for every 100 participants were observed among the study participants. In 2001, DENV-3, a serotype not previously observed in the region, invaded Iquitos in a process characterized by 3 distinct periods: amplification over at least a 5–6 month period, replacement of previously circulating serotypes, and epidemic transmission when incidence peaked. Incidence patterns of new infections were geographically distinct from baseline prevalence rates prior to arrival of DENV-3, but closely mirrored them during the invasion. DENV transmission varied geographically corresponding to elevated mosquito densities. The invasion of a novel serotype is often characterized by 5–6 months of silent transmission before traditional surveillance programs detect the virus. This article sets the stage for subsequent publications on dengue epidemiology.

## Introduction

Dengue viruses (DENV) are major re-emerging pathogens that have increased geographically from only 9 countries 60 years ago to more than 100 today. An estimated 2.5–3.0 billion people worldwide are at risk, with 50–100 million cases of dengue fever (DF) and 250,000–500,000 of the more severe dengue hemorrhagic fever (DHF) and dengue shock syndrome (DSS) each year. Incidence of severe disease (DHF/DSS) has been increasing consistently since the 1950's [1], [2], [3], [4], [5].

DENV exists as four closely-related, antigenically distinct single-stranded RNA viruses (DENV-1, DENV-2, DENV-3 and DENV-4) in the genus *Flavivirus*, family *Flaviridae*. Immunity induced by infection with one serotype is protective and affords transient cross-protection against the other serotypes; hence sequential infections with different serotypes are possible. The etiology of serious illness is not completely understood, but secondary infection and/or variation in virus virulence have often been implicated [1], [4], [5], [6], [7], [8].

Without a vaccine, dengue prevention relies on virological surveillance and vector control. Mounting evidence indicates that accounting for variation in the ecology and epidemiology of dengue will be important for development of more effective, locally adapted control programs [9], [10]. Such programs, along with future phase III vaccine trials, will require an improved understanding of region-specific transmission dynamics. This information is best obtained from comprehensive, longitudinal field studies designed to provide a detailed understanding of fundamental processes in virus transmission, epidemiology and disease control [9].

In Latin America, successful control efforts that ended in the 1960's were followed by reinvasion of the mosquito vector *Aedes aegypti* and the emergence of dengue as a leading public health problem throughout the continent. Wide-spread urbanization contributed to spread of the vector, creating conditions that enhanced DENV transmission. This is exemplified by numerous recent and dramatic regional outbreaks. Here we report results from longitudinal studies in Iquitos, Peru, an Amazonian city with a history of dengue virus transmission that has been well documented by the US Naval Medical Research Center Detachment (NMRCD) since the early 1990's, when DENV was presumably reintroduced into Peru [11]. Iquitos experienced epidemics of febrile disease caused by sequential invasions of DENV: DENV-1 in 1990—1991, an American strain of DENV-2 in 1995 [7], [12], DENV-3 in 2001 [13], an Asian strain of DENV-2 in 2002, and DENV-4 in 2008 [14].

The long-term goal of our research in Iquitos was to acquire a detailed understanding of dynamics in DENV transmission and their relationship to entomological parameters that will inform vector control programs and improve disease prevention. Our approach was to monitor virus transmission and *Ae. aegypti* population densities simultaneously in the homes and neighborhoods of a longitudinal cohort representing 20% of the most populated areas of the city. *Ae. aegypti* abundance and production patterns were previously described [15], [16], [17], [18]. Herein, we examine spatial and temporal patterns of transmission dynamics for 3 DENV serotypes before and during the invasion of a locally novel virus. Our results are based on a prospective cohort study conducted between 1999–2005, during which time there was active transmission of DENV-1 and DENV-2 and invasion of DENV-3, which caused a significant epidemic of febrile disease.

## Materials and Methods

### Human Use Statement

The study protocol was approved by the University of California, Davis (Protocol 2220210788-4(994054), Instituto Nacional de Salud, and Naval Medical Research Center (Protocol #NMRCD.2001.0008 [DoD 31574]) Institutional Review Boards in compliance with all Federal regulations governing the protection of human subjects.

### Study Area

Our study was conducted in Iquitos, an urban community located in the Amazon Basin of northeastern Peru (73.2°W, 3.7°S, 120 m above sea level) in the Department of Loreto. The Amazon, Nanay, and Itaya Rivers surround it on 3 sides. The population in the city has grown since its last published census of 350,000 people [19]. The more common industries are small business, fishing, oil, lumber, tourism and agriculture. The climate is tropical, with an average daily temperature of 25.8°C (average minimum 21.9°C and maximum 32.4°C) and an average annual precipitation of 3.4 meters (range 2.7—4.4 meters) during our study. Precipitation occurs throughout the year, on about half the days (51.6%). Iquitos is described in detail in earlier reports [7], [12], [15], [16], [17], [18].

Iquitos is comprised of 4 districts: San Juan, Maynas, and Punchana running from South to North and Belen on the East (See Figure 1 in [17]). We restricted our study to an area of ≈16 km^2^ in the districts of Maynas, Punchana and small portions of Belen and San Juan, which we divided into 8 geographic zones (described in detail in [17]) based on known neighborhoods served by distinct health centers. In brief, the 3 most northern zones – Punchana (PU), Maynas (MY), and San Antonio (SA) – and the 5 remaining southern zones – Putumayo (PT), Iquitos (IQ), Morona Cocha (MC), Bagazan (BG) and Tupac Amaru (TA) – belong to the districts of Punchana and Maynas, respectively; each has its own local government and services. The zones of Tupac Amaru (TA), Bagazan (BG), and Putumayo (PT) each have areas where houses are flooded seasonally.

**Figure 1:**
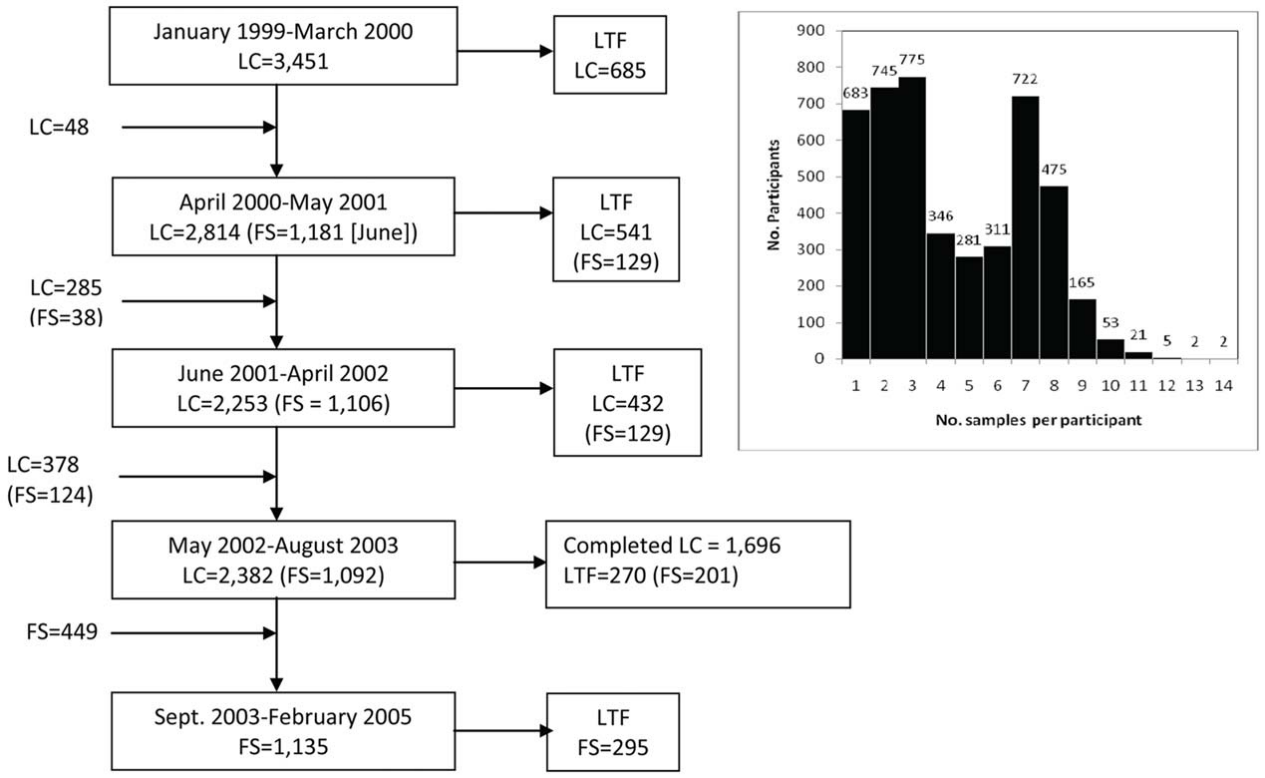
Participant enrollment and lost to follow up (LTF) for longitudinal cohort (LC) and subset school-based febrile surveillance (FS) studies carried out between January 1999 and February 2005. All participants enrolled in LC provided blood samples taken at ∼6-month intervals that were tested by PRNT for DENV NtAbs to identify seroconversions. Entomological surveys were carried out through August 2003. Participation in FS included the LC activities plus absence monitoring in school initiated in June 2000. After August 2003, only FS participants continued to be monitored serologically. Absence monitoring in schools continued through December 2004. Participants recruited after August 2003 provided serological samples for PRNT testing and all students finishing the study provided a final serological sample between December 2004–February 2005. **Inset:** The distribution of the number of blood samples taken at intervals from individual participants.

### Study Design

We monitored a cohort of ∼2,400 study participants longitudinally from January 1999 through August 2003 at ∼6-month intervals for serological evidence of DENV infection. In addition, serological monitoring continued until February 2005 in a subset of school age cohort participants who were also monitored for active dengue disease based on attendance at school (see sub-section **febrile surveillance** below). To obtain a geographically and temporally stratified sample of study participants, each of the 8 study zones was sub-divided into approximately 5 equal areas. Three blocks from each area were randomly selected for a total of 15 sample blocks in each zone. Recruitment focused on school-aged children (between 5 and 20 years). To obtain a more stratified cross-section of the population, participation was also offered to other members of the household after a school-aged participant was enrolled. If the residents agreed to participate, the consent and assent forms were signed before samples were obtained. Written informed consent was obtained from participants older than 17 years, and from parents of participants younger than 18. In addition, assent was obtained from participants 8–17 years of age. If participants were unable to read and sign the consent form, oral consent was obtained and documented in the presence of a witness. Fifty school-aged participants and 10 family members older than 20 were recruited from each zone each month, repeating the process each month until a base cohort of 2,400 individuals was obtained (Table S1; see Figure 1 in [20]).

After the base cohort was established, follow-up visits for individual participants were carried out at approximately 6-month intervals. Participants were considered lost to follow up after a full year had passed since their previous blood draw, despite repeated attempts to locate the participant, or if there was a verifiable reason for dropping them from the study (direct request from the participant, movement from the study area, or death). New participants were enrolled to replace those lost to follow up, with preference toward people from the same geographic zones as those lost, in order to maintain an active cohort of ∼2,400 individuals. In some cases participants would re-enroll in the study after returning from an extended trip or regaining interest in the study.

In June 2000, a subset of 1,100 cohort members (ages 5—20) who were attending morning sessions at one of the 29 participating public schools in the city were recruited for surveillance of febrile disease [20]. In addition to participating in the longitudinal components of the study (6-month blood samples and entomological surveys) this sub-population was monitored for symptomatic DENV cases. Phlebotomists visited schools daily, checked attendance, and visited the homes of participating absentees. If the absence was caused by febrile illness, acute and convalescent blood samples were obtained and daily medical exams carried out for each participant. During school vacation, children were visited weekly at their homes. New participants were enrolled to replace those who had dropped out of school, graduated, or changed schools. After entomological surveillance ended in August 2003, the school surveillance study continued for participants who remained in monitored schools. At the beginning of the 2004 school year, additional participants were recruited from previously enrolled families (siblings or other relatives) or from households participating in a community-based cohort study [20]. Monitoring of absences ended in December 2004, and final blood samples were obtained from children up to March 2005. Clinical aspects of this study will be reported in subsequent publications.

#### Blood sample collection

Blood samples were obtained by venipuncture or fingerstick using standard aseptic techniques, using one 3 mL Vacutainer® collection tube (red top) without anticoagulant per participant or three 0.25 mL sterile capillary tubes that were expressed into screw-top plastic vials. All samples were taken at the home of the participant, labeled, and stored in small portable ice chests until they were transported to our field laboratory within 4 hrs. Blood samples were centrifuged for 10 minutes at 3,000 rpm at 4°C, and sera were then transferred to cryovials and stored at −70°C, until transported on dry ice to the NMRCD laboratory facilities in Lima for serological testing for DENV antibodies.

### Plaque Reduction Neutralization Tests (PRNT)

All blood samples from the longitudinal arm of the study were assayed by plaque reduction neutralization test (PRNT) using 70% reduction for the cut-offs (PRNT_70_). PRNT_70_ were carried out at final serum dilutions of 1∶60 and 1∶120 (after addition of virus) for DENV-1 and DENV-2 and 1∶30 and 1∶60 for DENV-3. For DENV-2 linear regression models were fit to estimate the percent reduction at cut-off dilution of 1∶80, whereas a cutoff dilution of 1∶60 was used for DENV-1 and DENV-3. Because DENV-3 was not detected in Iquitos until December 2001, routine screening of samples for DENV-3 neutralizing antibody (NtAb) was not initiated for sera collected until after January 2002. For participants who may have seroconverted to DENV-3 before 2002, prior samples were tested for DENV-3 sequentially until a sample negative for DENV-3 was observed. DENV-4 circulation was not detected in Iquitos between 1993 through the end of this cohort study [14]. We did not, therefore, test for DENV-4 antibody.

PRNTs were performed as described by Sangkawibha [21] and Graham [22], with a modified protocol for semi-micro methods with baby hamster kidney (BHK-21) cells (clone 15) in 12 or 24 well plates [23]. Modifications are described in detail by Kochel et al. [8] and Comach et al. [24]. Briefly, 0.2 mL of diluted test sera were mixed with 0.2 mL diluted media (Earle's minimal essential medium [E-MEM], 2% fetal bovine serum [FBS], and antibiotic/antimycotic) containing 40–80 PFU of assay virus and then incubated at 4°C for 15 hours. Unless otherwise stated, DENV strains utilized in the PRNT were two viruses isolated in Thailand in 1974 from DHF cases [25], DENV-1 16007 and DENV-2 16681, as well as a 2001 Peruvian isolate (from a DF case) of DENV-3, IQD1728. Prior to use in the assay, viruses were amplified in *Ae. albopictus* C6/36 cell culture and aliquots frozen at −70°C to ensure consistency in testing throughout the study. Sera were heat-inactivated at 56°C for 30 minutes before the PRNT. In triplicate, 0.1 mL of virus-serum mixture (dengue-1: 16007; dengue-2: 16681; dengue-3: IQT1728) was added to 0.5 mL media containing 1.5×10^5^ BHK21 cells and then added to a well of a 24 well tissue culture plate and incubated at 37°C with 5% CO_2_ for 3 hrs. The cells were then overlaid with 0.5 mL of overlay media (0.6% carboxymethyl Cellulose, MEM w/o phenol Red, 10% FBS, 0.075% NaHCO_3_ and antibiotic/antimycotic) and incubated at 37°C with 5% CO_2_ for 5 days. The media was removed, and the cells rinsed with H_2_O and stained with 0.5 mL/well stain solution (0.1% (w/v) Naphthol Blue Black, 1.36% (w/v) Sodium Acetate, and 6% (v/v) Glacial Acetic Acid) for 30 min. Stain was removed and plaques were counted. Results were expressed as the serum dilution, determined by linear regression analysis, that reduced the number of plaques by 70% compared to that of normal human serum at the same dilution. Positive and negative human control sera were included with every batch of sera tested. Cutoff values, established based on the ability of the assay to maximize serotype sensitivity and specificity, were 1∶60 for DENV-1 and DENV-3 and 1∶80 for DENV-2 (Minnick SL, unpublished data).

#### Interpretation of PRNT results

PRNT is the most specific serological test for dengue infections, but a number of factors influence its sensitivity and specificity, including cross-reactions among antibodies to different dengue serotypes [8], [26], [27] (Minnick et al. unpublished data). To account for potential errors in the interpretation of PRNTs for a single blood sample, we determined seroconversions (SC) by considering serological profiles derived from the full sequence of blood samples for each participant. The following criteria were used to identify serotype-specific seroconversions. A seroconversion was scored when the percent increase in reduction between a negative sample and a subsequent sample was greater than 20% and results from later samples were consistently positive (e.g., ---+++). When subsequent PRNT results were not consistent, a seroconversion was not scored. These were considered false-positives (FP; e.g., --+--). Isolated reversions were considered false negatives (FN; e.g., --++-++). False-positive/false-negative rates were used to calculate correction factors to estimate the true number of seroconversions among participants who showed indication of seroconversion in the final sample of a series (e.g., ---+) for incidence rate estimates. Correction factors (Table 1) were calculated as the number of true-positives (TP) over the total number of apparent seroconversions, TP/(TP + FP).

**Table 1:** Correction factors for estimating true number of seroconversions for participants who have evidence of seroconversion in a single post-conversion sample.

Profile	FALSE+	TRUE+	TOTAL	Correction Factor
N-D1	75	28	103	0.2718
N-D2	43	11	54	0.2037
N-D3	0	94	94	1.0000
N-D12	43	11	54	0.2037
N-D13	0	14	14	1.0000
N-D123	3	15	18	0.8333
D1-D12	104	59	163	0.3620
D1-D13	0	13	13	1.0000
D1-D123	3	49	52	0.9423
D2-D23	0	26	26	1.0000
D2-D12	98	54	152	0.3553
D2-D123	7	64	71	0.9014
D3-D13	14	0	14	0.0000
D3-D23	1	0	1	0.0000
D3-D123	7	0	7	0.0000
D12-D123	218	318	536	0.5933
D13-D123	5	1	6	0.1667
D23-D123	2	2	4	0.5000
D**1**2*-D123	1	24	25	0.9600
D1**2** **-D123	1	10	11	0.9091

*: Participants positive for DENV-1 or DENV-1/-2 in first sample.

**: Participants positive for DENV-2 or DENV-1/-2 in first sample. In both cases participants did not have DENV-3 above thresholds levels in samples previous to seroconversion. For incidence calculations these individuals were considered monotypic.

N = negative, D1 = DENV-1, D2 = DENV-2, D3 = DENV-3, D12 = DENV-1 and -2, D13 = DENV-1 and -3, D23 = DENV-2 and -3, D123 = DENV-1, -2 and -3, D**1**2^*^ = DENV-1 or DENV-1 and -2, D12^**^ = DENV-2 or DENV-1 and -2.

### Febrile Surveillance

If a study participant was observed to have a febrile illness during school-based surveillance activities, acute and convalescent blood samples were collected. Acute-phase serum samples were tested for DENV infection either by virus isolation in cell culture or by detection of viral RNA by reverse transcription polymerase chain reaction (RT-PCR). Both acute- and convalescent-phase samples were screened for anti-DENV IgM antibody by IgM-capture enzyme-linked immunosorbant assay (ELISA). In addition, a subset of acute and convalescent samples (those collected prior to 2002 and a subset of those collected during 2004) was screened for anti-DENV IgG antibodies by ELISA. Febrile episodes were classified as DENV infections based on the isolation of virus, RT-PCR, IgM serology (elevated IgM antibody titers [≥1∶100] in the acute sample, convalescent sample, or both), or IgG antibody serology (four-fold rise in titers between acute and convalescent samples). Infections identified by viral detection, IgM seroconversion or an elevated IgM titer of ≥1∶400 were counted as seroconversions in all incidence calculations, even if pre- and/or post-sample PRNT data was not available or discordant, whereas individuals with an elevated IgM titer of 1∶100 or IgG serology were only included for incidence calculations if confirmatory PRNT data was available.

#### IgM-capture ELISA

Dengue-specific IgM antibody titers were determined by an IgM-capture ELISA adapted from published protocols [28]. Briefly, plates (96-well format) were coated with anti-human IgM antibody to capture participant IgM antibody. Virus-specific IgM was detected by the addition of dengue viral antigen, followed by virus-specific hyperimmune ascitic fluid and horseradish peroxidase (HRP)-labeled anti-mouse IgG. Following the addition of colorimetric substrate, plates were read at 410 nm. All acute- and convalescent-phase samples were initially screened at a 1∶100 dilution. Samples exceeding the reference cut-off value, calculated as the mean of seven antibody-negative samples plus three standard deviations, were considered IgM antibody-positive. Positive samples were subsequently re-tested at four-fold serial dilutions to determine end-point antibody titers.

#### IgG-ELISA

Dengue-specific IgG titers were determined by an IgG ELISA adapted from Ansari et al. [29]. Plates (96-well format) were coated with DENV antigen (cocktail of serotypes 1, 2, 3 & 4) produced from infected Vero cell culture lysates or uninfected cell lysates as controls. Aliquots of diluted participant's serum samples (1∶100) were added to two dengue antigen coated wells and to two control wells. After addition of HRP-conjugated mouse anti-human IgG, OD values were recorded at 410 nm. Adjusted OD values were calculated by subtracting the OD of the uninfected antigen coated well from that of the corresponding viral antigen coated well. The cutoff OD value for determining antibody positivity was calculated as the mean adjusted OD plus 3 standard deviations of antibody negative control sera.

#### RT-PCR

Viral RNA was prepared from 140 µl of each sera sample using QIAamp Viral RNA Mini Kits following the manufacturer's instructions (Qiagen Inc., Valencia, CA 91355). Nested DENV RT-PCR was performed following the protocol of Lanciotti et al. [30] on serum samples for dengue viral RNA detection.

#### Virus isolation

Isolation of DENV serotypes was attempted on all acute serum specimens. Using standard virological techniques, cell cultures (C6/36 and Vero cells) were inoculated with serum and monitored for cytopathic effects. All cells inoculated for virus isolation were tested by immunofluorescence assay using DENV serotype-specific monoclonal antibodies whether or not the cells show cytopathic effects.

### Statistical Analyses

Proportions were compared using a chi-square test using the FREQ procedure in SAS (SAS Version 8, 1999, SAS Institute Inc., Cary, NC.) with statistical significance assessed at an alpha level of 0.05. We calculated seroprevalence rates from serostatus in the first blood sample of all participants enrolled through September 1999 when an active cohort of 2,400 participants was achieved as interpreted through examination of complete serological profiles (see Interpretation of PRNT results above).

#### Incidence rate calculations

Except for clinically apparent cases, we were unable to identify the time of infection between paired blood draws taken at ca. 6 month intervals. We thus calculated seroincidence rates and 95% CI for 10 time periods between January 1999 and February 2005 assuming that infection occurred on the mid-date of the monitoring interval. Thus the incidence rate for a time interval was the total number of seroconversions with mid-dates occurring within the time period (numerator) divided by the sum of person-days contributed each participant during the time period (denominator). Serotype-specific incidence rates only included person-days of participants who were susceptible to that serotype at the start of the monitoring interval. Calculations assuming that infections occurred at the end of each monitoring interval were qualitatively and quantitatively similar to those calculated assuming that infection occurred on the mid-date of the interval; only a temporal shift was observed (Tables S3, S4, S5). Maps and GIS information were managed using Arc-View 3.2 (ESRI, Redlands, CA).

## Results

Between February 21, 1999 and February 12, 2005 to maintain an active cohort of 2,400 study participants, a total of 4,586 participants were enrolled in the study. Seventy-five percent were enrolled between February 1999 and March 2000 (Figure 1, Table S1). Of all participants, 683 (14.9%) individuals were lost to follow up after providing a single baseline sample, whereas the remaining 3,903 (85.1%) participants provided between 2 and 14 samples (Figure 1). Fifty-two percent (n = 2,383) and 31.5% (n = 1,445) of the participants provided ≥4 and ≥7 blood samples, respectively.

**Figure 2:**
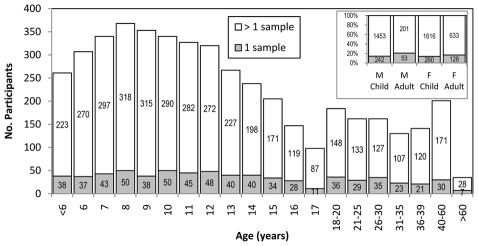
Number of participants enrolled in a 1999–2005 cohort by age. The percentage of enrollees providing >1 blood sample was consistent among age groups (78–87%, *P* = 0.17).

The majority of participants (77.9%; n = 3,571) were younger than 18 years of age. Females were more common than males, representing 57.5% (n = 2,637) of the overall study population. Among adult participants, female participation (75.0%) was significantly higher than for males (*P*<0.0001). Study participants providing single or multiple blood draws had a comparable distribution by gender (*P* = 0.7), but the proportion of adult participants among those lost to follow up after providing a single sample was slightly higher than among participants who provided multiple samples (*P*<0.003, Figure 2). The ages of enrollees were evenly distributed among school age children (77.9% of cohort population) and adults except for the >60 years group and was consistent between participants who provided multiple samples and those dropping out after a single sample (*P* = 0.17, Figure 2). Participants were recruited from 36 to 60 city blocks in each of 8 geographic zones (Table 2). The proportion of participants leaving the study after a single blood draw ranged from 10.6 to 19.9% (*P*<0.0001) among the 8 zones.

**Table 2:** Number of participants enrolled in 1999–2005 cohort by geographic zone.

Zone	No. Enrolled	No. Blocks	Average No. persons per block (SE)/Range	% Enrollees with >1 blood sample	% Enrollees with anti-DENV antibody during 1999 baseline	
					1 sample	>1 sample
BG	559	40	14.0 (2.6)/1-70	80.1	82.0	65.6
IQ	406	36	11.3 (2.7)/1-98	88.2	84.6	80.8
MC	485	53	9.2 (0.9)/1-30	85.6	78.0	87.1
MY	639	60	10.7 (1.4)/1-52	89.4	96.6	90.2
PT	479	48	10.0 (1.3)/1-34	86.0	83.3	72.4
PU	617	50	12.3 (1.6)/1-46	80.1	83.8	84.8
SA	499	51	9.8 (1.5)/1-42	81.2	68.2	87.0
TA	902	54	16.7 (2.1)/1-87	88.7	83.9	69.8

School-based surveillance was carried out on a subset of 1,000–1,180 cohort participants each year (Table S6) between the ages of 5 and 18, with slightly more female (53.4%) than male enrollees.

### PRNT Assay

Although the time interval between blood samples was relatively long (∼6 mo), we observed cross-reactive antibody in the first and sometimes second post-conversion blood samples, which became specific in subsequent samples (e.g. [N-N-D12-D1-D1], see Table 1 for sequence abbreviations). The rate of apparent cross-reaction was similar for primary (18.8%, 29/154) and secondary infections (23.9%, 33/138).

Correction factors for all observed infection sequences are shown in Table 1. Of the 22 participants whose baseline PRNT sample showed evidence of monotypic infection with DENV-3 followed by an apparent infection to either DENV-1 or DENV-2, none had supportive PRNT results in subsequent samples (correction factor = 0 in Table 1). Overall, profiles indicating seroconversion were reliable for new DENV-3 infections (correction factor>0.90), but less so for new DENV-1 and DENV-2 infections (correction factor<0.37). Correction factors decreased for participants with a polytypic pre-conversion status, but these seroconversions held up >50% of the time for new DENV-3 infections. In a number of cases, pre-conversion blood samples presented a % reduction that was between 1 and 10% below the cutoff, followed by a 20% increase in the subsequent sample. In other instances, serology suggested infection with DENV-3, only to fall off in subsequent samples. According to our criteria, none of these cases would be considered unequivocal seroconversions; although they could be true seroconversions our assay was not sensitive enough to detect them. Estimates of incidence with and without these possible seroconversions did not have any significant effect on the outcome (see Tables S3, S4, S5).

### Seroprevalence

Overall, we were able to interpret baseline serostatus for 99.9% of study participants. For all participants enrolled before October 1999 (*n* = 2,527) only 19.7% (*n* = 498) did not have serological evidence of prior dengue infection. Approximately half the participants had DENV NtAbs against both DENV-1 and DENV-2 (1,292, 51.1%) at the baseline sampling period of the study; 366 (14.5%) and 368 (14.6%) had monotypic NtAbs to DENV-1 and DENV-2, respectively. DENV NtAbs were observed at the same rate in participants providing a single (286 of 350, 81.7%) or multiple (1,740 of 2,174, 80.0%) blood draws (*P* = 0.4642).

**Figure 3:**
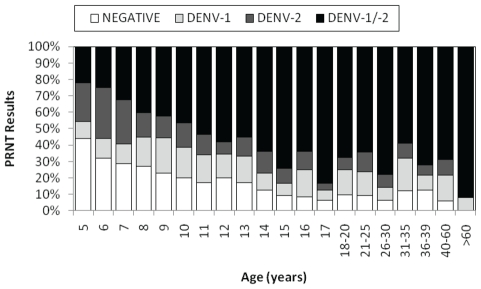
Age-specific seroprevalence rates for participants enrolled in the longitudinal cohort between February and September 1999.

**Figure 4:**
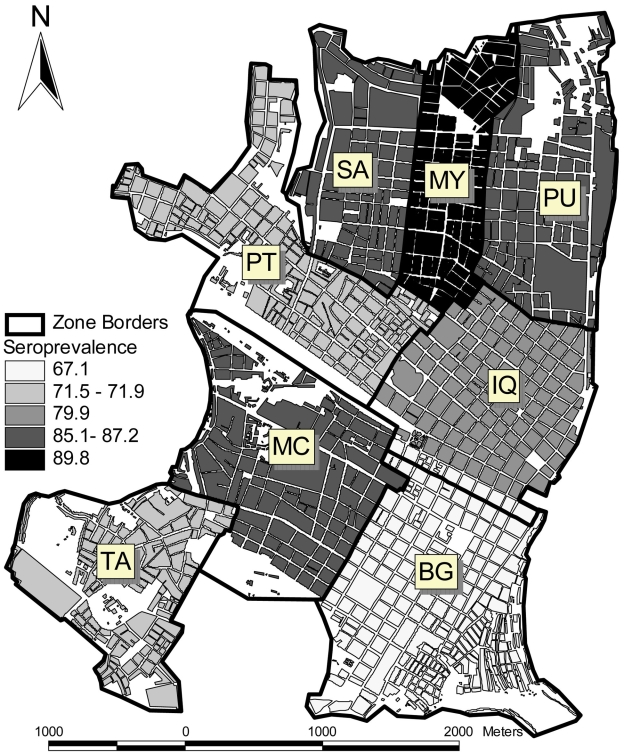
Seroprevalence rates for cohort participants enrolled between February and September 1999 in 8 geographic zones in Iquitos, Peru.

Prevalence of DENV NtAbs was higher among females (1,170 of 1,424, 82.2%) than males (856 of 1,100, 77.8%; *P* = 0.0065) and increased with age. Of adults (≥18 years) prevalence was 91.2% (423 of 464), while among children (<18) it was 77.8% (1,603 of 2,060; *P*<0.0001). Over half of the youngest group (5 year-olds) presented evidence of DENV NtAbs (56.1%) and this proportion increased steadily with age with more than 87% of the participants ≥14 year-olds showing a history of infection (Figure 3, *P*<0.0001). DENV seroprevalence rates varied geographically (Figure 4). Age-adjusted rates ranged from 67.1 to 89.9% (*P*<0.0001) with lower rates in zones (BG, PT, TA) where some households were located in river-front areas that are seasonally flooded.

### Seroincidence

Of the 3, 903 participants who provided at least 2 blood samples, 2,542 (65.1%) had no change in their serostatus compared to baseline over the observation period (Table 3). A total of 1,414 DENV seroconversions occurred during the study period among 1,347 participants (34.5% of the cohort); 1,281 participants showed evidence of infection with a single serotype, 65 to 2 serotypes, and 1 to 3 serotypes. Of these 1,414 seroconversions, 585 (41.4%) were in the final blood sample, which cannot be conclusively determined to be true seroconversions, and 62 (1.6%) were ambiguous (see *PRNT*, above) (Table 4 and S2). Complete result profiles from 14 participants were excluded because they could not be interpreted. The majority of infections were secondary (75.7%, Table 4). Prior to invasion by DENV-3 the ratio of the incidence of primary to secondary infections was less than two –fold, then increased to more than five-fold through the peak of epidemic transmission (Table 5). By 2004, the rate of dengue infection in primary and secondary infections was nearly equal. DENV-3 was responsible for 58.4% of infections and most likely responsible for an additional 18.8% (cross reactive responses that included DENV-3 antibody) of all the DENV infections observed (Table 4). The exact infecting serotype could not be identified in 24.9% of the primary infections (see Table 4; N-D12, N-D13, N-D23, N-D123) and 17.9% of the secondary infections (see Table 4; D3-D123, D2-D123, D1-D123).

**Table 3:** Summary of serological results for 3,903 participants (15,780 monitoring intervals) providing ≥2 blood samples in longitudinal cohort study, Iquitos, Peru 1999–2005.

Status		No.	%	Intervals				
				Total	Consistent^a^	False (+)		False (−) 1 interval^d^
						1^b^	2^c^	
**No Seroconversion**								
Negative	N-N	585	23.0	2833	2435 (86.0%)	14.0%	1.4%	
Monotypic		620	24.4	3326				
	D1-D1	285	11.2	1429	1058 (74.0%)	20.6%	2.0%	5.3%
	D2-D2	295	11.6	1696	1290 (76.1%)	17.9%	1.2%	6.1%
	D3-D3	40	1.6	201	124 (61.7%)	34.3%	5.4%	4.0%
Polytypic		1337	52.6	8066				
	D12-D12	1144	45.0	6393	5127 (80.2%)	8.0%	1.9%	11.8%
	D13-D13	8	0.3	61	16 (26.2%)	49.2%	8.1%	24.6%
	D23-D23	7	0.3	53	10 (20.4%)	77.6%	22.4%	10.2%
	D123-D123	97	3.8	936	739 (79.0%)	-	1.9%	21.0%
	D**1**2-D**1**2	57	2.2	443	421 (95.0%)	2.0%	0.2%	2.9%
	D1**2**-D1**2**	24	0.9	184	166 (90.2%)	2.2%	1.9%	7.6%
Total		2542	65.1	14229	11386 (80.0%)			
**Seroconversions**								
	Single	1281	32.8	1281				
	Double	65	1.7	130				
	Triple	1	0.03	3				
Total		1347	34.5	1414				

Serostatus of each study subject (No.) either remained consistent for all monitoring intervals throughout their participation (No seroconversion) or showed serological evidence of 1, 2 or 3 DENV infections (seroconversions). Top panel shows the number of participants and monitoring intervals where no serological changes were observed, demonstrating the percentage of sampling intervals where antibodies to other DENV serotypes were observed (false+/cross-reactive antibodies) for 1 or 2 monitoring intervals or where titers fell below threshold values for a single interval. Lower panel shows the number of seroconversions observed.

a All PRNT results were the same, e.g., [N-N-N or N-N-N-N or D1-D1-D1-D1 or D12-D12-D12-D12-D12].

b e.g. [N-N-D2-N-N-N or D1-D12-D1-D1-D1].

c e.g. [N-D2-D2-N-N-N or D2-D12-D12-D2-D2].

d e.g. [D2-D2-N-D2-D2 or D12-D12-D1-D12-D12].

**Table 4:** Summary of 1,414 seroconversions detected among 1999–2005 longitudinal cohort participants by PRNT in Iquitos, Peru.

Status			Seroconversions			
			Unequivocal		Putative^a^	
			No.	%	No.	%
1° Infections		N-D1	23	1.63	33	2.33
		N-D2	10	0.71	8	0.57
		N-D3	94^b^	6.65	60	4.24
		N-D12	11	0.78	24	1.70
		N-D13	14	0.99	23	1.63
		N-D23	2	0.14	3	0.21
		N-D123	15	1.06	17	1.20
		**ALL 1°**	**169**	**11.95**	**168**	**11.88**
2° Infections	DV-1	D2-D12	45	3.18	28	1.98
		D3-D13	0	0.00	7	0.50
		D23-D123	2	0.07	4	0.28
		**ALL D1**	**47**	**3.89**	**39**	**2.76**
	DV-2	D1-D12	54	3.82	45	3.18
		D3-D23	0	0.00	1	0.07
		D13-D123	1	0.07	0	0.00
		**ALL D2**	**55**	**3.89**	**46**	**3.25**
	DV-3	D1-D13	13	0.92	10	0.71
		D2-D23	26	1.84	8	0.57
		D12-D123	318^c^	22.49	209^d^	14.78
		D**1**2-D123	24	1.70	14	0.99
		D1**2-**D123	8	0.57	6	0.42
		**ALL D3**	**389**	**27.51**	**247**	**17.47**
	DV-1 or DV-2	D3-D123	0	0.00	6	0.42
	DV-1 or DV-3	D2-D123	63^e^	4.46	35	2.48
	DV-2 or DV-3	D1-D123	44^f^	3.11	44	3.11
		**ALL 2°**	**598**	**42.29**	**417**	**29.49**
		TOTAL	767	54.24	585	41.37

a Seroconversion occurring in final pair of samples from a participant (eg. [N-N-N-D1] or [D1-D1-D1-D12]). For incidence calculations numbers will be multiplied by a correction factor calculated based the proportion of specific pair profiles (i.e. N to D1, D1 to D12, D12 to D123) observed that are maintained in subsequent samples (ie., N-D1-D1 or D1-D12-D12) versus those that represent false+ results (i.e. N-D1-N or D1-D12-D1).

b 1 with 2^nd^ sample 8/12/2001.

c 14 participants with 2^nd^ sample dates in 2001 (July = 3; October = 6; November = 1; December = 4 ).

d 9 participants with 2^nd^ sample in 2001 (August = 1; October = 2; December = 6).

e 2 participants with 2^nd^ samples on 5/27/01 and 10/26/01.

f 1 participant with 2^nd^ sample 10/25/2001.

**Table 5:** Seroincidence of primary and secondary infections for all DENV serotypes between February 1999–February 2005.

Date	Primary Infections		Secondary Infections	
	Serotype-Adjusted	Population-based	Serotype-Adjusted	Population-based
2/99–3/00	2.73 (2.52–2.93)	0.53 (0.51–0.55)	4.34 (4.17–4.51)	1.47 (1.42–1.53)
4/00–5/01	3.48 (3.25–3.71)	0.66 (0.64–0.68)	6.38 (6.17–6.59)	1.96 (1.90–2.02)
6/01–12/01	3.90 (3.47–4.33)	0.69 (0.66–0.72)	23.04 (21.86–24.22)	4.80 (4.58–5.02)
1/02–4/02	12.11 (10.43–13.78)	2.68 (2.51–2.85)	63.95 (59.30–68.59)	17.37 (16.27–18.48)
5/02–8/02	17.77 (15.64–19.89)	3.52 (3.35–3.70)	147.59 (139.00–156.18)	34.28 (32.55–36.00)
9/02–12/02	39.89 (35.45–44.34)	8.66 (8.21–9.10)	110.89 (103.62–118.16)	30.27 (28.72–31.82)
1/03–4/03	14.11 (12.21–16.00)	2.79 (2.62–2.95)	36.67 (33.69–39.65)	11.35 (10.67–12.03)
5/03–8/03	3.33 (0.07–6.60)	0.48 (0.34–0.63)	17.54 (10.52–24.56)	4.95 (3.48–6.41)
**9/03–5/04**	**5.68 (4.42–6.94)**	**0.92 (0.83–1.01)**	**20.87 (18.17–23.56)**	**5.84 (5.28–6.40)**
**8/04–2/05**	**23.54 (19.96–27.12)**	**7.79 (7.10–8.48)**	**28.34 (24.79–31.90)**	**14.18 (12.92–15.44)**

Bolded rows include school-based component only. Seroincidence rates were calculated under the assumption that infection occurred at the midpoint of the sampling interval. Incidence rates were adjusted based on susceptibility patterns (sero-type adjusted) and for the entire study population (population-based).

Among the febrile children captured through active school absence monitoring, 73 confirmed DENV infections were identified; 20 by virus isolation, 14 by RT-PCR, and 39 by IgM serology (Table 6). Another 42 participants had symptoms consistent with DF and PRNT evidence for a seroconversion during the monitoring interval of their febrile episode. Of these 11 with an IgM antibody titer of 1∶100 and 3 with a 4-fold rise in anti-DENV IgG antibody titer between acute and convalescent samples were included in the calculation of symptomatic dengue infections. In total, we identified 11 DENV-1 cases (7 primary, 4 monotypic to polytypic), 2 DENV-2 cases (1 primary, 1 monotypic to polytypic), and 74 DENV-3 cases (22 primary, 20 monotypic to polytypic, 32 DENV-1/-2 to DENV-1/-2/-3). For 5 primary and 23 secondary infections the infecting serotype could not be identified.

**Table 6:** Summary of dengue cases identified through febrile case surveillance in school-based cohort between June 2000 and December 2004.

Status		Diagnostic Assay					
		Viral Detection	IgM SC	Elevated IgM^b^	IgG SC^c^	Clinical w/PRNT^d^	Total
DENV-1	1°	2	2	-	-	3	7
	2°	1	-	1	-	2	4
DENV-2	1°	-	1	-	-	-	1
	2°	-	1	-	-	-	1
DENV-3	1°	9	2	9	-	2	22
	2°						
DENV-1/-3	1°2°	-22	-10	17	-3^a^	-10	152
		-	3	3	-	2	8
DENV-2/-3	1°	-	-	-	-	-	-
	2°	-	3	1	-	-	4
DENV-1/-2/-3	1°	-	2	-	-	1	3
	2°	-	-	-	-	-	-
Unknown	1°	-	-	1	-	-	2
	2°	-	-	1	7	-	8
	UK	-	1	1	1	-	3
Total	1°	11	7	2	-	6	35
	2°	23	17	13	10	14	77
	UK	-	1	1	1	-	3

a All 3 individuals were febrile in late July 2001.

b Includes 11 participants with an elevated IgM titer of 1∶100 with supporting PRNT data indicating seroconversion during sampling interval.

c 3 of 10 participants with supporting PRNT data indicating seroconversion were included for incidence calculation.

d None in this category was included for incidence calculations.

### Temporal Trends

During the first 15 months of the study, DENV-1 and DENV-2 co-circulated at low infection rates, with approximately 2 and 6 infections per 100 people per year in the entire population and susceptible population, respectively (Table 7). DENV-3 appears to have been introduced into Iquitos sometime in 2001. The first DENV-3 isolate was recovered from a DF case on December 7, 2001 [13](TJK, unpublished results). We detected only 2 clinical DENV-3 cases in our school surveillance study prior to January 2002. In addition, 2 febrile participants detected in July 2001 had no detectable IgM response, but did show a 4-fold rise in IgG titer. Both had NtAb to DENV-3 in 2002 PRNT results. Examination of PRNT antibody results indicates possible DENV-3 transmission at least 5–6 months earlier than the first isolate. First evidence of DENV-3 transmission was in a sample taken from a 5 year-old female whose last PRNT sample taken on May 27, 2001 indicated seroconversion to this serotype, but no subsequent samples were taken to confirm this result. The next earliest indications of transmission were in samples taken July 24 from a 12 year-old male and July 27 from a 20 year-old female. The previous blood samples taken at the end of 2000 from both of these participants were negative for DENV antibody, thus indicating that infection took place sometime between December 2000 and late July 2001.

**Table 7:** Serotype- specific DENV incidence between February 1999–February 2005.

Date	Serotype-adjusted (95% CI) and [Population-based (95% CI)] Seroincidence per 100 person-years at risk							
	DV-1	DV-2	DV-3	DV-1/-2	DV-1/-3	DV-2/-3	DV-1/-2/-3	TOTAL
2/99–3/00	2.22 (2.09–2.35) [0.76 (0.74–0.79)]	3.46 (3.26–3.65) [1.15 (1.11–1.19)]	0.00 (0.00)	0.47 (0.44–0.51) [0.09 (0.09–0.09)]	0.00 (0.00)	0.00 [0.00]	0.00 [0.00]	6.15 (5.93–6.36) [2.00 (1.93–2.07)]
4/00–5/01	3.05 (2.90–3.21) [1.01 (0.98–1.04)]	3.46 (3.28–3.63) [1.13 (1.10–1.16)]	0.22 (0.21–0.22) [0.22 (0.21–0.22)]	1.01 (0.94–1.08) [0.19 (0.19–0.20)]	0.21 (0.20–0.22) [0.07 (0.07–0.07)]	0.00 [0.00]	0.00 [0.00]	7.95 (7.72–8.19) [2.62 (2.54–2.70)]
6/01–12/01	2.75 (2.53–2.97) [0.90 (0.86–0.94)]	1.26 (1.16–1.37) [0.39 (0.37–0.41)]	3.90 (3.72–4.09) [3.89 (3.71–4.07)]	0.21 (0.18–0.23) [0.04 (0.03–0.04)]	0.83 (0.76–0.90) [0.27 (0.26–0.28)]	0.29 (0.27–0.31) [0.09 (0.09–0.09)]	0.00 [0.00]	9.24 (8.81–9.67) [5.58 (5.32–5.84)]
1/02–4/02	7.15 (6.40–7.89) [2.85 (2.67–3.03)]	1.50 (1.34–1.66) [0.57 (0.53–0.61)]	13.13 (12.29–13.98) [12.94 (12.11–13.76)]	0.77 (0.66–0.87) [0.17 (0.16–0.18)]	6.29 (5.64–6.95) [2.51 (2.35–2.67)]	4.46 (3.99–4.93) [1.70 (1.59–1.80)]	1.53 (1.32–1.74) [0.34 (0.32–0.36)]	34.83 (32.59–37.07) [21.07 (19.73–22.42)]
5/02–8/02	1.16 (1.05–1.27) [0.39 (0.37–0.40)]	2.02 (1.84–2.20) [0.66 (0.62–0.69)]	33.82 (32.03–35.6) [30.64 (29.10–32.18)]	0.63 (0.55–0.70) [0.13 (0.12–0.14)]	13.05 (11.85–14.25) [4.23 (4.01–4.44)]	7.26 (6.59–7.92) [2.28 (2.17–2.40)]	1.95 (1.72–2.19) [0.39 (0.37–0.41)]	59.89 (56.75–63.02) [38.70 (36.76–40.65)]
9/02–12/02	3.06 (2.79–3.34) [1.00 (0.95–1.05)]	4.81 (4.41–5.22) [1.64 (1.56–1.73)]	37.72 (35.55–39.89) [29.64 (28.12–31.15)]	1.53 (1.37–1.70) [0.35 (0.34–0.37)]	17.64 (16.00–19.29) [5.41(5.14–5.69)]	15.10 (13.78–16.42) [4.84 (4.59–5.09)]	9.30 (8.26–10.34) [2.02 (1.91–2.12)]	89.18 (84.14–94.22) [44.91 (42.61–47.21)]
1/03–4/03	1.50 (1.35–1.65) [0.55 (0.52–0.59)]	0.93 (0.83–1.02) [0.33 (0.31–0.35)]	17.71 (16.40–19.02) [11.62 (10.93–12.32)]	1.57 (1.39–1.76) [0.40 (0.38–0.42)]	10.86 (9.67–12.05) [3.24 (3.05–3.43)]	7.80 (6.95–8.66) [2.21 (2.08–2.35)]	2.38 (1.97–2.58) [0.45 (0.42–0.48)	42.65 (39.69–45.61) [18.81 (17.69–19.94)]
5/03–8/03	0.00 (0.00)	0.00 (0.00)	8.00 (4.58–11.42) [5.18 (3.65–6.71)]	1.98 (0.75–3.21) [0.48 (0.34–0.63)]	10.88 (2.82–18.93) [2.14 (1.50–2.77)]	0.00 (0.00)	0.00 (0.00)	20.86 (13.13–28.58) [7.80 (5.49–10.10)]
**9/03–5/04**	**0.28 (0.24–0.32) [0.10 (0.09–0.11)]**	**0.37 (0.31–0.42) [0.13 (0.12–0.14)]**	**8.40 (7.37–9.43) [4.97 (4.49–5.44)]**	**0.16 (0.13–0.19) [0.04 (0.03–0.04)]**	**4.60 (3.78–5.42) [1.18 (1.06–1.29)]**	**5.49 (4.49–6.50) [1.38 (1.24–1.51)]**	**3.73 (2.90–4.55) [0.60 (0.55–0.66)]**	**23.02 (20.45–25.60) [8.39 (7.58–9.20)]**
**6/04–2/05**	**3.95 (3.49–4.42) [2.21 (2.02–2.41)]**	**0.84 (0.74–0.93) [0.47 (0.42–0.51)]**	**21.42 (19.12–23.73) [14.13 (12.87–15.38)]**	**0.92 (0.80–1.04) [0.41 (0.38–0.45)]**	**13.60 (11.76–15.44) [5.65 (5.14–6.15)]**	**7.90 (6.84–8.96) [3.32 (3.03–3.62)]**	**2.76 (2.34–3.18) [0.91 (0.83–0.99)]**	**51.39 (46.41–56.36) [27.10 (24.69–29.51)]**

Bold rows include school-based component only. Seroincidence rates were calculated under the assumption that infection occurred at the midpoint of the sampling interval.

During the first trimester of 2002 we observed increased DENV-1 and DENV-3 transmission, with a steady increase in the incidence of DENV-3 infection from12.9 to 29.6 seroconversions per 100 p-years at risk between the first and last trimester of 2002, corresponding to a major dengue outbreak in Iquitos. Transmission rates of DENV-3 decreased steadily through the end of August 2003 when our community-based study ended. A subset of our study cohort was followed in school surveillance from September 2003 through February 2005. In this later group seroconversion rates were consistent with the May–August 2003 trimester through May 2004 but increased dramatically in the last half of 2004, which corresponded to another dengue outbreak.

**Figure 5:**
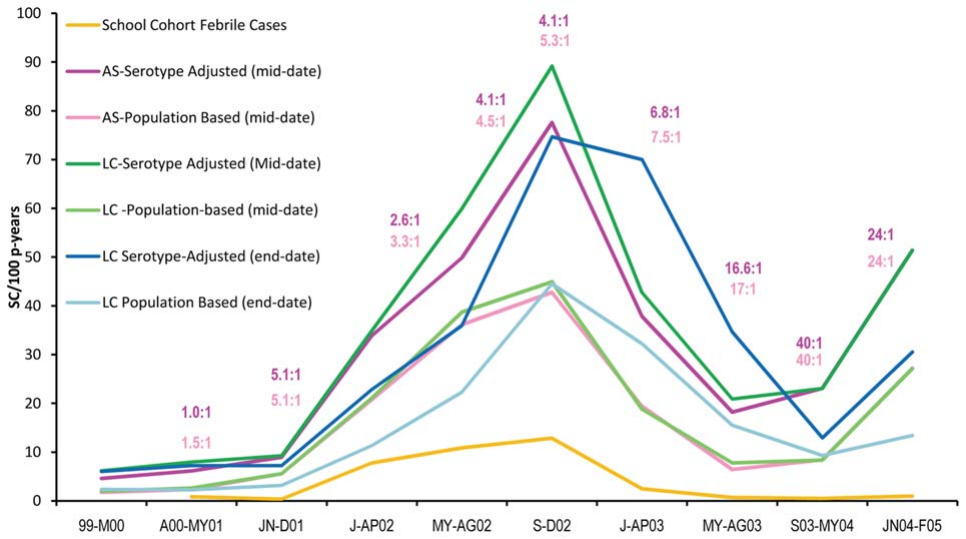
Comparison of DENV seroconversion and case incidence rates between January 1999 and February 2005. Incidence rates are expressed as cases or seroconversions per 100 person-years at risk. Symptomatic-to-asymptomatic case ratios were calculated from incidence of active dengue cases within the School surveillance program (yellow line) compared with overall seroconversion rates within susceptible (purple line) or the entire school cohort (pink line). The ratios are shown above the curves for each time period for the susceptible (purple) and entire (pink) population. Seroconversion rates assuming infection took place at the mid-point (green) or end-point (blue) of the monitoring interval are shown for the entire longitudinal cohort calculated for both the susceptible (dark) and entire (light) populations.

When seroconversion rates were calculated for the subset of the longitudinal cohort enrolled in the school surveillance program, they were similar to those for the entire cohort, except for May to December 2002 when seroconversion rates appeared about 10 SCs per100 p-years less in the school cohort when adjusted for the susceptibility patterns of the population (Table 8, Figure 5, pink line compared to green). Population-based seroconversion rates were nearly identical. Incidence of symptomatic DF cases in the school cohort was significantly lower than the seroconversion rate in the sample population, ranging from 0.6 to 13 cases per 100 p-years (Figure 5).

**Table 8:** Serotype- specific DENV incidence in 1,100 person school surveillance cohort between February 1999–August 2003.

Date	Serotype-adjusted (95% CI) and [Population-based (95% CI)] Seroincidence per 100 person-years at risk							
	DV-1	DV-2	DV-3	DV-1/-2	DV-1/-3	DV-2/-3	DV-1/-2/-3	TOTAL
2/99–3/00	1.71 (1.57–1.84) [0.68 (0.65–0.72)]	2.80 (2.56–3.03) [1.04 (0.99–1.10)]	0.00 [0.00]	0.11 (0.10–0.12) [0.02 (0.02–0.03)]	0.00 [0.00]	0.00 [0.00]	0.00 [0.00]	4.61 (4.37–4.86) [1.75 (1.66–1.84)]
4/00–5/01	2.76 (2.57–2.95) [1.07 (1.02–1.12)]	2.85 (2.65–3.06) [1.02 (0.98–1.07)]	0.16 (0.15–0.17) [0.16 (0.15–0.17)]	0.37 (0.34–0.41) [0.08 (0.08–0.08)]	0.00 [0.00]	0.00 [0.00]	0.00 [0.00]	6.15 (5.88–6.42) [2.34 (2.23–2.44)]
6/01–12/01	3.23 (2.87–3.58) [1.29 (1.20–1.37)]	1.31(1.16–1.45) [0.46 (0.43–0.49)]	3.53 (3.29–3.78) [3.53 (3.28–3.77)]	0.38 (0.33–0.44) [0.08 (0.07–0.08)]	0.49 (0.43–0.54) [0.19 (0.18–0.21)]	0.00 [0.00]	0.00 [0.00]	8.93 (8.32–9.55) [5.55 (5.16–5.93)]
1/02–4/02	7.92 (6.82–9.01) [3.84 (3.47–4.20)]	1.87 (1.60–2.14) [0.78 (0.71–0.86)]	9.66 (8.74–10.59) [9.56 (8.66–10.47)]	0.00 [0.00]	8.10 (6.97–9.22) [3.92 (3.55–4.29)]	6.34 (5.40–7.27) [2.66 (2.41–2.91)]	0.00 [0.00]	33.88 (30.64–37.13) [20.77 (45.82–53.85)]
5/02–8/02	1.39 (1.21–1.57) [0.58 (0.54–0.63)]	2.46 (2.14–2.79) [0.91 (0.84–0.98)]	31.76 (29.17–34.35) [29.10 (26.84–31.36)]	0.00 [0.00]	9.31 (8.11–10.51) [3.82 (3.52–4.11)]	4.91 (4.25–5.57) [1.74 (1.61–1.88)]	0.00 [0.00]	49.83 (4582–53.85) [36.15 (33.35–38.96)]
9/02–12/02	2.85 (2.49–3.20) [1.06 (0.98–1.114)]	4.97 (4.36–5.57) [1.86 (1.72–2.00)]	34.65 (31.71–37.60) [28.21 (26.07–30.35)]	0.46 (0.39–0.52) [0.12 (0.11–0.13)]	14.67 (12.78–16.57) [5.20 (4.81–5.60)]	12.01 (10.50–13.52) [4.30 (3.97–4.63)]	7.97 (6.73–9.21) [2.00 (1.85–2.15)]	77.57 (71.10–84.04) [42.75 (39.51–45.99)]
1/03–4/03	2.09 (1.78–2.39) [0.89 (0.81–0.98)	0.73 (0.62–0.84) [0.29 (0.26–0.31)]	19.48 (17.24–21.72) [13.33 (12.08–14.58)]	1.40 (1.15–1.65) [0.40 (0.36–0.43)]	7.63 (6.39–8.88) [2.65 (2.41–2.90)]	5.02 (4.18–5.87) [1.52 (1.37–1.66)]	1.50 (1.19–1.80) [0.33 (0.30–0.36)]	37.85 (33.79–41.92) [19.40 (17.58–21.22)]
5/03–8/03	0.00 [0.00]	0.00 [0.00]	4.54 (2.38–6.70) [3.33 (2.10–4.56)]	2.17 (0.43–3.91) [0.57 (0.36–0.78)]	11.52 (1.42–21.61) [2.53 (1.59–3.47)]	0.00 [0.00]	0.00 [0.00]	18.23 (10.43–26.03) [6.43 (4.05–8.81)]

The inapparent to apparent dengue case ratio varied over time (Figure 5). Prior to June 2001, when only DENV-1 and -2 were circulating, the ratio was low (approximately 1∶1), but increased rather dramatically in the last half of 2001, when DENV-3 was most likely introduced. In 2002, during the height of transmission, ratios ranged from 2.6–5.3∶1.

### Geographical Patterns

**Figure 6:**
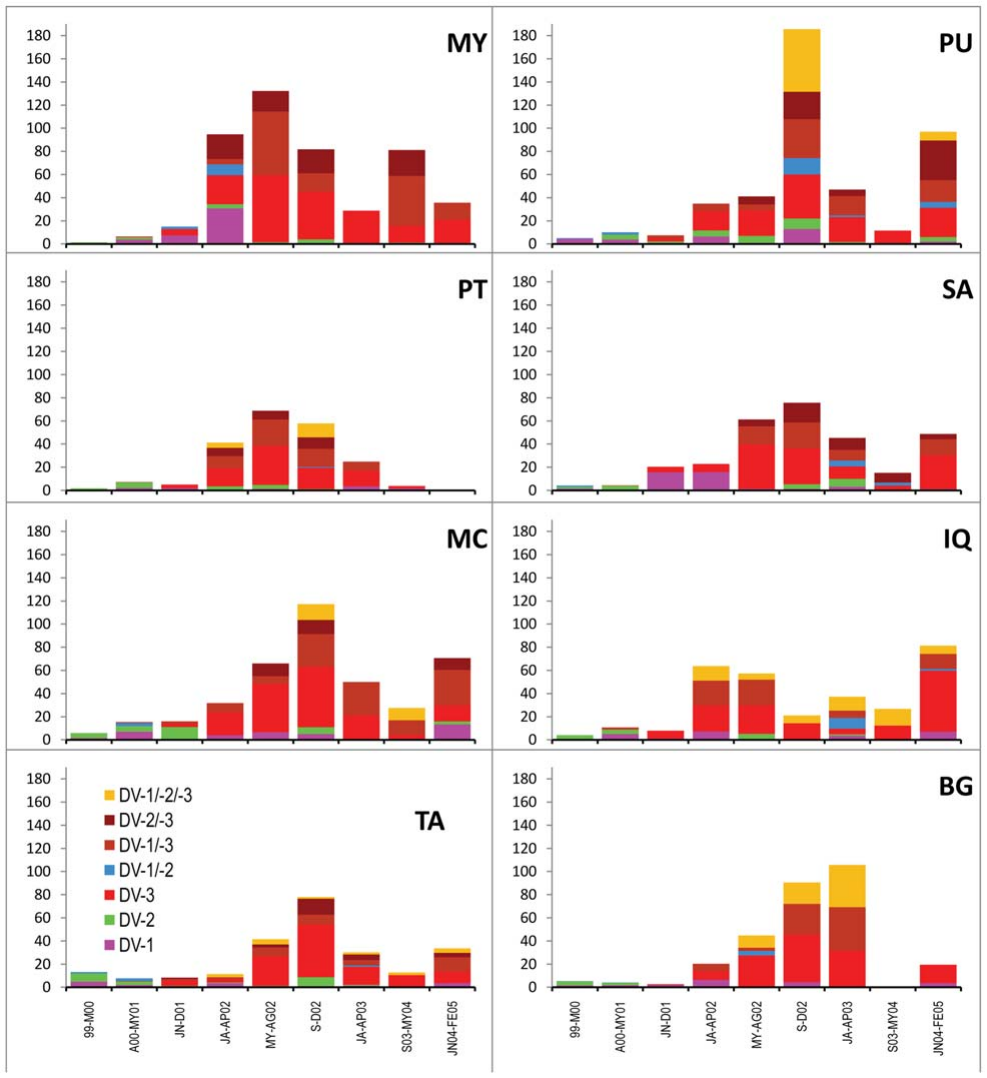
Seroincidence rates from January 1999–February 2005 in 8 geographic zones in Iquitos, Peru. Rates are expressed in 100 person-years at risk with the overall rate for the entire study period shown in the upper left portion of each figure and the zones in the upper right. Rates were calculated for same time periods shown in Table 7.

Rates of DENV seroconversion varied significantly among geographical zones of the city (Figure 6). When calculated for the entire study period seroconversions (SCs) were highest in the two northeastern zones (MY and PU) and one central zone (MC) (mean 28.6–31.5 SCs per 100 p-years at risk), followed by another central zone of IQ (24.5 SCs per 100 p-years at risk). Based on baseline seroprevalence rates, SA had less activity (20.5 SCs per 100 p-years at risk) and TA had more DENV activity than expected (20.4 SC per100 p-years at risk). DENV infection rates were lowest in BG and PT. Temporal patterns of transmission varied by zone. For example, for the period from January 1999 through March 2000 SC rates were similar between DENV-1 and -2, with more activity in TA (13.2 SCs per100 p-years) than in the other zones (1.5–5.8 SCs per100 p-years).

The first observed seroconversions to DENV-3 occurred in IQ, MY, and TA (Figure 4), suggesting virus spread rapidly throughout the city. Indeed, early infections of DENV-3 were distributed across all 8 geographic zones, although most cases occurred in MY and IQ during the first trimester of 2002. Transmission lagged in MC and PU, with SC rates peaking during the September–December 2002 trimester. BG lagged even further, with the highest transmission rates observed during the January–April 2003 time period.

## Discussion

Results from our longitudinal cohort reveal details of dengue dynamics during the transition from interepidemic to epidemic transmission that can help inform increasingly effective surveillance and disease prevention programs. Observations reported here set the stage for subsequent publications addressing the quantitative relationship between *Ae. aegypti* population densities and DENV transmission across periods of different forces of infection with the long-term goal of identifying entomological transmission thresholds [31] and examining age-specific heterogeneities in DENV transmission, the impact of vector and vaccine interventions, and heterogeneities (spatial and temporal) in transmission patterns. We begin by reviewing overall patterns of infection followed by a discussion of the consequences of invasion of a locally novel serotype.

In 1999, at the onset of the study, we detected baseline seroprevalence rates across DENV serotypes in Iquitos that ranged from 56% in 5 year olds to 87–94% in subjects ≥14 years old. Among school age children there appeared to have been greater than a 2-fold rise in cumulative infections since 1992 [12]. Our seroprevalence rates are consistent with those observed in SE Asia [21], [32], [33] and Indonesia [22], although it appears that seroprevalence increases more slowly with age in Iquitos. Possible explanations for this observation include: (1) the sequential nature of serotype introduction into Iquitos, (2) relatively low vector abundance that is associated with a relatively low force of infection, and 3) high rates of migration into and out of the city from remote river communities throughout the Amazon Basin where DENV transmission is minimal. In comparison to other parts of Latin America, seroprevalence rates in Iquitos were lower than those reported in Nicaragua [34] but similar (or slightly higher) than Salvador, Brazil [35] and Maracay, Venezuela [24].

Overall 34.5% of our study population seroconverted to at least 1 DENV serotype during the course of the study. Prior to the introduction of DENV-3, 6–8 of every 100 susceptible individuals (2–2.6 per 100 in the study population) per year had a DENV infection. This is consistent with observations in Kamphaeng Phet, Thailand [36], where transmission of all 4 DENV serotypes was recorded over 3 years with marked spatial and temporal variation [37]. It is also similar to published rates from Nicaragua [34] where only two serotypes were circulating and seroprevalence rates for existing serotypes were extremely high. In both the Iquitos and Managua situations, transmission was observed despite high prevalence of antibodies against multiple serotypes of DENV.

With the virgin-soil epidemic of DENV-3, seroconversion rates increased up to 90 per 100 p-years. School surveillance indicated infection rates increased each trimester of 2002 to a maximum during September–December. DENV-3 seroconversion rates during 2002 were consistent with those previously reported from Iquitos during outbreaks of DENV-1 in 1992–1993 (55%) and DENV-2 in 1993–1995 (86%; [7], [12]). Rates this high have also been reported from Brazil (annual incidence of 70.6%; [35]) and during an outbreak in Bangkok, Thailand (87%; [32]).

The introduction of DENV-3 into Iquitos can be divided into 3 distinct periods: (1) amplification, characterized by serological evidence of infection with the novel serotype and a paucity of clinically apparent cases detected, (2) replacement, characterized by the appearance of the first confirmed clinical cases and a rise in activity of previously circulating serotypes (DENV-1 in this case) concomitantly with cases of the novel serotype, and (3) epidemic, where the novel serotype dominated and caused an outbreak of clinical disease.

### Amplification

We estimate that DENV-3 was introduced into Iquitos between May–July 2001, and then transmission was amplified during the last trimester of that year when unusually high *Ae. aegypti* population densities were observed [17]. We identified 27 participants who had evidence of a DENV infection between May and December 2001 and 2 febrile participants were detected in July 2001. DENV-1 was also circulating at this time and could have been the cause of some of these seroconversions, all of which appeared polytypic. None of these individuals showed evidence of DENV-3 NtAbs prior to this time and subsequent blood samples indicated consistent positive DENV-3 results. Together these cases point to low level serotype 3 activity occurring at least 5–6 month and up to a year prior to our first isolation of DENV-3 during December 2001, despite rigorous virological surveillance. Gubler [38] described a latent period of this duration from introduction to detection of overt disease, but to the best of our knowledge this is the first empirical evidence for this phenomenon.

### Replacement

Virus isolation data from clinics in Iquitos indicated that DENV transmission started to increase in late 2001 and early 2002, but was predominantly DENV-1. By March 2002, DENV-3 virus isolates predominated. In addition there were 2 isolates of the Asian serotype of DENV-2 in early 2002 (Kochel et al. unpublished) and presentation of the first clear clinical cases of DF were observed in our school cohort. The initial increase in DENV-1 transmission supports the idea that entomological and environmental conditions had become favorable for virus transmission. DENV-3 had been present in northwestern Peru since 2000 and Guayaquil, Ecuador since 1999 [13]. There was also virological evidence of DENV-3 circulation in 2001 in Pucallpa and Yarinacocha, both cities located on a major route of river travel and commerce to the south of Iquitos in the Amazon Basin and connected by road to Lima, the capital of Peru [39]. We speculate that the relatively rapid replacement of DENV-1 with DENV-3 and the failure to see continued transmission of Asian DENV-2 can be explained by short term cross protection to heterologous serotypes by recent DENV infections. Nearly the entire Iquitos population was susceptible to DENV-3, whereas seroprevalence rates indicated high rates of previous infection with DENV-1 and DENV-2, and upon introduction of DENV-3 the low percentage of people susceptible to DENV-1 or DENV-2 would be even further reduced by the cross-protective response following DENV-3 infection. A similar pattern was observed recently with the 2008 introduction of DENV-4 into Iquitos [14].

### Epidemic

Infection rates steadily increased throughout 2002 to a peak level of 89 SC per 100 p-years, and rates of clinically apparent disease increased from 13 cases/100 p-years to 19 cases/100 p-years at risk. Transmission decreased dramatically after a city-wide vector intervention based around household ultra low volume adulticide applications and larvicide treatment of *Ae. aegypti*-producing containers (Morrison et al unpublished). In 2004, another outbreak of DF and the first documented cases (7) of DHF were observed. Although no DHF cases were observed in our school surveillance study, reports from local health officials indicated that severe cases of severe illness were observed only during periods of intense transmission at the end of 2002 and again in 2004 (Sihuincha, personal communication) indicating that, in Iquitos, slowing the force of infection with an effective vector control program had beneficial public health consequences.

The spatial evolution of the epidemic illustrated that dengue viruses spread rapidly and moved through different areas of the city at different times. Clinically apparent illness providing the earliest evidence of transmission indicated that infected people were scattered across the city, implicating human movement in the dispersal of virus because flying infected *Ae. aegypti* would not move those distances in that short a time period [40]. Within geographic zones, patterns appeared to reflect the suitability of local conditions for transmission of pathogen. For instance, the timing of peak transmission varied from as early as the January–April 2002 trimester to as late as the same trimester in 2003. In addition, the neighborhood whose peak transmission occurred last in 2003 also had the lowest seroprevalence rates at baseline, which we attribute to consistently lower *Ae. aegypti* indices [17]. Spatial patterns in seroincidence did not follow seroprevalence patterns prior to 2001, but did afterwards. This indicates these areas were of high risk, but herd immunity probably prevented higher transmission rates.

An important observation is the fluctuation in ratio of apparent to inapparent case rates observed over the course of the study. Prior to June 2001 the apparent to inapparent ratio was comparable to that reported by Endy et al. [36] in Thailand, before increasing to 5∶1 during the initiation of the DENV-3 outbreak (Figure 5). In 2003 the ratio increased steadily to 40∶1 for the period between September 2003–May 2004 and 24∶1 during the last half of 2004 when significant clinical disease was observed. This higher ratio of asymptomatic or subclinical cases after May 2003 may in part be due to a decrease in force of infection owing to vector control interventions. After August 2003, additional individuals were recruited into the school surveillance study and the community-based part of the study was discontinued. It is possible that our methodological shift affected the efficiency of our surveillance, but the dramatic shift in incidence of apparent dengue cases is difficult to explain except through positive impact of herd immunity.

Independent of the time interval monitored, infection rates among individuals who had been previously infected with DENV (secondary infections) always exceeded rates of primary infections. The higher incidence of secondary versus primary infections was least dramatic prior to the introduction of DENV-3 and during June 2004–February 2005 after that serotype had been circulating for 2 years. It was during these periods that our observations were consistent with previously published studies [21], [22], [33], [35]. During epidemic transmission in 2002, however, the secondary infection rate exceeded that of primary by 5–8 fold, decreasing in 2003 to 3–5-fold until June 2004 when the rates of primary and secondary infections were nearly equal. This indicates that many people in the population have consistent exposure over time and that seroprevalence patterns can be informative for establishing surveillance zones of consistently low versus high entomological risk. For example, in the geographic zone MY, seroprevalence patterns indicated high rates of historical transmission which also corresponded to some of the highest entomological indices in Iquitos [17]. Although pre-invasion seroincidence rates were relatively low in this zone, post-invasion incidence patterns indicate that transmission increased rapidly here prior to other areas of the city. It is worth noting that the higher incidence rates among participants who had been previously infected by a heterologous serotype, reliance on a serologically naïve population for longitudinal studies would underestimate overall DENV activity and would, thus, be inappropriate for early detection (i.e., sentinels).

A serious impediment to large population-based longitudinal studies on dengue has been logistical and technological barriers associated with serological testing [41]. In our hands, the PRNT, although expensive and labor intensive, provided a reliable way to measure seroconversion rates in a large population. For long term studies the PRNT permits monitoring of cohort participants at longer time intervals (6 months to 1 year) than alternative ELISA or HAI-based assays that require shorter intervals (<3 months) to identify seroconversions. The PRNT provides serotype-specific information, which the other assays do not, that is critical for assessing the risk of populations for epidemic transmission of a novel serotype. PRNT results become more informative as the number of blood samples per participant increases because multiple samples (preferably >3) provide a “serological profile” that can corroborate observations made from a single monitoring interval. For example, when at least one sample was available after the interval where a seroconversion occurred, the infecting serotype could be determined in 75.1% (127/169) of primary and 77.4% (345/446) of secondary infections. For individuals who had no change, 80% of the intervals monitored had consistent PRNT results; 11.7% and 8.3% showed transitory “false” positive and “false” negative results, respectively. Moreover, data from the entire study population can be used to derive correction factors to improve estimates of seroconversions in persons who have no samples taken after the seroconversion interval. We recommend that cutoff values be established empirically using serum samples with known infection histories. Responses may vary based on the local history of DENV serotype circulation and the genetic background of the resident populations [8], [24]. Four serial dilutions rather than the two used in this study improves interpretation of the portion of the human population with ambiguous responses [24]. A challenge to the application of PRNT results in a population-based study is that there remain important knowledge gaps in expected NtAb responses to virus challenges with homologous serotypes and related *Flavivirus* species, cross-reaction patterns among the four DENV serotypes, and how long NtAb titers remain above cutoff values for what proportion of the population.

Our results show a strong similarity between baseline seroprevalence patterns and seroincidence patterns the year following the introduction of a novel dengue serotype. Herd immunity clearly has a profound impact on seroincidence patterns under periods of endemic transmission. In locations like Iquitos, where there was not hyperendemic transmission of all 4 serotypes, surveillance and control programs must consider separate strategies for (1) managing transmission of existing serotypes and (2) preparing for and dealing with either the introduction of novel serotype/genotype or analogous situations where immunity in the human population is low. The major challenge remains early detection of and effective response to novel virus activity. Our experience in Iquitos indicates that establishment of a new serotype requires a short period where environmental conditions are favorable for amplification of virus (high adult *Ae. aegypti* populations and ambient temperatures) and that these characteristics can be incorporated into early warning systems. The lag time from introduction to epidemic transmission that we estimated for DENV-3 in Iquitos (6–12 months) and knowledge of spatially explicit areas of elevated risk (Maynas) should be considered for targeting more effective application of limited resources for dengue prevention.

## Supporting Information

Alternative Language Text S1 Spanish translation of the article by ACM, CR, and Gabriela Vasquez La Torre.
(5.83 MB PDF)

Checklist S1 STROBE checklist.
(0.09 MB DOC)

Table S1 Enrollment and termination dates for 4,586 participants in Longitudinal Cohort Study, carried out from January 1999 to February 2005. Study consisted of longitudinal component ending August 2003 and Active Surveillance component carried out from June 2000 through December 2005.
(0.07 MB DOC)

Table S2 Summary serological profiles where a non-specific broadly cross-reactive antibody response was observed for at least one monitoring interval before the infecting serotype could be identified and possible seroconversions excluded from incidence calculations. In addition, table shows 62 possible seroconversions that were excluded from our incidence calculations.
(0.08 MB DOC)

Table S3 Serotype- specific DENV incidence between February 1999–February 2005 calculated assuming that infections occurred at the midpoint of a sampling interval. The 62 seroconversions classified as probable were included in the rate calculations. Bold rows include school-based component only.
(0.05 MB DOC)

Table S4 Serotype-specific DENV incidence between February 1999 and February 2005 calculated under the assumptions that infection occurred on the final date of a sampling interval. The 62 seroconversions classified as probable were excluded from the rate calculation. Bold rows include school-based component only.
(0.05 MB DOC)

Table S5 Serotype-specific DENV incidence between February 1999 and February 2005 calculated under the assumptions that infection occurred on the final date of a sampling interval. The 62 seroconversions classified as probable were included in the rate calculation. Bold rows include school-based component only.
(0.05 MB DOC)

Table S6 Enrollment and termination dates for 1,846 participants in Active Surveillance School Cohort Sub-Study, carried out from June 2000 to February 2005.
(0.07 MB DOC)
