# Supplementary material for: Epidemiology of Dengue Virus in Iquitos, Peru 1999 to 2005: Interepidemic and Epidemic Patterns of Transmission
Source: PLoS Negl Trop Dis. 2010 May 4;4(5):e670. doi: 10.1371/journal.pntd.0000670 (PMC2864256; doi:10.1371/journal.pntd.0000670)
Supplement: Alternative Language Text S1 — Spanish translation of the article by ACM, CR, and Gabriela Vasquez La Torre. (5.83 MB PDF) [file pntd.0000670.s001.pdf]

Artículo de Investigación
Cabeza: Transmisión de Dengue en Iquitos.

Autor correspondiente:
Amy C. Morrison, Ph.D.
Directora
Laboratorio NMRCD Iquitos
Clínica Naval
Av. La Marina c/Calle Trujillo
Punchana, Perú
Teléfono: 51-65-601470 x107
Fax: 51-65-601472
Departamento de Entomología
Universidad de California
Davis, CA 95616
Teléfono: 530-752-0565
Fax: 530-752-1537
Correo electrónico:
amy.aegypti@gmail.com

Epidemiología del virus de dengue en Iquitos, Perú entre 1999 a 2005: Patrones Inter-epidémicos y epidémicos de Transmisión

Amy C. Morrison[1,2], Sharon L. Minnick[1], Claudio Rocha[2], Brett M. Forshey[2], Steven T. Stoddard[1], Arthur Getis[3], Dana A. Focks[4], Kevin L. Russell[2], James G. Olson[2], Patrick J. Blair[2], Douglas M.Watts[2,#], Moisés Sihuincha[5^], Thomas W. Scott[1] and Tadeusz J. Kochel[2]

[1]Departmento de Entomología, Universidad de California, Davis. One Shields Ave., Davis, CA 95616. [2]Instituto de Investigación de Enfermedades Tropicales de la Marina de los Estados Unidos, 3230 Lima Pl., Washington DC 20521-3230. [3]Departamento de Geografía, Universidad del Estado de San Diego, 5500 Campanile Dr., San Diego, CA, 92182. [4]Infectious Disease Analysis, Gainesville, FL. [5]Laboratorio Referencial de Loreto, Dirección Regional de Salud de Loreto, 332 García Saenz, Iquitos, Perú.

[#]Dirección actual: Office of Research and Sponsored Projects, University of Texas at El Paso, El Paso, TX 79968-0519
[^]Dirección actual: Hospital Apoyo, Departamento de Medicina Interna, Iquitos, Perú.

RESUMEN

Antecedentes: Para desarrollar programas de prevención adaptables al nivel local o aprobar en forma apropiada nuevas vacunas contra el dengue, hay una necesidad urgente por estudios de campo longitudinales y exhaustivos que monitoreen la enfermedad en su población humana así como también la densidad del vector.

Metodología y Hallazgos Principales: Reportamos los resultados provenientes de este tipo de estudio con duración de 5 años realizado en la ciudad Amazónica de Iquitos, Perú donde se monitoreó serológicamente la infección con el virus de dengue (DENV) dentro de una cohorte de 2.400 personas en sus vecindarios así como también casos febriles activos en un subgrupo del cohorte mediante vigilancia de ausencias en sus colegios. En las muestras basales, el 80% de la población del estudio tuvo anticuerpos contra el DENV, la seroprevalencia incrementó con la edad, lo cual demostraba una variación geográfica significativa, con tasas de seroprevalencia por zonas ajustadas por la edad entre 67.1 y 89.9%. Durante los primeros 15 meses del estudio cuando DENV-1 y DENV-2 estaban circulando, las tasas de incidencia para toda la población fueron entre 2 a 3 infecciones/100 años-persona. La introducción de DENV-3 en la segunda mitad de 2001 se caracterizó por 3 periodos distintos: amplificación del virus por un mínimo de 5-6 meses, reemplazo del serotipo previamente circulando, y transmisión epidémica cuando la tasa de incidencia llegó a su pico de 89 infecciones/100 años-persona.

Conclusiones/Significancia: Los patrones de incidencia fueron distintos geográficamente de las tasas de prevalencia basales antes de la introducción del serotipo DENV-3, pero muy similares durante el periodo de invasión. La transmisión varió geográficamente, con su pico de incidencia ocurridos en tiempos diferentes entre las 8 zonas geográficas; cubriendo ~16 km^2 de la ciudad. El tiempo entre la introducción de un nuevo serotipo hasta la transmisión epidémica y el conocimiento de aquellas zonas geográficas con riesgo de transmisión elevada, deben ser consideradas en la aplicación efectiva de los recursos limitados para prevenir el dengue.

INTRODUCCION

Los virus del dengue (DENV) son patógenos importantes re-emergentes que han aumentado su rango geográfico de sólo 9 países hace 60 años a más de 100 hoy en día. En todo el mundo, los estimados indican que 2.5-3.0 mil millones de personas están en riesgo de infección con el virus del dengue, con 50 a100 millones de casos de fiebre de dengue (FD) y entre 250,000 y 500,000 casos de la forma más severa, dengue hemorrágico (FDH) o el síndrome de shock de dengue (SSD) cada año. La incidencia de la enfermedad severa ha ido incrementándose en forma consistente desde los 1950's [1,2,3,4,5].

En la actualidad DENV existe con cuatro virus estrechamente relacionados pero antigénicamente diferentes mediante una cadena simple de ARN (DENV-1, DENV-2, DENV-3 y DENV-4) pertenecientes al género *Flavivirus*, familia *Flaviridae*. La inmunidad inducida por la infección con un serotipo protege contra el mismo y confiere una protección cruzada de forma efímera contra otros serotipos, por lo tanto, las infecciones secuenciales con distintos serotipos son posibles. La etiología de la enfermedad grave no está entendida completamente, pero infecciones secundarias y/o variación de la virulencia del virus han sido implicados con frecuencia. [1,4,5,6,7,8].

Sin una vacuna, la prevención de dengue requiere vigilancia virológica y control vectorial. La evidencia acumulada indica que incorporando la variación en cuanto a la ecología y epidemiología del dengue, es importante para desarrollar programas de prevención más efectivos y adaptables al nivel local. [9,10]. Dichos programas, y futuros ensayos de vacunas en fase III, requieren de un mayor entendimiento de la dinámica de transmisión específica a cada región. Esta información es mejor obtenida de estudios de campo longitudinales y exhaustivos diseñados a proporcionar un entendimiento detallado de los procesos fundamentales de la transmisión del virus, la epidemiología y el control de la enfermedad [9].

En América Latina, después de las campañas de control exitosas que terminaron en los 1960's, hubo una reinvación del vector mosquito *Aedes aegypti* seguido por la emergencia de la enfermedad de dengue como un problema de salud pública prominente, a través de todo el continente. El proceso de urbanización extenso contribuye a la dispersión del vector creando condiciones que aumentan la transmisión de DENV. Éste, está ejemplificado por numerosos brotes dramáticos regionales recientes. Aquí reportamos resultados de estudios longitudinales en Iquitos, Perú, una ciudad Amazónica con una historia de transmisión del virus de dengue extensivamente documentada por el Centro de Investigaciones Tropicales de la Marina de los Estados Unidos (siglas en inglés, NMRCD) desde los primeros años de la década de 1990, cuando presumimos que DENV fue reintroducido en Perú [11]. Iquitos tuvo epidemias de la enfermedad febril causadas por invasiones secuenciales del: DENV-1 en 1990—1991, una cepa Americana de DENV-2 en 1995 [7,12], DENV-3 en 2001 [13], una cepa Asiática de DENV-2 en 2002, y DENV-4 en 2008 [14].

La meta a largo plazo de nuestras investigaciones en Iquitos es adquirir un mejor entendimiento detallado de la dinámica de transmisión de DENV y su relación a los parámetros entomológicos que informarán a los programas de control de vectores y mejorarán la prevención de la enfermedad de dengue. El método que empleamos fue el de monitorear la transmisión del virus y la densidad de las poblaciones de *Ae. aegypti* simultáneamente en las casas y vecindarios de los participantes, de un cohorte longitudinal que representa el 20% de las áreas más pobladas de la ciudad. La abundancia y los patrones de producción de *Aedes aegypti* fueron descritos previamente [15,16,17,18]. En el presente artículo, examinamos los patrones espaciales y temporales de la dinámica de transmisión por los 3 serotipos de DENV antes y durante la invasión de un serotipo nuevo en la zona. Nuestros resultados se basan en un estudio de cohorte prospectivo conducido entre 1999-2005, durante este tiempo observamos una transmisión activa

de los serotipos DENV-1 y DENV-2 e invasión del serotipo DENV-3, que causó una epidemia de enfermedad febril significativa.

MATERIALES Y METODOS

Declaración de Uso Humano: El protocolo de este estudio fue aprobado por los comités de ética (Institutional Review Boards) de la Universidad de California, Davis (Protocolo 2220210788-4(994054), Instituto Nacional de Salud, y Naval Medical Research Center (Protocolo #NMRCD.2001.0008 [DoD 31574]) cumpliendo con todas las regulaciones federales gobernando la protección de sujetos humanos.

Localización de Estudio. Nuestro estudio fue realizado en Iquitos, en una comunidad urbana localizada en la Cuenca Amazónica al Noreste de Perú (73.2°W, 3.7°S, con una elevación de 120 msnm) en el Departamento de Loreto. Los Ríos Amazonas, Nanay e Itaya rodean la ciudad en 3 lados. La población de la ciudad ha crecido desde su último censo publicado con 350,000 habitantes. [19]. Las industrias más comunes son pequeños comerciantes, la pesca, el petróleo, la madera, el turismo y la agricultura. El clima es tropical, con una temperatura promedio diaria de 25.8ºC (promedio mínimo 21.9ºC y máxima 32.4ºC) y una precipitación promedio anual de 3.4 metros (rango 2.7—4.4 metros) durante nuestro estudio. Las precipitaciones ocurren a lo largo del año, en aproximadamente la mitad de los días (51.6%). Iquitos es descrito detalladamente en reportes previos [7,12,15,16,17,18].

La ciudad de Iquitos comprende 4 distritos: San Juan, Maynas, y Punchana de sur a norte y Belén al este (ver Figura 1 en [17]). El área del estudio fue restringida a ≈ 16 km^2 dentro de los distritos de Maynas, Punchana y áreas pequeñas de Belén y San Juan, donde los dividimos en 8 zonas geográficas (descrito detalladamente en [17]) basados en vecindarios reconocidos y

atendidos por distintos Centros de Salud. Brevemente, las 3 zonas localizadas en el norte de la ciudad -- Punchana (PU), Maynas (MY), y San Antonio (SA) – y las 5 zonas al sur de la ciudad -- Putumayo (PT), Iquitos (IQ), Morona Cocha (MC), Bagazán (BG) y Túpac Amaru (TA) – pertenecen predominantemente al distrito de Punchana y Maynas, respectivamente; cada uno tiene su propio gobierno y servicios. Las zonas de Túpac Amaru (TA), Bagazán (BG), y Putumayo (PT) cuentan con áreas donde las casas se inundan estacionalmente.

Diseño de Estudio. Se monitoreó un cohorte de ~2,400 participantes en forma longitudinal desde Enero de 1999 hasta Agosto de 2003 por intervalos de ~ 6 meses para detectar evidencia serológica de infección con DENV. Adicionalmente, el monitoreo serológico continuó hasta Febrero de 2005 en un subgrupo de participantes de edad escolar, quienes también fueron monitoreados por enfermedad activa de dengue basado en su asistencia al colegio (ver la sección abajo **vigilancia febril**). Para obtener una muestra de los participantes geográfica y temporalmente estratificada, cada una de las 8 zonas fue sub-divida en una red de 5 áreas aproximadamente iguales. Tres manzanas de cada una de éstas 5 áreas fueron seleccionadas al azar, dando un total de 15 manzanas de muestras en cada una de las 8 zonas. Enfocamos el reclutamiento en niños de edad escolar (entre 5 a 20 años). Para obtener una muestra más representativa de la población en general, ofrecimos participación a otros miembros de la familia viviendo en la misma casa después que los participantes de edad escolar fueron inscritos. Si los residentes estaban dispuestos a participar, las fichas de consentimiento y asentimiento fueron firmados antes de obtener las muestras de sangre. Se obtuvo consentimientos informados escritos de aquellos participantes mayores de 17 años, y de los padres de los participantes menores de 18 años. Además, obtuvimos asentimiento de participantes entre las edades de 8-17 años. Si los participantes no podían leer o firmar las fichas de consentimiento, obteníamos consentimiento oral en la presencia de un testigo documentado con una firma. Cincuenta

participantes de edad escolar y 10 miembros de sus familias mayores de 20 años fueron reclutados de cada zona en cada mes, repitiendo el proceso cada mes hasta complementar un cohorte base de 2,400 personas (S1; ver [20] Figura 1).

Después de establecer el cohorte basal, visitas de seguimiento por cada participante fueron llevadas a cabo en intervalos de aproximadamente 6 meses. Se consideró participantes perdidos, si no fueron ubicados después de un año desde su última muestra de sangre, a pesar de intentos repetitivos por localizar al participante, o si existía un razón válida para salir del estudio (manifestación del participante de dejar el proyecto, cambio de casa fuera del área de estudio, o fallecimiento). Inscribimos nuevos participantes para reemplazar las pérdidas, con una preferencia por personas de la misma zona geográfica, con el fin de mantener el cohorte activo de ~2,400 individuos. En algunos casos, algunos participantes reingresaron al cohorte después de un viaje largo o deseo de participar en el estudio de nuevo.

En Junio de 2000, un subgrupo de 1,100 miembros del cohorte (edades 5—20) quienes asistían a clases en las mañanas en una de las 29 escuelas públicas fueron reclutados para otro componente del estudio, vigilancia activa de la enfermedad febril [20]. Además de participar en todos los componentes del estudio longitudinal (muestras de sangre cada 6 meses, y encuestas entomológicas) monitoreamos ésta sub-población para casos sintomáticos de infección con DENV. Los flebotomistas visitaron los colegios diariamente, monitorearon la asistencia del niño, y visitaron las casas de los participantes ausentes. Si la razón de la ausencia era una enfermedad febril, obteníamos muestras de sangre agudas y convalescientes y un examen físico era realizado cada día de fiebre. Durante el periodo de vacaciones, éstos niños fueron visitados cada semana. A su vez, enrolamos nuevos participantes para reemplazar a aquellos que abadonaron la escuela por algún motivo, se graduaron o cambiaron de colegio. Después de finalizar la vigilancia entomológica en Agosto de 2003, el estudio de vigilancia activa de los participantes que permanecieron en los colegios, continuaron siendo monitorizados. Al inicio

del año escolar de 2004, reclutamos nuevos participantes de las familias inscritas previamente (hermanos u otros familiares) o casas localizadas en las mismas manzanas de participantes existentes en otro estudio de cohorte basado en la comunidad [20]. El monitoreo de ausencias finalizó en Diciembre de 2004, pero obtuvimos las últimas muestras de sangre de los niños hasta Marzo de 2005. Los aspectos clínicos del estudio serán reportados en publicaciones posteriores.

Recolección de Muestras Sanguíneas. Se obtuvo muestras sanguíneas por venipunción o el método del pinchazo del dedo, aplicando técnicas asépticas estándares, usando un tubo al vacío (Vacutainer®, tapa roja) de 3 ml sin anticoagulante por cada participante, o tres tubos capilares estériles de capacidad de 0.25 ml, que fueron colocados en viales de plástico de tapa rosca. Todas las muestras sanguíneas fueron recolectadas en las casas de los participantes, rotuladas, y almacenadas en coolers portátiles pequeños hasta ser transportados al laboratorio de campo dentro de 4 horas. Las muestras sanguíneas fueron centrifugadas por 10 minutos a 3,000 rpm a 4^{0}C, y el suero transferido a viales de propileno y almacenados a -70^{0}C, has ser transportados en hielo seco al laboratorio de NMRCD en Lima, para ensayos serológicos por anticuerpos contra DENV.

La Prueba de Neutralización por Reducción de Placas Estándar (PRNT). Todas las muestras sanguíneas colectadas del componente longitudinal del estudio fueron analizados por PRNT utilizando una reducción de 70% por los cortes ($PRNT_{70}$). $PRNT_{70}$ fueron llevados a cabo con una dilución final de suero de 1:60 y 1:120 (después de añadir el virus) para DENV-1 y DENV-2, y 1:30 y 1:60 para DENV-3. Para DENV-2, los modelos de regresión lineal fueron ajustados para estimar el porcentaje de reducción en una dilución de corte de 1:80, mientras utilizamos una dilución de corte de 1:60 para DENV-1 y DENV-3. A causa de que el serotipo DENV-3 no fue detectado en Iquitos hasta Diciembre de 2001, no iniciamos los análisis rutinarios para anticuerpos (NtAb) contra el serotipo DENV-3 en sueros colectados hasta Enero

de 2002. Para los participantes que pudieron haberse infectado con el serotipo DENV-3 antes de 2002, se analizaron las muestras previas para el serotipo DENV-3 en forma secuencial hasta que observamos muestras negativas para DENV-3. La circulación del serotipo DENV-4 no fue detectado en Iquitos entre 1993 hasta la fecha final de este estudio de cohorte [14]. Es por esta razón que no hemos analizado las muestras para anticuerpos contra el serotipo DENV-4.

Se siguieron protocolos de PRNT descritos por Sangkawibha [21] y Graham [22], con una modificación “semi-micro” con células (clone 15) de riñones de hámster neonatal (BHK-21) en placas de 12 o 24 pocillos [23]. Las modificaciones son descritas detalladamente en Kochel et al. [8] y Comach et al. [24]. Brevemente, en 0.2 ml de suero de prueba diluido, se mezcla con 0.2ml de medio diluido (Earle’s minimal essential medium [E-MEM], 2% suero bovino fetal [FBS], y antibiótico/antimicótico) conteniendo de 40 a 80 PFU de virus de ensayo y luego incubadas a 4^0 C por 15 horas. A menos que no haya sido mencionado en el texto, las cepas de DENV utilizados en el ensayo de PRNT fueron dos virus aislados en Tailandia en 1974 de casos de FDH [25], DENV-1 16007 y DENV-2 16681, y también una cepa Peruana del año 2001 (de un caso de dengue clásico) de DENV-3, IQD1728. Antes de usar el virus en el ensayo, se amplificaron en cultivo de células C6/36 de *Ae. albopictus* y las alícuotas congeladas a -70°C, para asegurar la consistencia del ensayo a lo largo del estudio. El suero fue inactivado con calor a 56°C por 30 minutos antes de realizar el PRNT. Por triplicado, una mezcla (DENV-1: 16007; DENV-2: 16681; DENV-3: IQT1728) de 0.1 ml de suero de virus fue agregada a un medio de 0.5 ml conteniendo células 1.5 X 10^5 BHK21 y añadidas a un pozo de 24 placas de cultivo de tejido e incubadas a 37^0 C con 5% CO_2 por 3 hrs. Las células fueron entonces cubiertas con 0.5 ml de medio de cubierta (0.6% celulosa de carboximetil, MEM w/o Rojo de fenol, 10% FBS, 0.075% $NaHCO_3$ y antibiótico/antimicótico) e incubadas a 37^0 C con 5% CO_2 por 5 días. El medio fue retirado, y las células lavadas con H_2O y coloreadas con 0.5 ml/pozo de una solución de tinción (0.1% (w/v) Azul de Naftofenol, 1.36% (w/v) Acetato de Sodio, y 6% (v/v) Ácido

Acético Glacial) por 30 min. El colorante fue retirado y las placas contadas. Los resultados fueron expresados como la dilución del suero, determinándose por análisis de regresión, que redujeron el número de placas en 70% comparado con el suero humano normal en la misma dilución. Se incluyó sueros de controles positivos y negativos con cada grupo de sueros analizados. Los valores de corte fueron calculados basados en la habilidad del análisis de maximizar la sensibilidad y especificidad; por cada serotipo fue de 1:60 para DENV-1 y DENV-3, y 1:80 para DENV-2 (Minnick SL, datos no publicados).

Interpretación de los Resultados de PRNT. PRNT es el bioensayo serológico más específico para infecciones de dengue, pero una cantidad de factores pueden influir su sensibilidad y especificidad, incluyendo reacciones cruzadas entre anticuerpos de diferentes serotipos de dengue [8,26,27] (Minnick et al. datos no publicados). Para considerar errores potenciales en la interpretación de los resultados de PRNT para una muestra sanguínea única, determinamos seroconversiones (SC) en el contexto del perfil serológico derivado de los resultados completos de todas las muestras sanguíneas de un participante. Utilizamos los siguientes criterios para identificar seroconversiones por cada serotipo. Se clasificó como una seroconversión, cuando el aumento en el porcentaje de reducción entre una muestra negativa con la próxima muestra de un participante es mayor de 20% y donde los resultados de muestras posteriores se mantuvieron positivas (por ejem., ---+++). Cuando los resultados posteriores de PRNT no eran consistentes, no fueron clasificados como seroconversión. Los consideramos como resultados falsos positivos (FP; por ejem., --+--). Reversiones aisladas fueron consideradas como resultados falsos negativos (FN; por ejem., --++-++). Para estimar tasas de incidencia utilizamos tasas de resultados falsos positivos/falsos negativos para calcular factores de corrección para estimar el número verdadero de seroconversiones entre los participantes, quienes mostraban resultados que indicaban una seroconversión en sus muestras finales (por ejem, ---+). Los factores de corrección fueron calculados como el número de resultados

positivos verdaderos (PV) divido por el número total de seroconversiones aparentes PV/ (PV + PF).

Vigilancia Febril. Cuando observamos un participante con una enfermedad febril durante la vigilancia en los colegios, colectamos muestras de sangre agudas y convalescientes. Sueros de la fase aguda fueron analizados para la infección con DENV, utilizando el aislamiento del virus en cultivos de células o detección de ARN viral por la transcriptasa reversa en la reacción de la cadena de la polimerasa (RT-PCR). Las muestras agudas y convalescientes fueron analizadas para anticuerpos contra el DENV de IgM utilizando la técnica de captura de IgM de ensayo por inmunoabsorción ligado a enzimas (ELISA). Además, un subgrupo de las muestras agudas y convalescientes (colectadas antes de 2002 y un subgrupo de ellas colectadas durante 2004) fueron analizados mediante la prueba de ELISA para anticuerpos de IgG contra el DENV. Se clasificaron episodios febriles como infección de DENV por aislamiento de virus, RT-PCR, serología de IgM (títulos de anticuerpos de IgM elevados [≥1:100] en la muestra aguda, convalesciente o ambos), o serología de anticuerpos de IgG (aumento de hasta 4 veces en los títulos entre las muestras agudas y convalascientes). Las infecciones identificadas por la detección viral, seroconversiones de IgM o elevados títulos de IgM $\geq$ 1:400 fueron contadas como seroconversiones en todos los cálculos de incidencia, aún si los datos de muestras ensayadas por PRNT antes y/o después de las muestras febriles no estuvieron disponibles o discordantes, mientras que los individuos con elevados títulos de IgM 1:100 o serología de IgG, fueron sólo incluidos en los cálculos de incidencia, sólo si los datos de PRNT confirmatorios estuvieron disponibles.

ELISA, Captura de IgM. Se determinaron los títulos de anticuerpos de IgM específicos para dengue utilizando un bioensayo de captura de IgM de ELISA adaptados de protocolos previamente publicados [28]. Brevemente, las placas (formato de 96 pocillos) fueron cubiertas

con anticuerpos anti IgM humana para capturar anticuerpos IgM de los participantes. El IgM específico para del virus fue detectado por la adición de antígeno viral del dengue seguido por líquido hiperimmune ascítico específico para el virus y el conjugado de anticuerpo IgG anti-ratón y peroxidasa de rábano (HRP). Después de añadir un substrato colorimétrico, los valores de densidad óptica (DO) fueron determinados a 410nm. Inicialmente todas las muestras de la fase aguda y convalesciente fueron analizadas en una dilución de 1:100. Las muestras con un DO mayor al valor de corte de referencia, calculadas como el promedio de 7 muestras control negativas de anticuerpos más 3 desviaciones estándares eran consideradas positivas para anticuerpos IgM. Las muestras positivas fueron analizadas posteriormente en diluciones de hasta 4 veces para determinar el título final de anticuerpos.

ELISA de IgG. Se determinaron los títulos de anticuerpos de IgG anti-dengue mediante un bioensayo de ELISA de IgG adaptado de Ansari et al. [29]. Las placas (en formato 96 de pocillos) fueron cubiertos con el antígeno de DENV (mezcla de serotipos 1, 2, 3 & 4) producido en cultivos de células Vero lisadas, infectados o no infectados como controles. Se añadieron alícuotas de suero de los participantes diluido (1:100) a dos pocillos cubiertos con antígeno de dengue y 2 pocillos de control. Después de añadir el HRP-conjugado de anticuerpo IgG de ratón anti-humano, los valores de DO fueron registrados a 410 nm. Los valores de DO ajustados fueron calculados restando la DO del pocillo no infectado, cubierto con antígeno menos el pocillo correspondiente cubierto con el antígeno viral. El valor de corte del DO para determinar la positividad para anticuerpos fue calculado como el promedio ajustado del DO más 3 desviaciones estándares de los sueros de control negativo.

RT-PCR. El ARN viral fue extraído de 140 µL de suero utilizando los Mini Kits QIAamp Viral RNA y siguiendo las instrucciones del fabricante (Qiagen Inc., Valencia, CA 91355). Se realizó RT-PCR específico para el virus dengue, siguiendo el protocolo de Lanciotti et al.[30] en muestras de suero para detección del ARN viral de dengue.

Aislamiento Viral. Se intentó aislar el virus del dengue de todas las muestras de suero agudas. Usando técnicas virológicas estándares, se inocularon en cultivos celulares (células C6/36 y Vero) con los sueros y se monitorearon los efectos citopáticos producidos. Todas las células inoculadas para el aislamiento viral fueron analizadas mediante el ensayo de inmunofluorescencia con anticuerpos monoclonales anti-dengue, específicos para cada serotipo del virus dengue, se hayan mostrando o no efectos citopáticos en las células.

Análisis Estadísticos. Comparamos proporciones usando Chi cuadrado, mediante el procedimiento FREQ en SAS (SAS Versión 8, 1999, SAS Instituto Inc., Cary, NC.) con el nivel de significancia estadística considerado con un alpha de 0.05. Calculamos las tasas de seroprevalencia del estatus serológico observado en la primera muestra sanguínea de todos los participantes enrolados hasta Setiembre de 1999, momento en el que se logró un tamaño de cohorte de 2,400 participantes, con el fin de interpretarlo examinando los perfiles serológicos completos (ver Interpretación de resultados de PRNT arriba).

Cálculo de Las Tasas de Incidencia. Con excepción de los casos con síntomas clínicos, no fue posible identificar la fecha de infección entre cada par de muestras tomadas en los intervalos de cada 6 meses. Entonces, calculamos las tasas de seroincidencia con un intervalo de confianza de 95% para los 10 periodos de tiempo, entre Enero de 1999 y Febrero de 2005, asumiendo que la infección ocurrió en la fecha a la mitad del intervalo monitoreado. Por esta razón, la tasa de incidencia por un intervalo de tiempo fue el número total de seroconversiones con las fechas a la mitad del intervalo ocurridas durante el periodo de tiempo (numerador) dividido por la suma de días-persona que cada participante contribuyó durante el periodo de tiempo (denominador). Las tasas de incidencia de serotipos específicos sólo incluyó los días-persona de los participantes susceptibles al mismo serotipo al inicio del intervalo del monitoreo. Los cálculos asumen que las infecciones ocurridas al final del intervalo de monitoreo, son cuantitativa y cualitativamente similares a aquellos calculos que asumen que las infecciones

ocurrieron en la fecha a la mitad del intervalo; sólo observamos un cambio temporal (Tablas S3-S5). Los mapas y la información del SIG fueron manejados usando Arc-View 3.2 (ESRI, Redlands, CA).

RESULTADOS

Un total de 4,586 participantes fueron enrolados en el estudio entre el 21 de Febrero de 1999 y el 12 de Febrero, 2005 con el fin de mantener un cohorte activo de 2,400 participantes. El setenta y cinco por ciento fue inscrito entre Febrero de 1999 y Marzo de 2000 (Figura 1, Tabla S1). De todos los participantes, 683 (14.9%) individuos fueron perdidos después de proporcionar sólo una muestra de sangre basal, mientras que los 3,903 (85.1%) participantes proporcionaron entre 2 y 14 muestras (Figura 1). Cincuenta y dos porciento (n=2,383) y 31.5% (n=1,445) de los participantes proporcionaron $\geq$ 4 y $\geq$ 7 muestras sanguíneas, respectivamente.

La mayoría de los participantes (77.9%; n=3,571) fueron menores de 18 años de edad. Los participantes del género femenino fueron más comunes que los del género masculino, representando el 57.5% (n=2,637) de la población total del estudio. Entre los participantes adultos, la participación femenina (75.0%) fue significativamente mayor que en varones ($P <$ 0.0001). Los participantes del estudio proporcionaron muestras de sangre únicas o múltiples, las que tuvieron una distribución comparable por género (P= 0.7), pero la proporción de participantes adultos entre los perdidos, quienes nos proporcionaron una sóla muestra, fue ligeramente mayor a quienes nos proporcionaron múltiples muestras (P < 0.003, Figura 2). Las edades de los enrolados fueron distribuidos regularmente (77.9% del cohorte) entre la población con excepción del grupo > 60 años de edad, y fue consistente entre los participantes quienes proporcionaron varias muestras y los que salieron del estudio después, dando una sola muestra (P = 0.17, Figura 2). Los participantes fueron reclutados de 36 a 60 manzanas de la ciudad en cada una de las 8 zonas geográficas (Tabla 2). La proporción de participantes que salieron del

estudio después de dar una muestra de sangre, tuvo un rango de 10.6 a 19.9% ($P < 0.0001$) entre las 8 zonas.

La vigilancia escolar fue realizada cada año en un subgrupo de 1,000-1,180 participantes del cohorte cada año (Tabla S5) entre las edades de 5 y 18, con más mujeres (53.4%) que hombres enrolados.

Bioensayo de PRNT. Aunque el intervalo de tiempo entre las muestras sanguíneas fue relativamente larga (~ 6 meses), observamos anticuerpos cruzados en la primera y algunas veces en la segunda muestra de sangre post-conversión, que fueron más específicos en muestras posteriores (por ejem., [N-N-D12-D1-D1], ver Tabla 1 para las abreviaturas de secuencias). La tasa de reacciones cruzadas aparentes fue similar para infecciones primarias (18.8%, 29/154) y secundarias (23.9%, 33/138).

Los factores de corrección para todas las secuencias observadas se muestran en la Tabla 1. De los 22 participantes cuyas muestras basales de PRNT mostraban evidencia de infección monotípica con el DENV-3 seguida por un infección aparente a DENV-1 o DENV-2, ninguno tuvo resultados de PRNT confirmatorios en las muestras posteriores (factor de corrección = 0 en Tabla 1). En general, los perfiles indicando seroconversión fueron confiables para nuevas infecciones con DENV-3 (factores de corrección > 0.90), pero menos confiables para nuevas infecciones de DENV-1 y DENV-2 (factor de corrección < 0.37). Los factores de corrección disminuyeron para aquellos participantes con un estatus de pre-conversión politípica, pero estas seroconversiones se mantuvieron altas en > 50% de los casos para nuevas infecciones de DENV-3. En algunos casos, las muestras sanguíneas de pre-conversión presentaron una reducción de % entre 1 y 10% menor al valor de corte, seguido por un aumento del 20% en la muestra posterior. En otros casos, la serología sugirió infección con DENV-3, pero negativizó nuevamente en muestras posteriores. Según nuestro criterio, ninguno de estos casos será considerado como una seroconversión verdadera, aunque podrían ser verdaderas, pero nuestros bioensayos no fueron

suficientemente sensibles para detectarlo. Los estimados de incidencia con y sin éstas posibles seroconversiones, no tuvieron un efecto significativo en el resultado (ver Tablas S3-S5).

Seroprevalencia. En general, podemos interpretar el estatus serológico basal para el 99.9% de los participantes del estudio. Para todos los participantes inscritos antes de Octubre de 1999 (n = 2,527) sólo el 19.7% (n = 498) no tuvo evidencia serológica de infección de dengue previa. Aproximadamente la mitad de los participantes tuvieron NtAbs de DENV contra DENV-1 y DENV-2 (1,292, 51.1%) en el periodo de muestreo basal; 366 (14.5%) y 368 (14.6%) tuvieron NtAbs monotípicos a DENV-1 y DENV-2, respectivamente. NtAbs de DENV fueron observados en la misma cantidad en participantes queproporcionron sólo una (286 de 350, 81.7%) o varias (1,740 de 2,174, 80.0%) muestras de sangre (P =0.4642).

La prevalencia de NtAbs de DENV fue mayor entre mujeres (1,170 de 1,424, 82.2%) que varones (856 de 1,100, 77.8%; P = 0.0065) y aumentó con la edad. En adultos ($\geq$18 años) la prevalencia fue de 91.2% (423 de 464), mientras que entre los niños (<18) fue de 77.8% (1,603 de 2,060; P < 0.0001). Más de la mitad del grupo más joven (5 años) presentó evidencia de NtAbs de DENV (56.1%) y ésta proporción se incrementó sostenidamente con más del 87% de los participantes $\geq$ 14 años de edad, lo cuál muestra una historia de infección (Figura 3, P <0.0001). Las tasas de seroprevalencia de dengue varían geográficamente (Figura 4). Las tasas ajustadas por edad tuvieron un rango de 67.1 a 89.9% (P < 0.0001) con tasas menores en las zonas (BG, PT, TA) donde algunas casas fueron localizadas en áreas frente al río que se inundaban estacionalmente.

Seroincidencia. De los 3, 903 participantes quienes proporcionaron por lo menos 2 muestras de sangre, 2,542 (65.1%) no tuvieron cambio en el estatus serológico durante el periodo de observación comparado a su serología basal (Tabla 3). Un total de 1,414 seroconversiones de DENV ocurrieron durante el periodo del estudio entre 1,347 participantes (34.5% del cohorte); 1,281 participantes mostraban evidencia de infección con un sólo serotipo, 65 a 2 serotipos, y 1 a

3 serotipos. De éstas 1,414 seroconversiones, 585 (41.4%) ocurrieron en la muestra de sangre final, los cuales no pueden ser determinadas conclusivamente como seroconversiones verdaderas, y 62 (1.6%) fueron ambiguas (ver *PRNT,* arriba) (Tabla 4 y S2). Los perfiles completos de 14 participantes fueron excluidos porque no pudieron ser interpretados. La mayoría de las infecciones fueron secundarias (75.7%) (Tabla 4). Antes de la invasión por DENV-3 la relación de la incidencia de infecciones primarias a secundarias fue menor a 2 veces, pero incrementó a más de 5-veces en el pico de transmisión epidémica (Tabla 5). Llegando al 2004, la tasa de infección con dengue por infecciones primarias y secundarias fue casi igual. DENV-3 fue responsable del 58.4% de las infecciones y probablemente responsable por el18.8% (respuestas cruzadas que incluyen anticuerpos contra DENV-3) de todas las infecciones de DENV observadas (Tabla 4). No pudimos identificar el serotipo exacto de infección en 24.9% de las infecciones primarias (ver Tabla 4; N-D12, N-D13, N-D23, N-D123) y 17.9% de las infecciones secundarias (ver Tabla 4; D3-D123, D2-D123, D1-D123).

Entre los niños febriles detectados por medio del monitoreo activo de ausencias en sus colegios, 73 infecciones confirmadas fueron identificadas; 20 por aislamiento viral, 14 por RT-PCR, y 39 por serología de IgM (Tabla 6). Otros 42 participantes tuvieron síntomas consistentes con FD y evidencia de seroconversión por medio de sus resultados de PRNT durante el intervalo que monitoreamos sus episodios febriles. De éstos, 11 tuvieron un título de anticuerpos de IgM de 1:100 y 3 tuvieron un aumento en el título de anticuerpos IgG anti-DENV, IgG de hasta 4 veces entre las muestras agudas y convalescientes, éstas muestras fueron incluidas en los cálculos de infecciones sintomáticas de dengue. En total, identificamos 11 casos de DENV-1 (7 primarias, 4 monotípico a politípico), 2 casos de DENV-2 (1 primaria, 1 monotípico a politípico), y 74 casos de DENV-3 (22 primarias, 20 monotípico a politípico, 32 DENV-1/-2 a DENV-1/-2/-3). Para 5 infecciones primarias y 23 secundarias el serotipo de la infección no pudo ser identificado.

Patrones Temporales. Durante los primeros 15 meses del estudio, DENV-1 y DENV-2 circularon a niveles bajos de infección, con aproximadamente 2 y 6 infecciones por cada 100 personas por año dentro de la población total y la población susceptible, respectivamente (Tabla 7). Aparentemente, el serotipo DENV-3 fue introducido en Iquitos en algún momento de 2001. El primer aislamiento de DENV-3 fue recuperado de un caso de FD el 7 Diciembre de 2001 [13](TJK, resultados no publicados). Solamente detectamos 2 casos clínicos de DENV-3 en nuestro estudio de vigilancia escolar antes de Enero de 2002. Además, 2 participantes febriles detectados en Julio de 2001 no tuvieron una respuesta de IgM detectable, pero mostraban un aumento de sus títulos de IgG de hasta 4 veces. En ambos casos los resultados de PRNT tuvieron NtAb de DENV-3 en 2002. La examinación de los resultados de PRNT indicaba que posiblemente la transmisión de DENV-3 empezó en un mínimo de 5-6 meses antes del primer aislamiento. Encontramos la primera evidencia de transmisión de DENV-3 en la muestra obtenida de una niña de 5 años donde su última muestra analizada para PRNT, tomada el 27 Mayo de 2001 indicaba una seroconversión a este serotipo, pero no hubo más muestras posteriores para confirmar éstos resultados. Las próximas indicaciones tempranas de transmisión fueron observadas en muestras tomadas el 24 de Julio de un niño de 12 años y el 27 de Julio de una mujer de 20 años. Las muestras sanguíneas previas tomadas al final del año 2000 de éstos participantes fueron negativas para anticuerpos contra DENV-3, indicando que la infección ocurrió en algún momento entre Diciembre de 2000 y finales de Julio de 2001.

Durante el primer trimestre de 2002 observamos un aumento en la transmisión de DENV-1 y DENV-3, con un incremento sostenible en la incidencia de infección con DENV-3 entre12.9 a 29.6 seroconversiones por 100 años-persona en riesgo entre el primer y último trimestre de 2002, correspondiente a un brote de dengue de grandes proporciones en Iquitos. Las tasas de transmisión de DENV-3 disminuyeron sosteniblemente hasta finales de Agosto del 2003 cuando finalizó nuestro estudio basado en la comunidad. Un subgrupo de la cohorte continuó en

vigilancia escolar desde Setiembre de 2003 hasta Febrero de 2005. En este último subgrupo las tasas de seroconversión fueron consistentes con los trimestres de Mayo-Agosto de 2003 hasta Mayo de 2004, pero aumentaron dramáticamente en la última mitad del 2004, lo cuál correspondió a otro brote de dengue.

Cuando calculamos las tasas de seroconversión por el subgrupo del cohorte longitudinal, enrolado en el programa de vigilancia escolar, fueron parecidas a los de la cohorte completa, con excepción del periodo de Mayo a Diciembre de 2002, cuando las tasas de seroconversión parecieron ser aproximadamente 10 SCs por100 años-persona menor en la cohorte escolar después de ajustar los resultados por patrones de susceptibilidad en la población (Tabla 8, Figura 5, línea rosada comparada con la verde). Las tasas de seroconversión basadas en toda la población fueron casi idénticas. La incidencia de casos sintomáticos de FD en la cohorte escolar fue significativamente menor que la tasa de seroconversión en nuestro muestreo de la población, con un rango de 0.6 a 13 casos por 100 años-persona (Figura 5).

La relación de casos de dengue no aparentes a aparentes varia con el tiempo (Figura 5). Antes de Junio de 2001, cuando sólo DENV-1 y -2 estaban circulando, la relación fue baja (aproximadamente 1:1), pero incrementó dramáticamente en la última mitad de 2001, cuando probablemente el DENV-3 fue introducido. En 2002, durante el pico máximo de la transmisión, las relaciones tuvieron un rango de 2.6-5.3:1.

Patrones Geográficos. Las tasas de seroconversión de DENV variaron significativamente entre las zonas geográficas de la ciudad (Figura 6). Cuando calculamos las seroconversiones (SCs) por el periodo completo del estudio, fue mayor en las dos zonas del noreste (MY y PU) y una zona central (MC) (promedio 28.6-31.5 SCs por 100 años-persona en riesgo), seguido por otra zona central IQ (24.5 SCs por 100 años-persona en riesgo). Basado en las tasas de seroprevalencia basales, SA tuvo menor actividad (20.5 SCs por 100 años-persona en riesgo) y TA tuvo más actividad de DENV de lo esperado (20.4 SC por100 años-persona en riesgo). Las

tasas de infección de DENV fueron más bajas en las zonas de BG y PT. Los patrones temporales de transmisión variaron por zona. Por ejemplo, para el periodo desde Enero de 1999 hasta Marzo de 2000 las tasas de SC fueron similares entre DENV-1 y -2, con más actividad en TA (13.2 SCs por100 años-persona) que en otras zonas (1.5-5.8 SCs per100 años-persona).

Las primeras seroconversiones de DENV-3 observadas ocurrieron en IQ, MY, y TA (Figura 4), sugiriendo una dispersión rápida del virus por toda la ciudad. En efecto, las infecciones tempranas de DENV-3 fueron distribuidas en todas las 8 zonas geográficas, aunque la mayoría de los casos ocurrieron en MY e IQ durante el primer trimestre de 2002. La transmisión se retrasó en MC y PU, con las tasas de SC llegando a su pico máximo durante el trimestre de Setiembre-Diciembre de 2002. BG se retrasó más todavía, con las mayores tasas observadas durante el periodo correspondiente de Enero-Abril de 2003.

DISCUSIÓN

Los detalles relevantes de los resultados de nuestra cohorte longitudinal sobre la dinámica de transmisión de dengue durante la transición de un periodo inter-epidémico a un periodo epidémico ayudaría a informar a los programas de vigilancia y prevención de dengue que se encuentran constantemente incrementando su eficacia. Las observaciones reportadas aquí constituyen la base para publicaciones posteriores que tratan la relación cuantitativa entre las densidades poblacionales de *Ae. aegypti* y transmisión de DENV durante periodos con distintas fuerzas de infección y la meta a largo plazo de identificar los niveles umbrales entomológicos de transmisión [31], examinar las heterogeneidades de transmisión de DENV entre grupos etáreos, el impacto de intervenciones vectoriales o de vacuna, y heterogeneidades (espaciales y temporales) en patrones de transmisión. Empezamos con una revisión de los patrones de

infección en general, seguido por una discusión de las consecuencias de una invasión de un serotipo nuevo al área local.

En 1999, al inicio del estudio, detectamos tasas de seroprevalencia basales para todos los serotipos de DENV en Iquitos obteniendo rangos desde 56% en niños de 5 años, hasta 87-94% en sujetos $\geq$ 14 años. Entre los niños de edad escolar, hubo aparentemente un aumento mayor de hasta 2x infecciones acumuladas desde 1992 [12]. Nuestras tasas de seroprevalencia son consistentes con las observadas en el sudeste de Asia [21,32,33] e Indonesia [22], aunque aparentemente la seroprevalencia se incrementa más lento con la edad en Iquitos. Posibles explicaciones para ésta observación incluye: (1) la introducción de los serotipos, en Iquitos, en forma secuencial, (2) la abundancia relativamente baja del vector asociada a su vez con una fuerza de infección también relativamente baja, y 3) las tasas altas de migración dentro y fuera de la ciudad desde comunidades aisladas de los ríos de la cuenca Amazónica donde la transmisión de DENV es mínima. En comparación con otras partes de América Latina, las tasas de seroprevalencia en Iquitos fueron menores que las reportadas en Nicaragua [34] pero similar (o ligeramente mayor) que Salvador, Brasil [35] y Maracay, Venezuela [24].

En general el 34.5% de nuestra población de estudio se infectaron con por lo menos 1 serotipo de DENV durante el curso del estudio. Antes de la introducción de DENV-3, 6--8 de cada 100 individuos susceptibles (2-2.6 por 100 en toda la población del estudio) por año tuvieron una infección con DENV. Esto es consistente con observaciones hechas en Kamphaeng Phet, Tailandia [36], donde la transmisión de todos los 4 serotipos de DENV fueron registradas durante un periodo de 3 años con una variación espacial y temporal marcada [37]. También, las tasas son similares a las tasas publicadas de Nicaragua [34] donde sólo dos serotipos estaban circulando y las tasas de seroprevalencia para éstos serotipos fueron extremadamente altas. En las situaciones de Iquitos y Managua, observamos transmisión aunque hubo altos niveles de prevalencia de anticuerpos contra varios serotipos de DENV.

Con una epidemia de DENV-3 de una zona virgen, las tasas de seroconversión aumentaron a 90 por 100 años-personas. La vigilancia escolar indicó que las tasas de infección se incrementaron cada trimestre del 2002 hasta llegar a un máximo durante el periodo de Setiembre-Diciembre. Las tasas de seroconversión de DENV-3 durante 2002 fueron consistentes con las previamente reportadas de Iquitos durante los brotes de DENV-1 en 1992-1993 (55%) y DENV-2 en 1993-1995 (86%; [7,12]). Estas tasas altas han sido también reportadas en Brasil (incidencia anual de 70.6%; [35]) y durante un brote en Bangkok, Tailandia (87%, [32]).

La introducción de DENV-3 a Iquitos puede ser dividido en 3 periodos distintos: (1) amplificación, caracterizada por evidencia serológica de infección con un serotipo nuevo y una insuficiencia de casos aparentes clínicamente detectados, (2) reemplazo, caracterizado por la aparición de los primeros casos clínicos y un aumento de la actividad de los serotipos previamente circulando (DENV-1 en este caso), concomitantemente con casos del serotipo nuevo, y (3) epidemia, donde el serotipo nuevo fue dominante y causó un brote de la enfermedad clínica.

Amplificación. Estimamos que el serotipo DENV-3 fue introducido a Iquitos entre Mayo y Julio 2001, y después la transmisión se amplificó durante el último trimestre del mismo año cuando las densidades poblacionales de *Aedes aegypti* observadas fueron extraordinariamente altas [17]. Identificamos 27 participantes quienes tuvieron evidencia de infección de DENV entre Mayo y Diciembre de 2001 y 2 participantes febriles fueron detectados en Julio de 2001. En este momento DENV-1 también circulaba, y pudo haber causado algunas de éstas seroconversiones, en donde todos parecían politípicos. Ninguno de estos individuos mostró evidencia de NtAbs de DENV-3 antes de este tiempo y las muestras sanguíneas posteriores indicaban resultados positivos para DENV-3 consistentes. En conjunto estos casos indican la actividad del serotipo 3 a niveles bajos ocurriendo por lo menos 5-6 meses y hasta un año antes de nuestro primer aislamiento de DENV-3 en Diciembre de 2001, a pesar de la vigilancia

virológica rigorosa. Gubler [38] describió un periodo latente de ésta duración desde la introducción hasta la detección de casos clínicos de la enfermedad, pero basados en nuestros conocimientos, ésta es la primera evidencia empírica demostrando este fenómeno.

Reemplazo. Los datos de aislamiento viral de las clínicas de Iquitos indicaron que la transmisión del DENV empezó a incrementarse al final de 2001 y al inicio de 2002, pero el serotipo predominante fue DENV-1. Y en Marzo de 2002, los aislamientos virales de DENV-3 fueron predominantes. Además, hubo 2 aislamientos del DENV-2 Asiático al inicio de 2002 (Kochel et al. datos no publicados) y los primeros casos clínicos de FD fueron observados en nuestra cohorte escolar. El aumento inicial de transmisión de DENV-1 sustenta la idea que condiciones entomológicas y ambientales fueron favorables para la transmisión del virus. DENV-3 estuvo presente al noroeste de Perú desde 2000 y en Guayaquil, Ecuador desde 1999 [13]. También hubo evidencia virológica de circulación de DENV-3 en 2001 en Pucallpa y Yarinacocha, ambas ciudades localizadas en rutas importantes para el transporte fluvial y comercial al sur de Iquitos, en la Cuenca Amazónica y está conectado por carretera a Lima, la capital de Perú [39]. Especulamos, que el reemplazo relativamente rápido del serotipo DENV-1 con DENV-3 y la falta de detectar ésta transmisión de DENV-2 Asiático puede ser explicado por la protección cruzada de corto plazo a anticuerpos de serotipos heterólogos por medio de infecciones recientes con DENV. Casi toda la población de Iquitos era susceptible a DENV-3, mientras que las tasas de seroprevalencia indicaban tasas altas para infección previa con DENV-1 y DENV-2, y después de la introducción de DENV-3 el porcentaje bajo de personas susceptibles a DENV-1 o DENV-2 sería reducido aún más por la respuesta cruzada de proteccón que siguió después dela infección de DENV-3. Un patrón similar fue observado recientemente con la introducción de DENV-4 a Iquitos en 2008 [14].

Epidemia. Las tasas de infección se incrementaron consistentemente durante 2002 a un pico máximo de 89 SC por 100 años-personas, y las tasas de casos de la enfermedad clínicamente

aparentes se incrementaron desde 13 casos/100 años-persona a 19 casos/100 años-persona en riesgo. La transmisión disminuyó dramáticamente después de una intervención vectorial implementada en toda la ciudad utilizando la aplicación de formulaciones de volumen ultra bajo (ULV) de adulticida de casa en casa, y tratamiento de envases que produzcan *Ae. aegypti* con larvicidas (Morrison et al. datos no publicados). En 2004, observamos otro brote de FD y los primeros 7 casos de FHD. Aunque no hubo casos de FHD observado en nuestro estudio de vigilancia escolar, reportes locales oficiales de salud indicaban que hubo varios casos severos observados sólo durante periodos de transmisión intensos a finales de 2002 y de nuevo en 2004 (Sihuincha, comunicación personal) indicando que, en Iquitos, disminuyendo la fuerza de infección con un programa de control vectorial tenía consecuencias beneficiosas para la salud pública.

La evolución espacial de la epidemia ilustró que los DENV se dispersaron rápidamente y pasaron por todas las áreas de la ciudad durante tiempos distintos. Casos clínicamente aparentes proporcionaron la evidencia más temprana de transmisión indicando que personas infectadas se dispersaron por toda la ciudad, implicando a los movimientos humanos en la dispersión del virus, porque los *Ae. aegypti* infectados no se trasladarían volando a éstas distancias durante un periodo de tiempo tan corto [40]. Dentro de las zonas geográficas, aparentemente los patrones reflejaban la conveniencia de las condiciones locales para la transmisión del patógeno. Por ejemplo, al momento que observamos el pico de transmisión, este variaba de lo más temprano en el trimestre de Enero-Abril de 2002 hasta el más tarde en el mismo trimestre pero en 2003. Adicionalmente, el vecindario donde el pico de transmisión ocurrió a finales de 2003 también tuvo tasas de seroprevalencia basales menores, lo cuál atribuimos a los índices Aédicos consistentemente bajos [17]. Los patrones espaciales de seroincidencia no fueron consistentes con los patrones de seroprevalencia antes de 2001, pero si fueron consistentes después. Esto

indica que estas áreas son de alto riesgo, pero la inmunidad poblacional probablemente previno tasas de transmisión más altas.

Una observación importante es la fluctuación, en la relación de casos aparentes y no aparentes observados a lo largo del estudio. Antes de Junio de 2001 la relación de casos aparentes a no aparentes fue comparable a la misma relación reportada por Endy et al. [36] en Tailandia, y se incrementó a 5:1 al inicio de brote DENV-3 (Figura 5). En el 2003 la relación incrementó consistentemente a 40:1 para el periodo entre Setiembre de 2003 y Mayo de 2004 y 24:1 durante la segunda mitad de 2004 cuando observamos niveles significativos de casos clínicos. Ésta relación mayor de casos asintomáticos o subclínicos después de Mayo de 2003 podría ser resultado en parte de una disminución en la fuerza de infección causadas por las intervenciones de control vectorial. Después de Agosto de 2003, individuos adicionales fueron reclutados para el estudio de vigilancia escolar y las actividades realizadas en el estudio basado en la comunidad fueron suspendidas. Es posible que nuestro cambio en la metodología afectara la eficiencia de nuestra vigilancia, pero el cambio dramático de incidencia de casos de DENV aparentes sólo puede ser explicado por el impacto positivo de inmunidad poblacional.

Independientemente del intervalo de tiempo monitoreado, las tasas de infección entre las personas previamente infectadas con DENV (infecciones secundarias) siempre fueron mayores que las tasas para infecciones primarias. La incidencia mayor observada en infecciones secundarias versus infecciones primarias fue menos dramática, antes de la introducción de DENV-3 y entre el intervalo de Junio de 2004 a Febrero de 2005, después que éste serotipo estuvo circulando 2 años. Durante dichos periodos nuestras observaciones fueron consistentes con los de estudios previamente publicados [21,22,33,35]. Sin embargo; durante la transmisión epidémica en 2002, la tasa de infección secundaria excedió la tasa de infección primaria por 5-8x, dismuyendo en 2003 a 3-5x hasta Junio de 2004 cuando las tasas de infección primaria y secundaria fueron casi igual. Esto indica que miembros de la población se encuentran

consistentemente expuestos al virus de dengue temporalmente y que los patrones de seroprevalencia pueden ser informativos para establecer zonas de vigilancia de riesgo entomológico consistentemente bajo y alto. Por ejemplo, en la zona geográfica de MY, los patrones de seroprevalencia indicaban tasas altas de transmisión histórica que también correspondían a las zonas con los índices entomológicos más altos [17]. Aunque las tasas de seroincidencia antes de la invasión fueron relativamente bajos en esta zona, los patrones de incidencia después de la invasión indicaban que la transmisión se incrementó más rápidamente en ésta zona que en otras áreas en la ciudad. Cabe señalar que las tasas de incidencia más altas observadas en los participantes, quienes han sido infectados previamente con un serotipo heterólogo, dependen de una población sin previa exposición al virus y para estudios longitudinales pudiera resultar una subestimación de la actividad de DENV dentro de la ciudad, y por esta razón sería inapropiado para sistemas de detección temprana (por ejem., centinelas).

Un impedimento serio para realizar estudios longitudinales grandes en poblaciones humanas, que estudian dengue, han sido las barreras logísticas y tecnológicas asociadas con los bioensayos serológicos [41]. Aunque el bioensayo PRNT es caro y laborioso, en nuestras manos, nos proporcionó un método confiable para medir las tasas de seroconversión en poblaciones humanas grandes. Para estudios de largo plazo, el PRNT nos permite monitorear a participantes de un cohorte por intervalos de tiempo más largos (6 meses a 1 año) que los alternativos, los bioensayos de ELISA o Inhibición de Hemaglutinación, requieren de intervalos más cortos (< 3 meses) para identificar seroconversiones. El PRNT proporciona información de serotipo específico, algo que los demás bioensayos, no pueden proporcionar, lo cual es crítico para determinar el riesgo de transmisión epidémico de un serotipo nuevo. Los resultados de PRNT se vuelven más informativos cuando el número de muestras sanguíneas por participante aumenta porque son muestras múltiples (preferible > 3) que proporcionan un “perfil serológico”, que puedan corroborar las observaciones detectadas en un sólo intervalo de monitoreo. Por

ejemplo, para individuos, en donde por lo menos una muestra esta disponible después del intervalo donde ocurrió la seroconversión, el serotipo del virus puede ser determinada en un 75.1% (127/169) de infecciones primarias y 77.4% (345/446) de infecciones secundarias. Para individuos sin ningún cambio serológico, 80% de los intervalos monitoreados tuvieron resultados de PRNT consistentes; 11.7% y 8.3% mostraban resultados transitorios de falsos positivos y falsos negativos, respectivamente. Además, los datos de toda la población del estudio pueden ser usados para derivar factores de corrección para mejorar las estimaciones de seroconversiones en personas que no tienen muestras tomadas después del intervalo donde ocurrió la seroconversión. Recomendamos que los valores de corte sean establecidos empíricamente usando muestras de suero con historia de infección conocida. Las respuestas inmunológicas pueden variar basadas en la historia local de circulación del serotipo de DENV y los antecedentes genéticos en las poblaciones humanas [8,24]. Utilizando cuatro diluciones seriales en vez de los dos utilizados en este estudio, mejora la interpretación de los resultados para el sector de la población humana con resultados ambiguos. [24]. Un desafío para la aplicación de resultados del PRNT en estudios de poblaciones humanas, es que todavía existen brechas en el conocimiento sobre los niveles esperados de NtAbs del virus ante infecciones con serotipos homólogos y especies de *Flavivirus* relacionados, con patrones de reacciones cruzadas entre los cuatro serotipos de DENV, y la duración de títulos sobre los valores de corte de NtAb para diferentes sectores de la población humana.

Nuestros resultados muestran paridad significativa entre los patrones de seroprevalencia basales y los patrones de seroincidencia al año después de la introducción de un serotipo nuevo de DENV. Claramente, la inmunidad poblacional tiene un impacto profundo sobre los patrones de seroincidencia durante periodos de transmisión endémica. En localidades como Iquitos, donde no hay transmisión hiperendémica de todos los 4 serotipos, los programas de vigilancia y control tienen que considerar estrategias separadas para (1) el manejo de la transmisión de serotipos

existentes y (2) la preparación y/o reacción de la introducción de un serotipo/genotipo nuevo o una situación análoga donde la inmunidad en la población humana es baja. El mayor desafío es la detección temprana y la reacción efectiva a un virus nuevo. Nuestra experiencia en Iquitos nos indica que para que se establezca un nuevo serotipo en un área, existe la necesidad de un periodo corto de tiempo en el cual las condiciones ambientales son favorables para la amplificación del virus (altas densidades de *Ae. aegypti* adultos y altas temperaturas ambientales) y donde éstas características pueden ser incorporadas en un sistema de alerta temprano. El tiempo de retraso entre la introducción del virus hasta la transmisión epidémica que estimamos para DENV-3 en Iquitos (6-12 meses) y el conocimiento de las áreas espacialmente explícitas de riesgo elevado (Maynas), deben ser considerados para enfocar la aplicación de recursos limitados más eficaces para la prevención del dengue.

Agradecimientos. Agradecemos a los residentes de la ciudad de Iquitos, Perú por permitirnos realizar este estudio dentro y cerca de sus viviendas. Estamos muy agradecidos por el apoyo de la Dirección Regional de Salud incluyendo Drs. Carlos Calampa, Jorge Reyes, Rubén Naupay, Carlos Vidal, Hugo Rodríguez y Martín Casapía, quienes facilitaron nuestro trabajo en Iquitos. Karla Block supervisó el equipo de flebotomistas y proporcionó apoyo logístico por el proyecto. Agradecemos a Renán Ricarte Ruíz Chávez por procesar las muestras sanguíneas y mantener al equipo de flebotomistas felices y Leslye Angulo por la búsqueda de participantes escolares. Las encuestas serológicas fueron realizadas por Joel Cahuachi Tuesta, Jacqueline Cárdenas Pérez, Junnelhy Mireya Flores López, Juan Flores Michi, Marcelina Flores Michi, Elga López Guerrero, Xiomara Mafaldo García, Nora Marín Moreno, María Juana Martínez Salas, Orfelinda Morales Tejada, Geraldine Ocmín Galán, Zenith María Pezo Villacorta, Zoila Martha Reátegui Chota, Luis Riveros López, Rubiela Nerza Rubio Briceño, Rosana Magaly Sotero Jiménez, Zenith Tamani Guerrero, Moisés Tanchiva Tuanama, Sarita Del Pilar Tuesta Dávila.

Agradecemos a Angélica Espinoza, Roxana Caceda, y Roger Castillo por realizar los bioensayos serológicos, Carolina Guevara por su supervisión al Laboratorio de Virología de NMRCD y Juan Pérez por el manejo de datos. Dr. Truman Sharp, Lucy Rubio y Roxana Lescano del U.S. Naval Medical Research Center en Lima, Perú donde su participación fue fundamental para las facilidades del estudio. A su vez también, a Gabriela Vásquez La Torre por su ayuda con la traducción de este artículo a la versión en español.

Aclaración. Las ideas expresadas en este artículo son de los autores y no necesariamente reflejan la política oficial del Departamento de la Marina de Guerra de los Estados Unidos, ni del Departamento de Defensa, y mucho menos del gobierno de los Estados Unidos.

REFERENCIAS

1. Monath TP (1994) Dengue: the risk to developed and developing countries. Proc Natl Acad Sci U S A 91: 2395-2400.
2. Kuno G (1995) Review of the factors modulating dengue transmission. Epidemiol Rev 17: 321-335.
3. Gubler DJ (1997) Dengue and dengue hemorrhagic fever: its history and resurgence as a global public health problem. In: Gubler DJ, Kuno, G., editor. Dengue and dengue hemorrhagic fever. London, UK: CAB International. pp. 1-22.
4. Gubler DJ (2002) Epidemic dengue/dengue hemorrhagic fever as a public health, social and economic problem in the 21st century. Trends Microbiol 10: 100-103.
5. Gubler DJ (2004) The changing epidemiology of yellow fever and dengue, 1900 to 2003: full circle? Comp Immunol Microbiol Infect Dis 27: 319-330.
6. Rothman AL, Ennis FA (1999) Immunopathogenesis of Dengue hemorrhagic fever. Virology 257: 1-6.
7. Watts DM, Porter KR, Putvatana P, Vasquez B, Calampa C, et al. (1999) Failure of secondary infection with American genotype dengue 2 to cause dengue haemorrhagic fever. Lancet 354: 1431-1434.
8. Kochel TJ, Watts DM, Halstead SB, Hayes CG, Espinoza A, et al. (2002) Effect of dengue-1 antibodies on American dengue-2 viral infection and dengue haemorrhagic fever. Lancet 360: 310-312.
9. Morrison AC, Zielinski-Gutierrez E, Scott TW, Rosenberg R (2008) Defining challenges and proposing solutions for control of the virus vector Aedes aegypti. PLoS Med 5: e68.
10. Scott TW, Morrison AC (2010) Vector dynamics and transmission of dengue virus: implications for dengue surveillance and prevention strategies : vector dynamics and dengue prevention. Curr Top Microbiol Immunol 338: 115-128.
11. Phillips I, Need J, Escamilla J, Col n E, S nchez S, et al. (1992) First documented outbreak of dengue in the Peruvian Amazon region. Bulletin of the Pan American Health Organization 26: 201-207.
12. Hayes CG, Phillips IA, Callahan JD, Griebenow WF, Hyams KC, et al. (1996) The epidemiology of dengue virus infection among urban, jungle, and rural populations in the

Amazon region of Peru. The American journal of tropical medicine and hygiene 55: 459-463.

13. Kochel T, Aguilar P, Felices V, Comach G, Cruz C, et al. (2008) Molecular epidemiology of dengue virus type 3 in Northern South America: 2000--2005. Infect Genet Evol 8: 682-688.
14. Forshey BM, Morrison AC, Cruz C, Rocha C, Vilcarromero S, et al. (2009) Dengue virus serotype 4, northeastern Peru, 2008. Emerg Infect Dis 15: 1815-1818.
15. Getis A, Morrison AC, Gray K, Scott TW (2003) Characteristics of the spatial pattern of the dengue vector, Aedes aegypti, in Iquitos, Peru. The American journal of tropical medicine and hygiene 69: 494-505.
16. Morrison AC, Astete H, Chapilliquen F, Ramirez-Prada C, Diaz G, et al. (2004) Evaluation of a sampling methodology for rapid assessment of Aedes aegypti infestation levels in Iquitos, Peru. Journal of medical entomology 41: 502-510.
17. Morrison AC, Gray K, Getis A, Astete H, Sihuincha M, et al. (2004) Temporal and geographic patterns of Aedes aegypti (Diptera: Culicidae) production in Iquitos, Peru. Journal of medical entomology 41: 1123-1142.
18. Schneider JR, Morrison AC, Astete H, Scott TW, Wilson ML (2004) Adult size and distribution of Aedes aegypti (Diptera: Culicidae) associated with larval habitats in Iquitos, Peru. Journal of medical entomology 41: 634-642.
19. INEI (Instituto Nacional de Estadística e Informática) (2008) Censos Nacionales 2007: XI de Población y VI de Vivienda. Lima, Peru.
20. Rocha C, Morrison AC, Forshey BM, Blair PJ, Olson JG, et al. (2009) Comparison of two active surveillance programs for the detection of clinical dengue cases in Iquitos, Peru. Am J Trop Med Hyg 80: 656-660.
21. Sangkawibha N, Rojanasuphot S, Ahandrik S, Viriyapongse S, Jatanasen S, et al. (1984) Risk factors in dengue shock syndrome: a prospective epidemiologic study in Rayong, Thailand. I. The 1980 outbreak. Am J Epidemiol 120: 653-669.
22. Graham RR, Juffrie M, Tan R, Hayes CG, Laksono I, et al. (1999) A prospective seroepidemiologic study on dengue in children four to nine years of age in Yogyakarta, Indonesia I. studies in 1995-1996. Am J Trop Med Hyg 61: 412-419.
23. Morens DM, Halstead SB, Repik PM, Putvatana R, Raybourne N (1985) Simplified plaque reduction neutralization assay for dengue viruses by semimicro methods in BHK-21 cells: comparison of the BHK suspension test with standard plaque reduction neutralization. J Clin Microbiol 22: 250-254.
24. Comach G, Blair PJ, Sierra G, Guzman D, Soler M, et al. (2008) Dengue Virus Infections in a Cohort of Schoolchildren from Maracay, Venezuela: A 2-Year Prospective Study. Vector Borne Zoonotic Dis.
25. Halstead SB, Marchette NJ (2003) Biologic properties of dengue viruses following serial passage in primary dog kidney cells: studies at the University of Hawaii. Am J Trop Med Hyg 69: 5-11.
26. Kuno G (2003) Serodiagnosis of flaviviral infections and vaccinations in humans. Adv Virus Res 61: 3-65.
27. Thomas SJ, Nisalak A, Anderson KB, Libraty DH, Kalayanarooj S, et al. (2009) Dengue plaque reduction neutralization test (PRNT) in primary and secondary dengue virus infections: How alterations in assay conditions impact performance. Am J Trop Med Hyg 81: 825-833.
28. Innis BL, Nisalak A, Nimmannitya S, Kusalerdchariya S, Chongswasdi V, et al. (1989) An enzyme-linked immunosorbent assay to characterize dengue infections where dengue and Japanese encephalitis co-circulate. Am J Trop Med Hyg 40: 418-427.

29. Ansari MZ, Shope RE, Malik S (1993) Evaluation of vero cell lysate antigen for the ELISA of flaviviruses. Journal of clinical laboratory analysis 7: 230-237.
30. Lanciotti RS, Calisher CH, Gubler DJ, Chang GJ, Vorndam AV (1992) Rapid detection and typing of dengue viruses from clinical samples by using reverse transcriptase-polymerase chain reaction. J Clin Microbiol 30: 545-551.
31. Scott TW, Morrison AC (2003) *Aedes aegypti* density and the risk of dengue virus transmission. In: Takken W, Scott TW, editors. Ecological Aspects for Application of Genetically Modified Mosquitoes. Norwell, MA: Kluwer Academic Publishers. pp. 187-206.
32. Burke DS, Nisalak A, Johnson DE, Scott RM (1988) A prospective study of dengue infections in Bangkok. Am J Trop Med Hyg 38: 172-180.
33. Thein S, Aung MM, Shwe TN, Aye M, Zaw A, et al. (1997) Risk factors in dengue shock syndrome. Am J Trop Med Hyg 56: 566-572.
34. Balmaseda A, Hammond SN, Tellez Y, Imhoff L, Rodriguez Y, et al. (2006) High Seroprevalence of Antibodies Against Dengue Virus in a Prospective Study of Schoolchildren in Managua, Nicaragua. Tropical Medicine & International Health 11: 935-942.
35. Teixeira Mda G, Barreto ML, Costa Mda C, Ferreira LD, Vasconcelos PF, et al. (2002) Dynamics of dengue virus circulation: a silent epidemic in a complex urban area. Trop Med Int Health 7: 757-762.
36. Endy TP, Chunsuttiwat S, Nisalak A, Libraty DH, Green S, et al. (2002) Epidemiology of inapparent and symptomatic acute dengue virus infection: a prospective study of primary school children in Kamphaeng Phet, Thailand. American journal of epidemiology 156: 40-51.
37. Endy TP, Nisalak A, Chunsuttiwat S, Libraty DH, Green S, et al. (2002) Spatial and temporal circulation of dengue virus serotypes: a prospective study of primary school children in Kamphaeng Phet, Thailand. American journal of epidemiology 156: 52-59.
38. Gubler D (1992) Dengue. In: Monath TP, editor. The Arboviruses: Epidemiology and Ecology. Boca Raton, FL: CRC Press. pp. 223-260.
39. Cobos Z. M, Gutierrez P. V, Garcia M. M, Mamani Z. E, Fernandez C. R, et al. (2004) Estudio serológico y virológico del brote de dengue en la Provincia de Coronel Portillo: Ucayali, Perú (2000 - 2001). Rev perú med exp salud publica 21: 139-145.
40. Harrington LC, Scott TW, Lerdthusnee K, Coleman RC, Costero A, et al. (2005) Dispersal of the dengue vector Aedes aegypti within and between rural communities. Am J Trop Med Hyg 72: 209-220.
41. Kuno G (1997) Factors influencing the transmission of dengue viruses. In: Gubler DJ, Kuno G, editors. Dengue and Dengue Hemorrhagic Fever. New York: CAB International. pp. 61-88.

Tabla 1. Factores de corrección para estimar el número verdadero de seroconversiones para los participantes quienes tuvieron evidencia de seroconversión en una sola muestra de sangre post-conversión. N=negativo, D1=DENV-1, D2=DENV-2, D3=DENV-3, D12=DENV-1 y -2, D13=DENV-1 y -3, D23=DENV-2 y -3, D123 = DENV-1, -2 y -3, D12*=DENV-1 o DENV-1 y -2, D12**=DENV-2 o DENV-1 y -2.

Perfil	FALSO+	VERDERO+	TOTAL	Factor de Corrección
N-D1	75	28	103	0.2718
N-D2	43	11	54	0.2037
N-D3	0	94	94	1.0000
N-D12	43	11	54	0.2037
N-D13	0	14	14	1.0000
N-D123	3	15	18	0.8333
D1-D12	104	59	163	0.3620
D1-D13	0	13	13	1.0000
D1-D123	3	49	52	0.9423
D2-D23	0	26	26	1.0000
D2-D12	98	54	152	0.3553
D2-D123	7	64	71	0.9014
D3-D13	14	0	14	0.0000
D3-D23	1	0	1	0.0000
D3-D123	7	0	7	0.0000
D12-D123	218	318	536	0.5933
D13-D123	5	1	6	0.1667
D23-D123	2	2	4	0.5000
D12*-D123	1	24	25	0.9600
D12**-D123	1	10	11	0.9091

*Los participantes positivos para DENV-1 o DENV-1/-2 en la primera muestra; **Los participantes positivos para DENV-2 o DENV-1/-2 en la primera muestra. En ambos casos los participantes no tuvieron un titulo de anticuerpos anti-DENV-3 sobre los valores de corte en las muestras posteriores a la seroconversión. Para los cálculos de incidencia éstos individuos fueron considerados monotípicos.

Tabla 2. El número de participantes inscritos en la cohorte entre1999-2005 por zona geográfica.

Zona	N° Enrolados	N° Manzanas	N° Promedio de personas por manzana (SE) / Rango	% de Enrolados con > 1 muestras de sangre	% Enrolados con anticuerpos anti-DENV durante la muestra basal en 1999	
					1 muestra	>1 muestra
BG	559	40	14.0 (2.6) / 1-70	80.1	82.0	65.6
IQ	406	36	11.3 (2.7) / 1-98	88.2	84.6	80.8
MC	485	53	9.2 (0.9) / 1-30	85.6	78.0	87.1
MY	639	60	10.7 (1.4) / 1-52	89.4	96.6	90.2
PT	479	48	10.0 (1.3) / 1-34	86.0	83.3	72.4
PU	617	50	12.3 (1.6) / 1-46	80.1	83.8	84.8
SA	499	51	9.8 (1.5) / 1-42	81.2	68.2	87.0
TA	902	54	16.7 (2.1) / 1-87	88.7	83.9	69.8

Tabla 3. Resumen de los resultados serológicos de 3,903 participantes (15,780 intervalos de monitoreo) proporcionando $\geq$ 2 muestras sanguíneas en el estudio de cohorte longitudinal, Iquitos, Perú 1999-2005. El estado serológico de cada sujeto del estudio (N°) se mantuvo constante para todos los intervalos de monitoreo a lo largo de su participación (No seroconversión) o mostraban evidencia serológica de infección con serotipos 1, 2 ó 3 de dengue (Seroconversiones). El panel superior muestra el número de participantes y los intervalos de monitoreo donde no se observaron cambios serológicos mostrando el porcentaje de intervalos donde anticuerpos a otros serotipos de DENV fueron observados (falso+/anticuerpos cruzados) para 1 ó 2 intervalos de monitoreo o donde los títulos cayeron bajo los valores de corte por un sólo intervalo. El panel inferior muestra el número de seroconversiones observadas.

Estatus				Intervalos				
						Falso (+)		Falso (-)
		N°	%	Total	Consistente[a]	1[b]	2[c]	1 intervalo[d]
No Seroconversión								
Negativo	N-N	585	23.0	2833	2435 (86.0%)	14.0%	1.4%	
Monotípico		620	24.4	3326				
	D1-D1	285	11.2	1429	1058 (74.0%)	20.6%	2.0%	5.3%
	D2-D2	295	11.6	1696	1290 (76.1%)	17.9%	1.2%	6.1%
	D3-D3	40	1.6	201	124 (61.7%)	34.3%	5.4%	4.0%
Politípico		1337	52.6	8066				
	D12-D12	1144	45.0	6393	5127 (80.2%)	8.0%	1.9%	11.8%
	D13-D13	8	0.3	61	16 (26.2%)	49.2%	8.1%	24.6%
	D23-D23	7	0.3	53	10 (20.4%)	77.6%	22.4%	10.2%
	D123-	97	3.8	936	739 (79.0%)	-	1.9%	21.0%
	D123	57	2.2	443	421 (95.0%)	2.0%	0.2%	2.9%
	D**1**2-D**1**2	24	0.9	184	166 (90.2%)	2.2%	-	7.6%
	D1**2**-D1**2**							
Total		2542	65.1	14229	11386 (80.0%)			

Seroconversiones				
	Una Sola	1281	32.8	1281
	Dobles	65	1.7	130
	Triples	1	0.03	3
Total		1347	34.5	1414

[a] Todos resultados de PRNT fueron iguales, por ejem., [N-N-N o N-N-N-N o D1-D1-D1-D1 o D12-D12-D12-D12-D12]; [b]por ejem. [N-N-D2-N-N-N o D1-D12-D1-D1-D1], [c]por ejem. [N-D2-D2-N-N-N o D2-D12-D12-D2-D2], [d]por ejem. [D2-D2-N-D2-D2 o D12-D12-D1-D12-D12]

Tabla 4. Resumen de los 1,414 seroconversiones detectadas en los participantes del estudio de cohorte 1999-2005 por medio del bioensayo PRNT en Iquitos, Perú.

Estatus			Seroconversiones			
			Inequívoca		Putativos[a]	
			N°	%	N°	%
1° Infecciones		N-D1	23	1.63	33	2.33
		N-D2	10	0.71	8	0.57
		N-D3	94[b]	6.65	60	4.24
		N-D12	11	0.78	24	1.70
		N-D13	14	0.99	23	1.63
		N-D23	2	0.14	3	0.21
		N-D123	15	1.06	17	1.20
		**TODA 1°**	**169**	**11.95**	**168**	**11.88**
2° Infecciones	DV-1	D2-D12	45	3.18	28	1.98
		D3-D13	0	0.00	7	0.50
		D23-D123	2	0.07	4	0.28
		**TODA D1**	**47**	**3.89**	**39**	**2.76**
	DV-2	D1-D12	54	3.82	45	3.18
		D3-D23	0	0.00	1	0.07
		D13-D123	1	0.07	0	0.00
		**TODA D2**	**55**	**3.89**	**46**	**3.25**
	DV-3	D1-D13	13	0.92	10	0.71
		D2-D23	26	1.84	8	0.57
		D12-D123	318[c]	22.49	209[d]	14.78
		D**1**2-D123	24	1.70	14	0.99
		D1**2**-D123	8	0.57	6	0.42
		**TODA D3**	**389**	**27.51**	**247**	**17.47**
	DV-1 o DV-2	D3-D123	0	0.00	6	0.42
	DV-1 o DV-3	D2-D123	63[e]	4.46	35	2.48
	DV-2 o DV-3	D1-D123	44[f]	3.11	44	3.11
		**TOTA 2°**	**598**	**42.29**	**417**	**29.49**
		TOTAL	767	54.24	585	41.37

[a]La seroconversión ocurrió en el último par de muestras de un participante (por ejem. [N-N-N-D1] o [D1-D1-D1-D12]). Para los cálculos de incidencia, la tasa cruda fue multiplicada por un factor de corrección basado en la proporción de perfiles de pares específicos (por ejem. N a D1, D1 a D12, D12 a D123) observados que se mantienen en muestras posteriores (por ejem., N-D1-D1 o D1-D12-D12) versus los que representan resultados falso+ (por ejem. N-D1-N o D1-D12-D1). [b]1 con 2nd muestra 12/8/2001. [c]14 participantes con fechas de la 2nd muestra en 2001 (Julio=3; Octubre=6; Noviembre=1; Diciembre=4). [d]9 participantes con 2nd muestra en 2001 (Agosto=1; Octubre=2; Diciembre =6). [e]2 participantes con 2nd muestra en 27/5/01 y 16/10/01. [f]1 participante con 2nd muestra 25/10/2001.

Tabla 5. Seroincidencia de infecciones primarias y secundarias para todos los serotipos de DENV entre Febrero de 1999 a Febrero de 2005. Las filas con letras en negrita incluyen sólo el componente escolar del estudio. Las tasas de seroincidencia fueron calculadas asumiendo que las infecciones ocurrieron en la fecha a la mitad del intervalo de muestreo. Las tasas de incidencia fueron ajustadas por los patrones de susceptibilidad de la población (ajustado por serotipo) y para la población completa (población-completa).

Fecha	Infecciones Primarias		Infecciones Secundarias	
	Ajustada por serotipo	Población completa	Ajustada por serotipo	Población completa
2/99-3/00	2.73 (2.52-2.93)	0.53 (0.51-0.55)	4.34 (4.17-4.51)	1.47 (1.42-1.53)
4/00-5/01	3.48 (3.25-3.71)	0.66 (0.64-0.68)	6.38 (6.17-6.59)	1.96 (1.90-2.02)
6/01-12/01	3.90 (3.47-4.33)	0.69 (0.66-0.72)	23.04 (21.86-24.22)	4.80 (4.58-5.02)
1/02-4/02	12.11 (10.43-13.78)	2.68 (2.51-2.85)	63.95 (59.30-68.59)	17.37 (16.27-18.48)
5/02-8/02	17.77 (15.64-19.89)	3.52 (3.35-3.70)	147.59 (139.00-156.18)	34.28 (32.55-36.00)
9/02-12/02	39.89 (35.45-44.34)	8.66 (8.21-9.10)	110.89 (103.62-118.16)	30.27 (28.72-31.82)
1/03-4/03	14.11 (12.21-16.00)	2.79 (2.62-2.95)	36.67 (33.69-39.65)	11.35 (10.67-12.03)
5/03-8/03	3.33 (0.07-6.60)	0.48 (0.34-0.63)	17.54 (10.52-24.56)	4.95 (3.48-6.41)
**9/03-5/04**	**5.68 (4.42-6.94)**	**0.92 (0.83-1.01)**	**20.87 (18.17-23.56)**	**5.84 (5.28-6.40)**
**8/04-2/05**	**23.54 (19.96-27.12)**	**7.79 (7.10-8.48)**	**28.34 (24.79-31.90)**	**14.18 (12.92-15.44)**

Tabla 6. Resumen de los casos de dengue identificados a través de la vigilancia de casos febriles en cohorte escolar entre Junio de 2000 y Diciembre de 2004.

		Bioensayo de Diagnostico					
Estatus		Detección Viral	SC de IgM	IgM Elevado[b]	SC IgG[c]	Síntomas Clínicos con/ PRNT[d]	Total
DENV-1	1°	2	2	-	-	3	7
	2°	1	-	1	-	2	4
DENV-2	1°	-	1	-	-	-	1
	2°	-	1	-	-	-	1
DENV-3	1°	9	2	9	-	2	22
	2°	22	10	7	3[a]	10	52
DENV-1/-3	1°	-	-	1	-	-	1
	2°	-	3	3	-	2	8
DENV-2/-3	1°	-	-	-	-	-	-
	2°	-	3	1	-	-	4
DENV-1/-2/-3	1°	-	2	-	-	1	3
	2°	-	-	-	-	-	-
Desconocido (DC)	1°	-	-	1	-	-	2
	2°	-	-	1	7	-	8
	DC	-	1	1	1	-	3
Total	1°	11	7	2	-	6	35
	2°	23	17	13	10	14	77
	DC	-	1	1	1	-	3

[a] Todos los 3 individuos fueron febriles a finales de Julio de 2001. [b]Incluye 11 participantes con títulos elevados de IgM de 1:100 con resultados de PRNT indicando la seroconversión durante el intervalo de muestreo, [c] 3 de 10 participantes con resultados de PRNT indicando seroconversión que fueron incluidos en los cálculos de incidencia. [d] Ninguno en ésta categoría se incluye para los cálculos de incidencia.

Tabla 7. Incidencia por serotipo especifico de DENV entre Febrero de 1999 a Febrero de 2005. Las filas con letra en negrita incluyen sólo el componente escolar del estudio. Las tasas de seroincidencia fueron calculados asumiendo que la fecha de la infección sucedió en el punto medio del intervalo de muestreo.

Fecha	Seroincidencia ajustada por serotipo (IC de 95%) y [La población completa(IC de 95%)] por 100 años-persona en riesgo							
	DV-1	DV-2	DV-3	DV-1/-2	DV-1/-3	DV-2/-3	DV-1/-2/-3	TOTAL
2/99-3/00	2.22 (2.09-2.35) [0.76 (0.74-0.79)]	3.46 (3.26-3.65) [1.15 (1.11-1.19)]	0.00 (0.00)	0.47 (0.44-0.51) [0.09 (0.09-0.09)]	0.00 (0.00)	0.00 [0.00]	0.00 [0.00]	6.15 (5.93-6.36) [2.00 (1.93-2.07)]
4/00-5/01	3.05 (2.90-3.21) [1.01 (0.98-1.04)]	3.46 (3.28-3.63) [1.13 (1.10-1.16)]	0.22 (0.21-0.22) [0.22 (0.21-0.22)]	1.01 (0.94-1.08) [0.19 (0.19-0.20)]	0.21 (0.20-0.22) [0.07 (0.07-0.07)]	0.00 [0.00]	0.00 [0.00]	7.95 (7.72-8.19) [2.62 (2.54-2.70)]
6/01-12/01	2.75 (2.53-2.97) [0.90 (0.86-0.94)]	1.26 (1.16-1.37) [0.39 (0.37-0.41)]	3.90 (3.72-4.09) [3.89 (3.71-4.07)]	0.21 (0.18-0.23) [0.04 (0.03-0.04)]	0.83 (0.76-0.90) [0.27 (0.26-0.28)]	0.29 (0.27-0.31) [0.09 (0.09-0.09)]	0.00 [0.00]	9.24 (8.81-9.67) [5.58 (5.32-5.84)]
1/02-4/02	7.15 (6.40-7.89) [2.85 (2.67-3.03)]	1.50 (1.34-1.66) [0.57 (0.53-0.61)]	13.13 (12.29-13.98) [12.94 (12.11-13.76)]	0.77 (0.66-0.87) [0.17 (0.16-0.18)]	6.29 (5.64-6.95) [2.51 (2.35-2.67)]	4.46 (3.99-4.93) [1.70 (1.59-1.80)]	1.53 (1.32-1.74) [0.34 (0.32-0.36)]	34.83 (32.59-37.07) [21.07 (19.73-22.42)]
5/02-8/02	1.16 (1.05-1.27) [0.39 (0.37-0.40)]	2.02 (1.84-2.20) [0.66 (0.62-0.69)]	33.82 (32.03-35.6) [30.64 (29.10-32.18)]	0.63 (0.55-0.70) [0.13 (0.12-0.14)]	13.05 (11.85-14.25) [4.23 (4.01-4.44)]	7.26 (6.59-7.92) [2.28 (2.17-2.40)]	1.95 (1.72-2.19) [0.39 (0.37-0.41)]	59.89 (56.75-63.02) [38.70 (36.76-40.65)]
9/02-12/02	3.06 (2.79-3.34) [1.00 (0.95-1.05)]	4.81 (4.41-5.22) [1.64 (1.56-1.73)]	37.72 (35.55-39.89) [29.64 (28.12-31.15)]	1.53 (1.37-1.70) [0.35 (0.34-0.37)]	17.64 (16.00-19.29) [5.41(5.14-5.69)]	15.10 (13.78-16.42) [4.84 (4.59-5.09)]	9.30 (8.26-10.34) [2.02 (1.91-2.12)]	89.18 (84.14-94.22) [44.91 (42.61-47.21)]
1/03-4/03	1.50 (1.35-1.65) [0.55 (0.52-0.59)]	0.93 (0.83-1.02) [0.33 (0.31-0.35)]	17.71 (16.40-19.02) [11.62 (10.93-12.32)]	1.57 (1.39-1.76) [0.40 (0.38-0.42)]	10.86 (9.67-12.05) [3.24 (3.05-3.43)]	7.80 (6.95-8.66) [2.21 (2.08-2.35)]	2.38 (1.97-2.58) [0.45 (0.42-0.48)	42.65 (39.69-45.61) [18.81 (17.69-19.94)]
5/03-8/03	0.00 (0.00)	0.00 (0.00)	8.00 (4.58-11.42) [5.18 (3.65-6.71)]	1.98 (0.75-3.21) [0.48 (0.34-0.63)]	10.88 (2.82-18.93) [2.14 (1.50-2.77)]	0.00 (0.00)	0.00 (0.00)	20.86 (13.13-28.58) [7.80 (5.49-10.10)]
**9/03-5/04**	**0.28 (0.24-0.32) [0.10 (0.09-0.11)]**	**0.37 (0.31-0.42) [0.13 (0.12-0.14)]**	**8.40 (7.37-9.43) [4.97 (4.49-5.44)]**	**0.16 (0.13-0.19) [0.04 (0.03-0.04)]**	**4.60 (3.78-5.42) [1.18 (1.06-1.29)]**	**5.49 (4.49-6.50) [1.38 (1.24-1.51)]**	**3.73 (2.90-4.55) [0.60 (0.55-0.66)]**	**23.02 (20.45-25.60) [8.39 (7.58-9.20)]**
**6/04-2/05**	**3.95 (3.49-4.42) [2.21 (2.02-2.41)]**	**0.84 (0.74-0.93) [0.47 (0.42-0.51)]**	**21.42 (19.12-23.73) [14.13 (12.87-15.38)]**	**0.92 (0.80-1.04) [0.41 (0.38-0.45)]**	**13.60 (11.76-15.44) [5.65 (5.14-6.15)]**	**7.90 (6.84-8.96) [3.32 (3.03-3.62)]**	**2.76 (2.34-3.18) [0.91 (0.83-0.99)]**	**51.39 (46.41-56.36) [27.10 (24.69-29.51)]**

Tabla 8. Incidencia por serotipo especifico de DENV en 1,100 participantes del cohorte de la vigilancia escolar entre Febrero de 1999 a Agosto de 2003.

Fecha	Seroincidencia ajustada por serotipo (IC de 95%) y [La población completa (IC de 95%)] por 100 años-persona en riesgo							
	DV-1	DV-2	DV-3	DV-1/-2	DV-1/-3	DV-2/-3	DV-1/-2/-3	TOTAL
2/99-3/00	1.71 (1.57-1.84)<br>[0.68 (0.65-0.72)]	2.80 (2.56-3.03)<br>[1.04 (0.99-1.10)]	0.00<br>[0.00]	0.11 (0.10-0.12)<br>[0.02 (0.02-0.03)]	0.00<br>[0.00]	0.00<br>[0.00]	0.00<br>[0.00]	4.61 (4.37-4.86)<br>[1.75 (1.66-1.84)]
4/00-5/01	2.76 (2.57-2.95)<br>[1.07 (1.02-1.12)]	2.85 (2.65-3.06)<br>[1.02 (0.98-1.07)]	0.16 (0.15-0.17)<br>[0.16 (0.15-0.17)]	0.37 (0.34-0.41)<br>[0.08 (0.08-0.08)]	0.00<br>[0.00]	0.00<br>[0.00]	0.00<br>[0.00]	6.15 (5.88-6.42)<br>[2.34 (2.23-2.44)]
6/01-12/01	3.23 (2.87-3.58)<br>[1.29 (1.20-1.37)]	1.31(1.16-1.45)<br>[0.46 (0.43-0.49)]	3.53 (3.29-3.78)<br>[3.53 (3.28-3.77)]	0.38 (0.33-0.44)<br>[0.08 (0.07-0.08)]	0.49 (0.43-0.54)<br>[0.19 (0.18-0.21)]	0.00<br>[0.00]	0.00<br>[0.00]	8.93 (8.32-9.55)<br>[5.55 (5.16-5.93)]
1/02-4/02	7.92 (6.82-9.01)<br>[3.84 (3.47-4.20)]	1.87 (1.60-2.14)<br>[0.78 (0.71-0.86)]	9.66 (8.74-10.59)<br>[9.56 (8.66-10.47)]	0.00<br>[0.00]	8.10 (6.97-9.22)<br>[3.92 (3.55-4.29)]	6.34 (5.40-7.27)<br>[2.66 (2.41-2.91)]	0.00<br>[0.00]	33.88 (30.64-37.13)<br>[20.77 (45.82-53.85)]
5/02-8/02	1.39 (1.21-1.57)<br>[0.58 (0.54-0.63)]	2.46 (2.14-2.79)<br>[0.91 (0.84-0.98)]	31.76 (29.17-34.35)<br>[29.10 (26.84-31.36)]	0.00<br>[0.00]	9.31 (8.11-10.51)<br>[3.82 (3.52-4.11)]	4.91 (4.25-5.57)<br>[1.74 (1.61-1.88)]	0.00<br>[0.00]	49.83 (4582-53.85)<br>[36.15 (33.35-38.96)]
9/02-12/02	2.85 (2.49-3.20)<br>[1.06 (0.98-1.114)]	4.97 (4.36-5.57)<br>[1.86 (1.72-2.00)]	34.65 (31.71-37.60)<br>[28.21 (26.07-30.35)]	0.46 (0.39-0.52)<br>[0.12 (0.11-0.13)]	14.67 (12.78-16.57)<br>[5.20 (4.81-5.60)]	12.01 (10.50-13.52)<br>[4.30 (3.97-4.63)]	7.97 (6.73-9.21)<br>[2.00 (1.85-2.15)]	77.57 (71.10-84.04)<br>[42.75 (39.51-45.99)]
1/03-4/03	2.09 (1.78-2.39)<br>[0.89 (0.81-0.98)	0.73 (0.62-0.84)<br>[0.29 (0.26-0.31)]	19.48 (17.24-21.72)<br>[13.33 (12.08-14.58)]	1.40 (1.15-1.65)<br>[0.40 (0.36-0.43)]	7.63 (6.39-8.88)<br>[2.65 (2.41-2.90)]	5.02 (4.18-5.87)<br>[1.52 (1.37-1.66)]	1.50 (1.19-1.80)<br>[0.33 (0.30-0.36)]	37.85 (33.79-41.92)<br>[19.40 (17.58-21.22)]
5/03-8/03	0.00<br>[0.00]	0.00<br>[0.00]	4.54 (2.38-6.70)<br>[3.33 (2.10-4.56)]	2.17 (0.43-3.91)<br>[0.57 (0.36-0.78)]	11.52 (1.42-21.61)<br>[2.53 (1.59-3.47)]	0.00<br>[0.00]	0.00<br>[0.00]	18.23 (10.43-26.03)<br>[6.43 (4.05-8.81)]

Figura 1. Enrolamiento y pérdida (LTF) de participantes, para el estudio del cohorte longitudinal (LC) y un subgrupo de estudio de Vigilancia de Febriles (FS) en escolares realizados entre Enero de 1999 y Febrero de 2005. Todos los participantes enrolados en LC proporcionaron muestras sanguíneas tomadas a intervalos de ~ 6-meses analizados por PRNT para NtAbs anti-DENV para identificar seroconversiones. Realizamos encuestas entomológicas hasta Agosto de 2003. La participación en FS incluía todas las actividades de LC más monitoreo de ausencias en los colegios desde inicios de Junio de 2000. Después de Agosto de 2003, sólo los participantes de FS continuaron siendo monitorizados serológicamente. El monitoreo de ausencias en los colegios continuó hasta Diciembre de 2004. Los participantes reclutados después de Agosto de 2003 proporcionaron las muestras para las pruebas serológicas de PRNT y todos los estudiantes que terminaron el estudio proporcionaron una muestra sanguínea final entre Diciembre de 2004 y Febrero de 2005. **Recuadro:** Muestra la distribución del número de muestras sanguíneas tomadas a intervalos de los participantes.

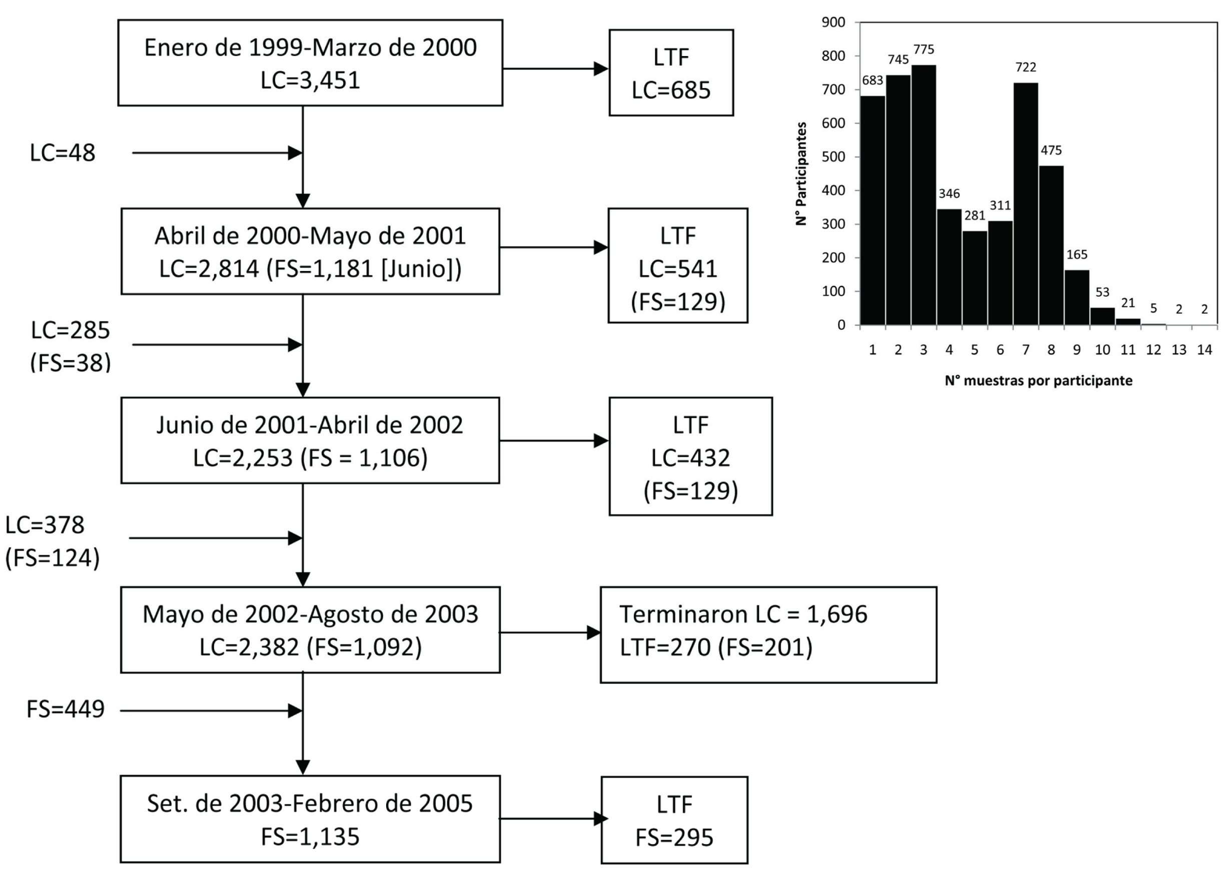

Figura 2. Número de participantes inscritos en la cohorte longitudinal de 1999-2005 por edades. El porcentaje de los enrolados que proporcionaron > 1 muestra de sangre fue consistente entre los grupos de edad (78-87%, P = 0,17).

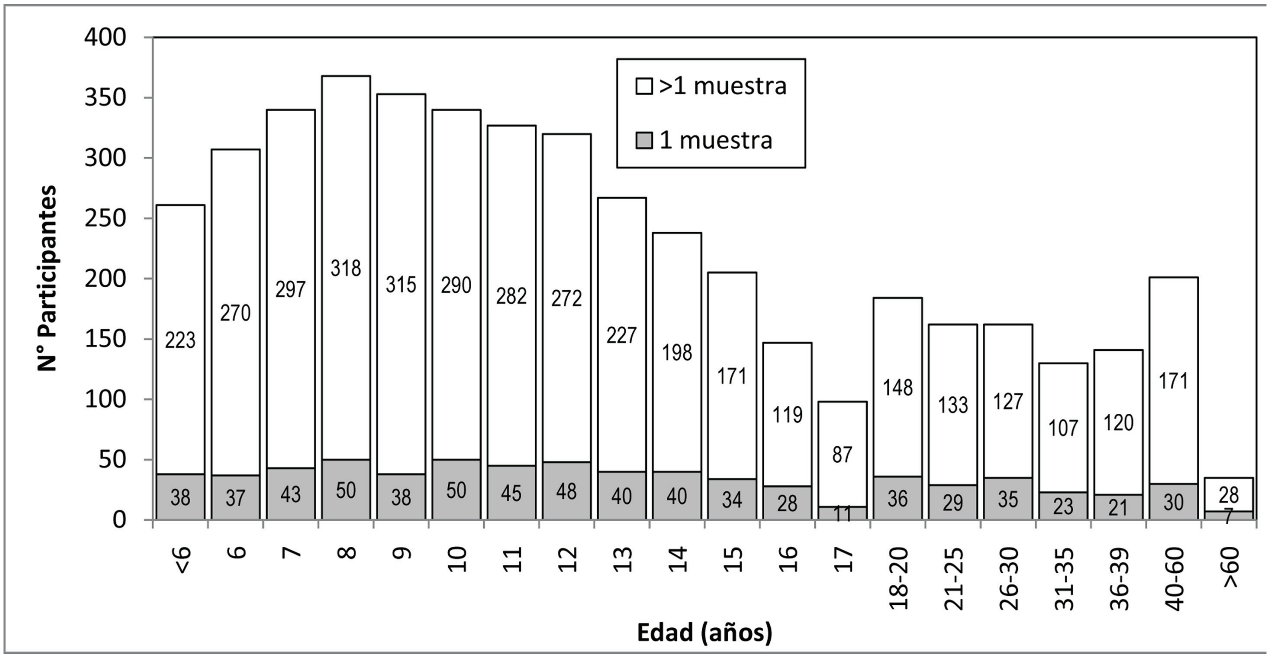

Figura 3. Tasas de seroprevalencia ajustadas por edad para los participantes enrolados en la cohorte longitudinal entre Febrero-Setiembre de 1999.

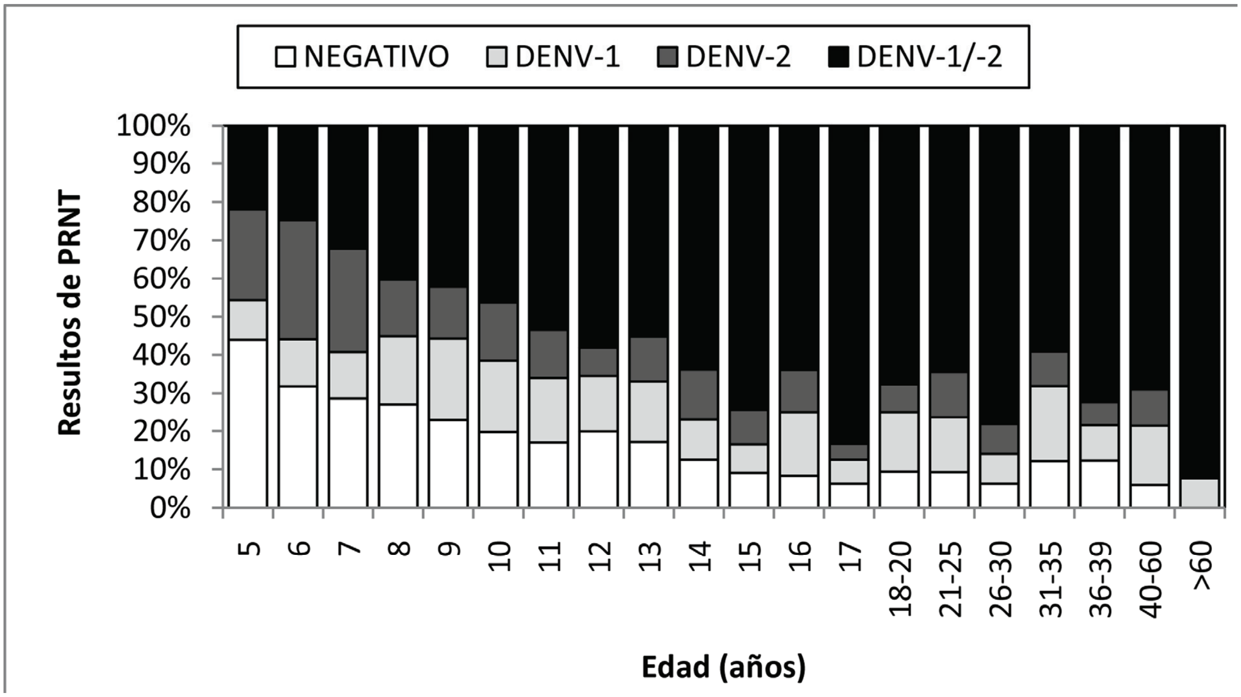

Figura 4. Tasas de seroprevalencia para los participantes enrolados en la cohorte entre Febrero – Setiembre de 1999 en las 8 zonas geográficas en Iquitos, Perú.

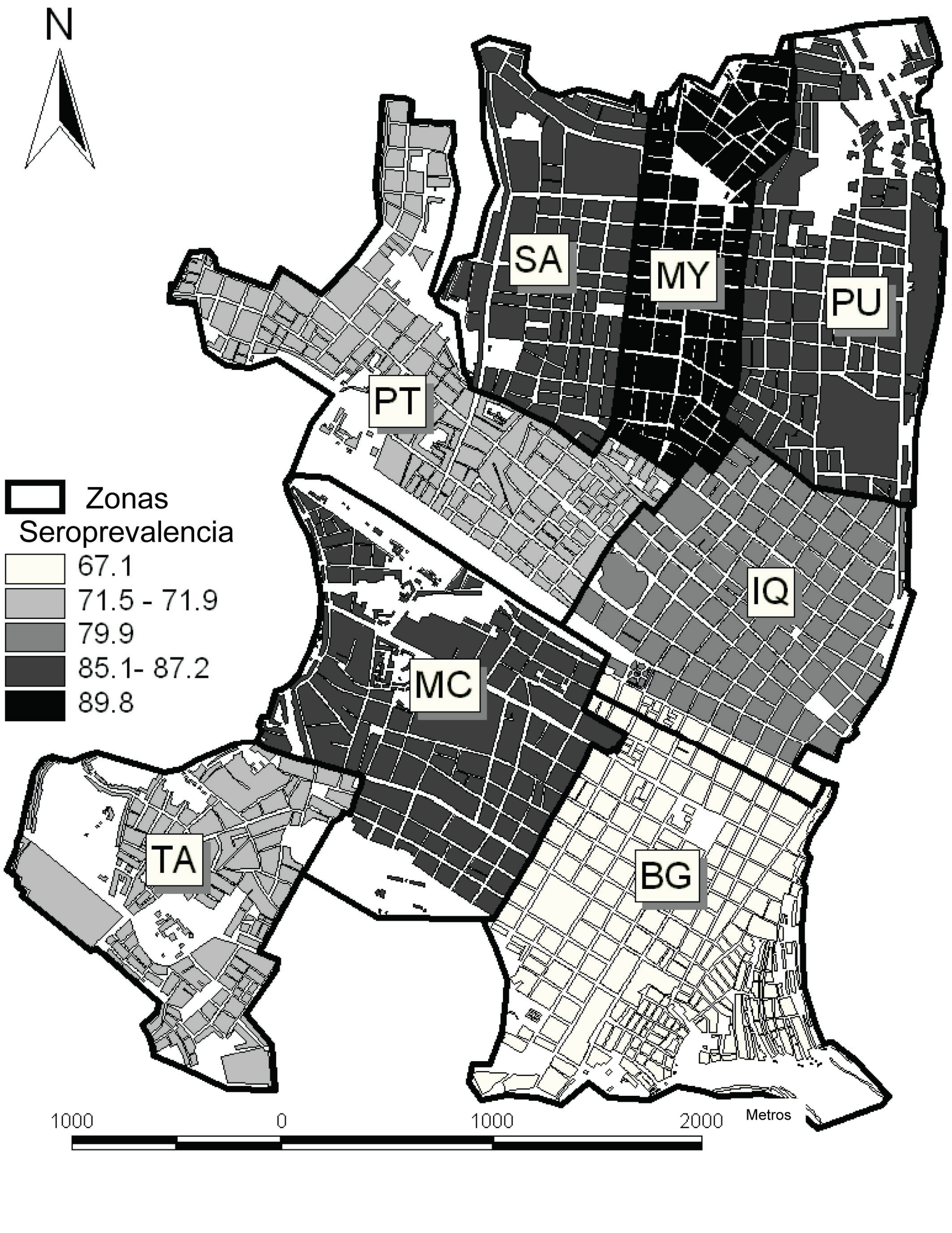

Figura 5. Comparación de las tasas de seroconversión e incidencias de casos clínicos de DENV entre Enero de1999 y Febrero de 2005. Las tasas de incidencia se expresan como casos o seroconversiones por 100 años-persona en riesgo. Las relaciones de casos sintomáticos a asintomáticos fueron calculados de la incidencia de casos de dengue activos entre el programa de vigilancia escolar (línea amarilla) comparada con las tasas de seroconversión global entre la cohorte completa susceptible (línea violeta) o el subgrupo en la cohorte escolar (línea rosada). Las relaciones que se muestran arriba, son las curvas por cada periodo de tiempo para la población susceptible (violeta) y la población completa (rosada). Las tasas de seroconversión para la cohorte longitudinal, asumiendo que la infección fue adquirida en la fecha del intervalo monitoreado (verde) o a la fecha final del intervalo monitoreado (azul) y para toda la población susceptible (oscura) y la población completa (claro).

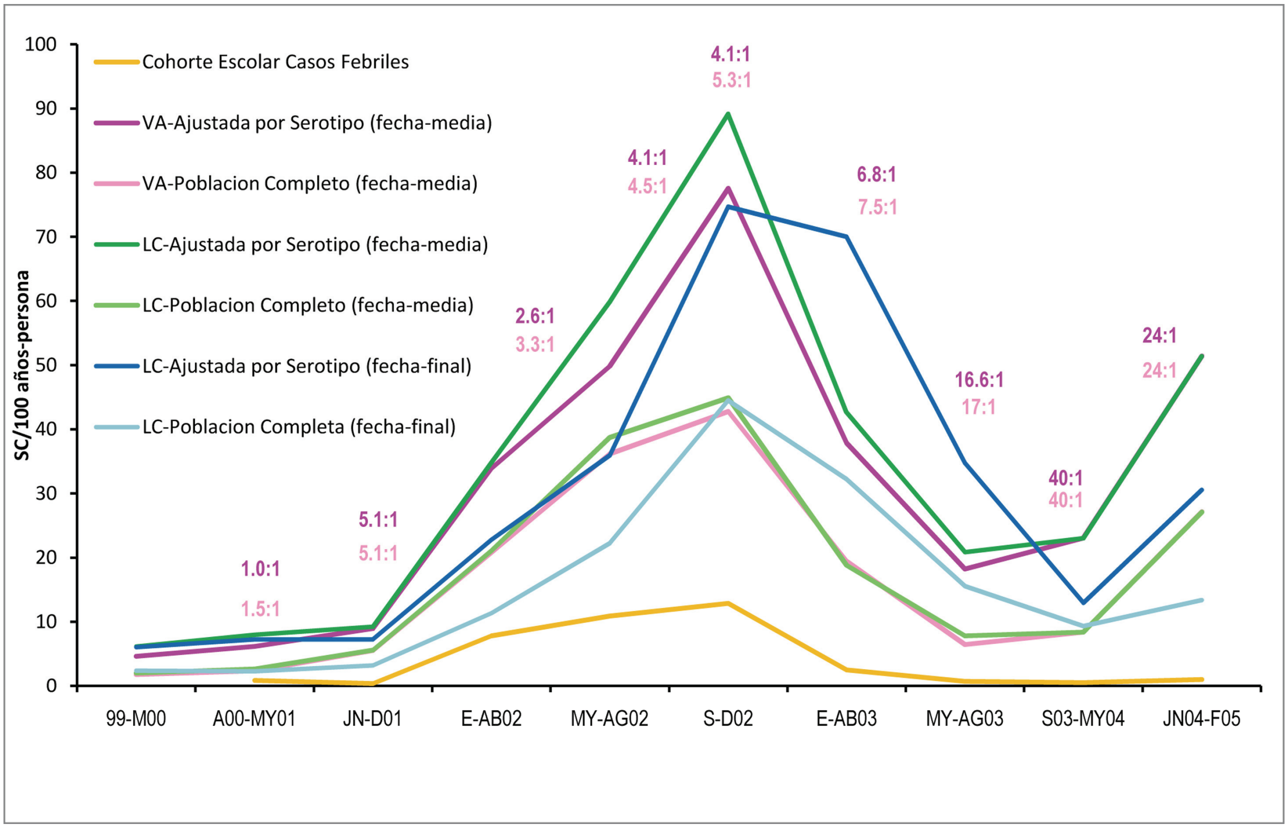

Figure 6. Las tasas de seroincidencia entre Enero 1999 a Febrero 2005 en 8 zonas geográficas en Iquitos, Perú. Las tasas están expresadas en 100 años-persona en riesgo con la tasa global para el periodo completo del estudio, mostrado en la parte superior izquierda de cada figura y las zonas en la parte superior derecha. Las tasas se calcularon para los mismos períodos de tiempo al igual que la Tabla 7.

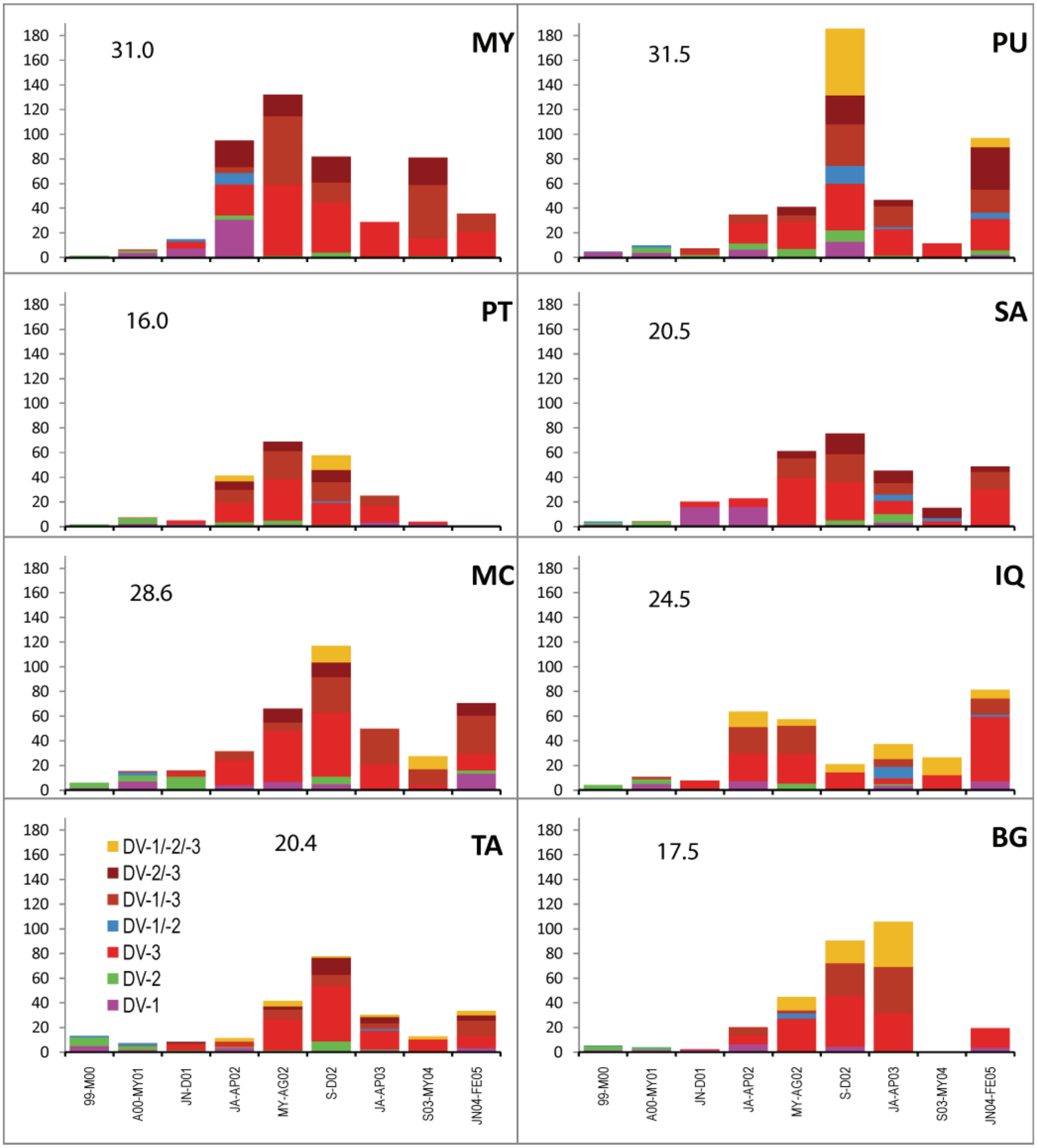

Tablas Supleméntales.

S1. Las fechas de enrolamiento y terminación de 4,586 participantes en el Estudio de Cohorte Longitudinal, realizado entre Enero de 1999 hasta Febrero de 2005. El Estudio consistió de un componente longitudinal que terminó en Agosto de 2003 y un componente de vigilancia active realizado entre Junio de 2000 y Diciembre de 2005.

	N° de Participantes por fecha de Enrolamiento y Retiro (Porcentaje)										
Fecha Enrol	Fecha de Retiro/Terminación										
	1/99-3/00	4/00-5/01	6/01-12/01	1/02-4/02	5/02-8/02	9/02-12/02	1/03-4/03	5/03-8/03	9/03-5/04	6/04-12/05	Total
1/99-3/00	685 (14.9)	523 (11.4)	302 (7.0)	87 (1.9)	75 (1.6)	139 (3.0)	662 (14.4)	539 (11.8)	64 (1.4)	375 (8.2)	3,451 (75.3)
4/00-5/01		18 (0.4)	3 (0.1)	2 (0.0)	1 (0.0)	2 (0.0)	9 (0.2)	12 (0.3)	0 (0.0)	1 (0.0)	48 (1.1)
6/01-12/01			0 (0.0)	0 (0.0)	0 (0.0)	0 (0.0)	0 (0.0)	0 (0.0)	0 (0.0)	0 (0.0)	0 (0.0)
1/02-4/02				38 (0.8)	4 (0.1)	17 (0.4)	170 (3.7)	19 (0.4)	4 (0.1)	33 (0.7)	285 (6.2)
5/02-8/02					19 (0.4)	4 (0.1)	42 (0.9)	112 (2.4)	3 (0.1)	47 (1.0)	227 (5.0)
9/02-12/02						9 (0.2)	61 (1.3)	33 (0.7)	0 (0.0)	7 (0.2)	110 (2.4)
1/03 4/03							0 (0.0)	37 (0.8)	1 (0.0)	3 (0.1)	41 (0.9)
5/03 8/03								0 (0.0)	0 (0.0)	0 (0.0)	0 (0.0)
9/03-5/04									90 (2.0)	235 (5.1)	327 (7.1)
6/04-2/05										99 (2.2)	99 (2.2)
Total	685 (14.9)	541 (11.8)	305 (6.7)	127 (2.8)	99 (2.2)	171 (3.7)	944 (20.6)	752 (16.4)	162 (3.5)	800 (17.4)	4586 (100.0)

S2. Resumen de perfiles serológicos donde una respuesta de anticuerpos cruzadas no-específicos fue observado por lo menos durante un intervalo de monitoreo antes que pudimos identificar el serotipo del virus infectante y seroconversiones "posibles" se excluyeron de los cálculos de incidencia. Además, la tabla muestra 62 seroconversions "posibles" que fueron excluidos de los cálculos de incidencia.

		Seroconversiones				
		Inequívoca			Posibles[b]	
Estatus		N°	Reacción X	%	N°.	%
1° Infecciones	N-D1	23	5	21.7	5	0.35
	N-D2	10	1	9.0	1	0.07
	N-D3	94[c]	18	19.1	0	0.00
	N-D12	11	1	9.1	0	0.00
	N-D13	14	3	21.4	0	0.00
	N-D23	2	1	50.0	0	0.00
	N-D123	15	-	-	0	0.00
	TOTAL	169	29	17.2	6	0.42
2° Infecciones						
DV-1	D2-D12	45	4	8.9	9	0.64
	D3-D13	0	-	-	0	0.00
	D23-D123	2	-	-	0	0.00
	TODA D1	47	4	8.5	9	0.35
DV-2	D1-D12	54	8	14.8	5	0.35
	D3-D23	0	-	-	0	0.00
	D13-D123	1	-	-	0	0.00
	TODA D2	55	8	14.5	5	0.35
DV-3	D1-D13	13	5		0	0.00
	D2-D23	26	16		0	0.00
	D12-D123	318[d]			34	2.40
	D**1**2-D123	24			0	0.00
	D1**2**-D123	8			2	0.14
	TODA D3	389			36	2.55
DV-1 or DV-2	D3-D123	0			0	0.00
DV-1 or DV-3	D2-D123	63[f]			1	0.07
DV-2 or DV-3	D1-D123	44[g]			5	0.35
	TOTAL	598			56	3.96
	TOTAL	767			62	4.38

[b]Seroconversiones donde los resultados de PRNT pre-conversión fueron circas los valores de corte con un solo pre-muestra (por ejem. [N-D2-D2]) o donde está indicada como un respuesta de DENV-3 cayendo.

S3. Incidencia por serotipo especifico de DENV entre Febrero de 1999 a Febrero de 2005 calculado asumiendo que la fecha de la infección sucedió en el punto medio del intervalo de muestreo. Los 62 seroconversiones clasificados como "posibles" fueron incluidos en los cálculos de las tasas. Las filas con letra en negrita incluyen sólo el componente escolar del estudio.

Fecha	Seroincidencia ajustada por serotipo y (La población completa) por 100 años-persona en riesgo							
	DV-1	DV-2	DV-3	DV-1/-2	DV-1/-3	DV-2/-3	DV-1/-2/-3	TOTAL
2/99-3/00	2.51 (0.86)	4.21 (1.40)	0.00 (0.00)	0.47 (0.09)	0.00 (0.00)	0.00 (0.00)	0.00 (0.00)	7.19 (2.35)
4/00-5/01	4.11 (1.36)	3.56 (1.16)	0.25 (0.25)	1.01 (0.19)	0.21 (0.07)	0.00 (0.00)	0.00 (0.00)	9.15 (3.04)
6/01-12/01	2.75 (0.90)	1.26 (0.39)	4.08 (4.07)	0.21 (0.04)	0.83 (0.27)	0.29 (0.09)	0.00 (0.00)	9.42 (5.75)
1/02-4/02	7.56 (3.01)	1.50 (0.57)	13.96 (13.76)	0.77 (0.17)	6.28 (2.50)	4.46 (1.69)	1.53 (0.34)	36.07 (22.05)
5/02-8/02	1.16 (0.38)	2.02 (0.65)	36.77 (33.35)	0.63 (0.13)	13.44 (4.32)	8.08 (2.52)	1.95 (0.38)	64.05 (41.73)
9/02-12/02	3.51 (1.15)	4.81 (1.64)	38.21 (30.03)	1.53 (0.35)	17.62 (5.41)	16.45 (5.27)	9.28 (2.01)	91.41 (45.86)
1/03-4/03	1.50 (0.55)	0.93 (0.33)	17.97 (11.80)	1.57 (0.40)	10.86 (3.24)	7.80 (2.21)	2.28 (0.45)	42.91 (18.98)
5/03-8/03	0.00 (0.00)	0.00 (0.00)	8.00 (5.18)	1.98 (0.48)	10.88 (2.14)	0.00 (0.00)	0.00 (0.00)	20.86 (7.80)
**9/03-5/04**	**0.28 (0.10)**	**0.37 (0.13)**	**8.40 (4.97)**	**0.16 (0.04)**	**4.60 (1.18)**	**5.49 (1.38)**	**3.73 (0.60)**	**23.02 (8.39)**
**8/04-2/05**	**3.95 (2.21)**	**0.84 (0.47)**	**21.94 (14.47)**	**0.92 (0.41)**	**13.60 (5.65)**	**7.90 (3.32)**	**2.76 (0.91)**	**51.91 (27.44)**

S4. Incidencia por serotipo especifico de DENV entre Febrero de 1999 a Febrero de 2005 calculado asumiendo que la fecha de la infección sucedió en la fecha final del intervalo de muestreo. Los 62 seroconversiones clasificados como "posibles" fueron excluidos de los cálculos de las tasas. Las filas con letra en negrita incluyen sólo el componente escolar del estudio.

Fecha	Seroincidencia ajustada por serotipo y (La población completa) por 100 años-persona en riesgo							
	DV-1	DV-2	DV-3	DV-1/-2	DV-1/-3	DV-2/-3	DV-1/-2/-3	TOTAL
2/99-3/00	2.92 (1.17)	2.92 (1.13)	0.00 (0.00)	0.19 (0.04)	0.00 (0.00)	0.00 (0.00)	0.00 (0.00)	6.02 (2.34)
4/00-5/01	2.27 (0.75)	3.99 (1.29)	0.00 (0.00)	0.87 (0.17)	0.10 (0.03)	0.00 (0.00)	0.00 (0.00)	7.23 (2.25)
6/01-12/01	2.82 (0.91)	2.04 (0.63)	1.43 (1.43)	0.75 (0.13)	0.21 (0.07)	0.00 (0.00)	0.00 (0.00)	7.24 (3.17)
1/02-4/02	9.45 (2.78)	0.71 (0.21)	6.80 (6.76)	1.20 (0.21)	3.06 (0.90)	1.57 (0.46)	0.00 (0.00)	22.79 (11.33)
5/02-8/02	3.25 (1.11)	2.16 (0.75)	16.01 (15.69)	0.00 (0.00)	8.23 (2.81)	4.32 (1.51)	1.97 (0.38)	35.94 (22.26)
9/02-12/02	1.34 (0.43)	2.89 (0.86)	38.06 (34.09)	1.27 (0.25)	14.64 (4.62)	11.87 (3.39)	4.61 (0.86)	74.68 (44.51)
1/03-4/03	2.27 (0.73)	3.59 (1.17)	26.20 (19.98)	1.09 (0.24)	15.90 (4.64)	13.17 (3.88)	7.79 (1.59)	70.02 (32.23)
5/03-8/03	0.86 (0.31)	0.75 (0.28)	25.00 (9.69)	1.69 (0.44)	8.71 (2.60)	5.95 (1.85)	1.76 (0.36)	34.70 (15.53)
**9/03-5/04**	**1.15 (0.60)**	**0.74 (0.32)**	**7.49 (6.80)**	**0.16 (0.05)**	**2.29 (1.11)**	**1.12 (0.43)**	**0.00 (0.00)**	**12.93 (9.26)**
**8/04-2/05**	**1.75 (0.73)**	**0.56 (0.24)**	**11.20 (7.31)**	**0.52 (0.16)**	**8.41 (2.61)**	**5.07 (1.64)**	**3.00 (0.68)**	**30.52 (13.37)**

S5. Incidencia por serotipo especifico de DENV entre Febrero de 1999 a Febrero de 2005 calculado asumiendo que la fecha de la infección sucedió en la fecha final del intervalo de muestreo. Los 62 seroconversiones clasificados como "posibles" fueron incluidos en los cálculos de las tasas. Las filas con letra en negrita incluyen sólo el componente escolar del estudio.

Fecha	Seroincidencia ajustada por serotipo y (La población completa) por 100 años-persona en riesgo							
	DV-1	DV-2	DV-3	DV-1/-2	DV-1/-3	DV-2/-3	DV-1/-2/-3	TOTAL
2/99-3/00	3.17 (1.27)	3.99 (1.55)	0.00 (0.00)	0.19 (0.04)	0.00 (0.00)	0.00 (0.00)	0.00 (0.00)	7.35 (2.86)
4/00-5/01	3.28 (1.10)	4.18 (1.36)	0.00 (0.00)	0.87 (0.17)	0.10 (0.03)	0.00 (0.00)	0.00 (0.00)	8.43 (2.65)
6/01-12/01	3.28 (0.97)	2.04 (0.63)	1.49 (1.49)	0.75 (0.13)	0.21 (0.07)	0.00 (0.00)	0.00 (0.00)	7.50 (3.30)
1/02-4/02	3.02 (2.78)	0.71 (0.21)	7.55 (7.51)	1.20 (0.21)	3.06 (0.90)	1.57 (0.46)	0.00 (0.00)	23.53 (12.06)
5/02-8/02	9.45 (1.29)	2.16 (0.75)	16.68 (16.36)	0.00 (0.00)	8.73 (2.98)	4.86 (1.69)	1.97 (0.38)	38.18 (23.45)
9/02-12/02	2.78 (0.43)	2.89 (0.85)	40.36 (36.21)	1.27 (0.24)	14.64 (4.56)	13.58 (3.83)	4.61 (0.85)	78.70 (46.97)
1/03-4/03	1.34 (0.87)	3.58 (1.16)	26.82 (20.48)	1.08 (0.24)	15.86 (4.62)	13.14 (3.86)	7.77 (1.58)	70.98 (32.82)
5/03-8/03	2.73 (0.31)	0.75 (0.28)	15.00 (9.69)	1.69 (0.44)	8.71 (2.60)	5.95 (1.85)	1.76 (0.36)	34.70 (15.53)
**9/03-5/04**	**1.15 (0.60)**	**0.74 (0.32)**	**7.49 (6.80)**	**0.16 (0.05)**	**2.29 (1.11)**	**1.12 (0.43)**	**0.00 (0.00)**	**12.93 (9.29)**
**8/04-2/05**	**1.75 (0.73)**	**0.56 (0.24)**	**11.38 (7.42)**	**0.52 (0.16)**	**8.41 (2.61)**	**5.07 (1.64)**	**3.00 (0.68)**	**30.70 (13.48)**

S6. Las fechas de enrolamiento y terminación de 4,586 participantes en el Estudio de Vigilancia de Febriles Escolar, realizado entre Junio de 2000 hasta Febrero de 2005.

	N° de Participantes por fecha de Enrolamiento y Retiro (Porcentaje)									
	Fecha de Retiro/Terminación									
Fecha Enrol	4/00-5/01	6/01-12/01	1/02-4/02	5/02-8/02	9/02-12/02	1/03-4/03	5/03-8/03	9/03-5/04	6/04-12/05	Total
4/00-5/01	129 (7.0)	35 (1.9)	89 (4.8)	20 (1.1)	20 (1.1)	67 (3.6)	71 (3.9)	254 (13.8)	500 (27.1)	1181 (64.0)
6/01-12/01		0 (0.0)	5 (0.3)	0 (0.0)	0 (0.0)	3 (0.2)	2 (0.1)	12 (0.7)	29 (1.6)	51 (2.8)
1/02-4/02			0 (0.0)	1 (0.1)	0 (0.0)	2 (0.1)	3 (0.2)	7 (0.4)	25 (1.4)	38 (2.1)
5/02-8/02				0 (0.0)	3 (0.2)	3 (0.2)	4 (0.2)	18 (1.0)	72 (3.9)	100 (5.4)
9/02-12/02					0 (0.0)	2 (0.1)	0 (0.0)	4 (0.2)	17 (0.9)	23 (1.3)
1/03 4/03						0 (0.0)	0 (0.0)	0 (0.0)	0 (0.1)	0 (0.1)
5/03 8/03							0 (0.0)	0 (0.0)	1 (0.1)	1 (0.1)
9/03-5/04								0 (0.0)	358 (19.4)	358 (19.4)
6/04-2/05									0 (0.0)	90 (4.9)
Total	129 (7.0)	35 (1.9)	94 (5.1)	21 (1.1)	23 (1.3)	77 (4.2)	80 (4.3)	295 (16.0)	1092 (59.2)	1846 (100.0)
