# Supplementary material for: Epidemiology of Dengue Virus in Iquitos, Peru 1999 to 2005: Interepidemic and Epidemic Patterns of Transmission
Source: PLoS Negl Trop Dis. 2010 May 4;4(5):e670. doi: 10.1371/journal.pntd.0000670 (PMC2864256; doi:10.1371/journal.pntd.0000670)
Supplement: Table S1 — Enrollment and termination dates for 4,586 participants in Longitudinal Cohort Study, carried out from January 1999 to February 2005. Study consisted of longitudinal component ending August 2003 and Active Surveillance component carried out from June 2000 through December 2005. (0.07 MB DOC) [file pntd.0000670.s003.doc]

|  | No. Participants by Enrollment and Withdrawal Date (Percent) | | | | | | | | | | |
| --- | --- | --- | --- | --- | --- | --- | --- | --- | --- | --- | --- |
| Enroll  Date | Withdrawal/Termination | | | | | | | | | | |
|  | 1/99-  3/00 | 4/00-  5/01 | 6/01-  12/01 | 1/02-  4/02 | 5/02-  8/02 | 9/02-  12/02 | 1/03-  4/03 | 5/03-  8/03 | 9/03-  5/04 | 6/04-  12/05 | Total |
| 1/99-  3/00 | 685 | 523 | 302 | 87 | 75 | 139 | 662 | 539 | 64 | 375 | 3,451 |
|  | (14.9) | (11.4) | (7.0) | (1.9) | (1.6) | (3.0) | (14.4) | (11.8) | (1.4) | (8.2) | (75.3) |
| 4/00-  5/01 |  | 18 | 3 | 2 | 1 | 2 | 9 | 12 | 0 | 1 | 48 |
|  |  | (0.4) | (0.1) | (0.0) | (0.0) | (0.0) | (0.2) | (0.3) | (0.0) | (0.0) | (1.1) |
| 6/01-  12/01 |  |  | 0 | 0 | 0 | 0 | 0 | 0 | 0 | 0 | 0 |
|  |  |  | (0.0) | (0.0) | (0.0) | (0.0) | (0.0) | (0.0) | (0.0) | (0.0) | (0.0) |
| 1/02-  4/02 |  |  |  | 38 | 4 | 17 | 170 | 19 | 4 | 33 | 285 |
|  |  |  |  | (0.8) | (0.1) | (0.4) | (3.7) | (0.4) | (0.1) | (0.7) | (6.2) |
| 5/02-  8/02 |  |  |  |  | 19 | 4 | 42 | 112 | 3 | 47 | 227 |
|  |  |  |  |  | (0.4) | (0.1) | (0.9) | (2.4) | (0.1) | (1.0) | (5.0) |
| 9/02-  12/02 |  |  |  |  |  | 9 | 61 | 33 | 0 | 7 | 110 |
|  |  |  |  |  |  | (0.2) | (1.3) | (0.7) | (0.0) | (0.2) | (2.4) |
| 1/03  4/03 |  |  |  |  |  |  | 0 | 37 | 1 | 3 | 41 |
|  |  |  |  |  |  |  | (0.0) | (0.8) | (0.0) | (0.1) | (0.9) |
| 5/03  8/03 |  |  |  |  |  |  |  | 0 | 0 | 0 | 0 |
|  |  |  |  |  |  |  |  | (0.0) | (0.0) | (0.0) | (0.0) |
| 9/03-  5/04 |  |  |  |  |  |  |  |  | 90 | 235 | 327 |
|  |  |  |  |  |  |  |  |  | (2.0) | (5.1) | (7.1) |
| 6/04-  2/05 |  |  |  |  |  |  |  |  |  | 99 | 99 |
|  |  |  |  |  |  |  |  |  |  | (2.2) | (2.2) |
| Total | 685 | 541 | 305 | 127 | 99 | 171 | 944 | 752 | 162 | 800 | 4586 |
|  | (14.9) | (11.8) | (6.7) | (2.8) | (2.2) | (3.7) | (20.6) | (16.4) | (3.5) | (17.4) | (100.0) |
