# Supplementary material for: Epidemiology of Dengue Virus in Iquitos, Peru 1999 to 2005: Interepidemic and Epidemic Patterns of Transmission
Source: PLoS Negl Trop Dis. 2010 May 4;4(5):e670. doi: 10.1371/journal.pntd.0000670 (PMC2864256; doi:10.1371/journal.pntd.0000670)
Supplement: Table S2 — Summary serological profiles where a non-specific broadly cross-reactive antibody response was observed for at least one monitoring interval before the infecting serotype could be identified and possible seroconversions excluded from incidence calculations. In addition, table shows 62 possible seroconversions that were excluded from our incidence calculations. (0.08 MB DOC) [file pntd.0000670.s004.doc]

|  |  | Seroconversions | | | | |
| --- | --- | --- | --- | --- | --- | --- |
|  |  | Unequivocal | | | Possible^b^ | |
| Status |  | No. | X-Reaction | % | No. | % |
| 1° Infections | N-D1 | 23 | 5 | 21.7 | 5 | 0.35 |
|  | N-D2 | 10 | 1 | 9.0 | 1 | 0.07 |
|  | N-D3 | 94^c^ | 18 | 19.1 | 0 | 0.00 |
|  | N-D12 | 11 | 1 | 9.1 | 0 | 0.00 |
|  | N-D13 | 14 | 3 | 21.4 | 0 | 0.00 |
|  | N-D23 | 2 | 1 | 50.0 | 0 | 0.00 |
|  | N-D123 | 15 | - | - | 0 | 0.00 |
|  | TOTAL | 169 | 29 | 17.2 | 6 | 0.42 |
|  |  |  |  |  |  |  |
| 2° Infections |  |  |  |  |  |  |
| DV-1 | D2-D12 | 45 | 4 | 8.9 | 9 | 0.64 |
|  | D3-D13 | 0 | - | - | 0 | 0.00 |
|  | D23-D123 | 2 | - | - | 0 | 0.00 |
|  | ALL D1 | 47 | 4 | 8.5 | 9 | 0.35 |
|  |  |  |  |  |  |  |
| DV-2 | D1-D12 | 54 | 8 | 14.8 | 5 | 0.35 |
|  | D3-D23 | 0 | - | - | 0 | 0.00 |
|  | D13-D123 | 1 | - | - | 0 | 0.00 |
|  | ALL D2 | 55 | 8 | 14.5 | 5 | 0.35 |
|  |  |  |  |  |  |  |
| DV-3 | D1-D13 | 13 | 5 |  | 0 | 0.00 |
|  | D2-D23 | 26 | 16 |  | 0 | 0.00 |
|  | D12-D123 | 318^d^ |  |  | 34 | 2.40 |
|  | D**1**2-D123 | 24 |  |  | 0 | 0.00 |
|  | D1**2**-D123 | 8 |  |  | 2 | 0.14 |
|  | ALL D3 | 389 |  |  | 36 | 2.55 |
|  |  |  |  |  |  |  |
| DV-1 or DV-2 | D3-D123 | 0 |  |  | 0 | 0.00 |
| DV-1 or DV-3 | D2-D123 | 63^f^ |  |  | 1 | 0.07 |
| DV-2 or DV-3 | D1-D123 | 44^g^ |  |  | 5 | 0.35 |
|  | TOTAL | 598 |  |  | 56 | 3.96 |
|  |  |  |  |  |  |  |
|  | TOTAL | 767 |  |  | 62 | 4.38 |

^b^Seroconversions where pre-conversions PRNT results were near cutoff values with a single pre-sample (eg. [N-D2-D2]) or where indicated as a falling D3 response.
