# Supplementary material for: Epidemiology of Dengue Virus in Iquitos, Peru 1999 to 2005: Interepidemic and Epidemic Patterns of Transmission
Source: PLoS Negl Trop Dis. 2010 May 4;4(5):e670. doi: 10.1371/journal.pntd.0000670 (PMC2864256; doi:10.1371/journal.pntd.0000670)
Supplement: Table S3 — Serotype- specific DENV incidence between February 1999–February 2005 calculated assuming that infections occurred at the midpoint of a sampling interval. The 62 seroconversions classified as probable were included in the rate calculations. Bold rows include school-based component only. (0.05 MB DOC) [file pntd.0000670.s005.doc]

| Date | Serotype-adjusted Seroincidence (Population-based) per 100 Person-years @ risk | | | | | | | |
| --- | --- | --- | --- | --- | --- | --- | --- | --- |
|  | DV-1 | DV-2 | DV-3 | DV-1/-2 | DV-1/-3 | DV-2/-3 | DV-1/-2/-3 | TOTAL |
| 2/99-3/00 | 2.51  (0.86) | 4.21  (1.40) | 0.00  (0.00) | 0.47  (0.09) | 0.00  (0.00) | 0.00  (0.00) | 0.00  (0.00) | 7.19  (2.35) |
| 4/00-5/01 | 4.11  (1.36) | 3.56  (1.16) | 0.25  (0.25) | 1.01  (0.19) | 0.21  (0.07) | 0.00  (0.00) | 0.00  (0.00) | 9.15  (3.04) |
| 6/01-12/01 | 2.75  (0.90) | 1.26  (0.39) | 4.08  (4.07) | 0.21  (0.04) | 0.83  (0.27) | 0.29  (0.09) | 0.00  (0.00) | 9.42  (5.75) |
| 1/02-4/02 | 7.56  (3.01) | 1.50  (0.57) | 13.96  (13.76) | 0.77  (0.17) | 6.28  (2.50) | 4.46  (1.69) | 1.53  (0.34) | 36.07  (22.05) |
| 5/02-8/02 | 1.16  (0.38) | 2.02  (0.65) | 36.77  (33.35) | 0.63  (0.13) | 13.44  (4.32) | 8.08  (2.52) | 1.95  (0.38) | 64.05  (41.73) |
| 9/02-12/02 | 3.51  (1.15) | 4.81  (1.64) | 38.21  (30.03) | 1.53  (0.35) | 17.62  (5.41) | 16.45  (5.27) | 9.28  (2.01) | 91.41  (45.86) |
| 1/03-4/03 | 1.50  (0.55) | 0.93  (0.33) | 17.97  (11.80) | 1.57  (0.40) | 10.86  (3.24) | 7.80  (2.21) | 2.28  (0.45) | 42.91  (18.98) |
| 5/03-8/03 | 0.00  (0.00) | 0.00  (0.00) | 8.00  (5.18) | 1.98  (0.48) | 10.88  (2.14) | 0.00  (0.00) | 0.00  (0.00) | 20.86  (7.80) |
| **9/03-5/04** | **0.28**  **(0.10)** | **0.37**  **(0.13)** | **8.40**  **(4.97)** | **0.16**  **(0.04)** | **4.60**  **(1.18)** | **5.49**  **(1.38)** | **3.73**  **(0.60)** | **23.02**  **(8.39)** |
| **8/04-2/05** | **3.95**  **(2.21)** | **0.84**  **(0.47)** | **21.94**  **(14.47)** | **0.92**  **(0.41)** | **13.60**  **(5.65)** | **7.90**  **(3.32)** | **2.76**  **(0.91)** | **51.91**  **(27.44)** |
