# Supplementary material for: Epidemiology of Dengue Virus in Iquitos, Peru 1999 to 2005: Interepidemic and Epidemic Patterns of Transmission
Source: PLoS Negl Trop Dis. 2010 May 4;4(5):e670. doi: 10.1371/journal.pntd.0000670 (PMC2864256; doi:10.1371/journal.pntd.0000670)
Supplement: Table S4 — Serotype-specific DENV incidence between February 1999 and February 2005 calculated under the assumptions that infection occurred on the final date of a sampling interval. The 62 seroconversions classified as probable were excluded from the rate calculation. Bold rows include school-based component only. (0.05 MB DOC) [file pntd.0000670.s006.doc]

| Date | Serotype-adjusted Seroincidence (Population-based) per 100 Person-years @ risk | | | | | | | |
| --- | --- | --- | --- | --- | --- | --- | --- | --- |
|  | DV-1 | DV-2 | DV-3 | DV-1/-2 | DV-1/-3 | DV-2/-3 | DV-1/-2/-3 | TOTAL |
| 2/99-3/00 | 2.92  (1.17) | 2.92  (1.13) | 0.00  (0.00) | 0.19  (0.04) | 0.00  (0.00) | 0.00  (0.00) | 0.00  (0.00) | 6.02  (2.34) |
| 4/00-5/01 | 2.27  (0.75) | 3.99  (1.29) | 0.00  (0.00) | 0.87  (0.17) | 0.10  (0.03) | 0.00  (0.00) | 0.00  (0.00) | 7.23  (2.25) |
| 6/01-12/01 | 2.82  (0.91) | 2.04  (0.63) | 1.43  (1.43) | 0.75  (0.13) | 0.21  (0.07) | 0.00  (0.00) | 0.00  (0.00) | 7.24  (3.17) |
| 1/02-4/02 | 9.45  (2.78) | 0.71  (0.21) | 6.80  (6.76) | 1.20  (0.21) | 3.06  (0.90) | 1.57  (0.46) | 0.00  (0.00) | 22.79  (11.33) |
| 5/02-8/02 | 3.25  (1.11) | 2.16  (0.75) | 16.01  (15.69) | 0.00  (0.00) | 8.23  (2.81) | 4.32  (1.51) | 1.97  (0.38) | 35.94  (22.26) |
| 9/02-12/02 | 1.34  (0.43) | 2.89  (0.86) | 38.06  (34.09) | 1.27  (0.25) | 14.64  (4.62) | 11.87  (3.39) | 4.61  (0.86) | 74.68  (44.51) |
| 1/03-4/03 | 2.27  (0.73) | 3.59  (1.17) | 26.20  (19.98) | 1.09  (0.24) | 15.90  (4.64) | 13.17  (3.88) | 7.79  (1.59) | 70.02  (32.23) |
| 5/03-8/03 | 0.86  (0.31) | 0.75  (0.28) | 25.00  (9.69) | 1.69  (0.44) | 8.71  (2.60) | 5.95  (1.85) | 1.76  (0.36) | 34.70  (15.53) |
| **9/03-5/04** | **1.15**  **(0.60)** | **0.74**  **(0.32)** | **7.49**  **(6.80)** | **0.16**  **(0.05)** | **2.29**  **(1.11)** | **1.12**  **(0.43)** | **0.00**  **(0.00)** | **12.93**  **(9.26)** |
| **8/04-2/05** | **1.75**  **(0.73)** | **0.56**  **(0.24)** | **11.20**  **(7.31)** | **0.52**  **(0.16)** | **8.41**  **(2.61)** | **5.07**  **(1.64)** | **3.00**  **(0.68)** | **30.52**  **(13.37)** |
