# Supplementary material for: Epidemiology of Dengue Virus in Iquitos, Peru 1999 to 2005: Interepidemic and Epidemic Patterns of Transmission
Source: PLoS Negl Trop Dis. 2010 May 4;4(5):e670. doi: 10.1371/journal.pntd.0000670 (PMC2864256; doi:10.1371/journal.pntd.0000670)
Supplement: Table S5 — Serotype-specific DENV incidence between February 1999 and February 2005 calculated under the assumptions that infection occurred on the final date of a sampling interval. The 62 seroconversions classified as probable were included in the rate calculation. Bold rows include school-based component only. (0.05 MB DOC) [file pntd.0000670.s007.doc]

| Date | Serotype-adjusted Seroincidence (Population-based) per 100 Person-years @ risk | | | | | | | |
| --- | --- | --- | --- | --- | --- | --- | --- | --- |
|  | DV-1 | DV-2 | DV-3 | DV-1/-2 | DV-1/-3 | DV-2/-3 | DV-1/-2/-3 | TOTAL |
| 2/99-3/00 | 3.17  (1.27) | 3.99  (1.55) | 0.00  (0.00) | 0.19  (0.04) | 0.00  (0.00) | 0.00  (0.00) | 0.00  (0.00) | 7.35  (2.86) |
| 4/00-5/01 | 3.28  (1.10) | 4.18  (1.36) | 0.00  (0.00) | 0.87  (0.17) | 0.10  (0.03) | 0.00  (0.00) | 0.00  (0.00) | 8.43  (2.65) |
| 6/01-12/01 | 3.28  (0.97) | 2.04  (0.63) | 1.49  (1.49) | 0.75  (0.13) | 0.21  (0.07) | 0.00  (0.00) | 0.00  (0.00) | 7.50  (3.30) |
| 1/02-4/02 | 3.02  (2.78) | 0.71  (0.21) | 7.55  (7.51) | 1.20  (0.21) | 3.06  (0.90) | 1.57  (0.46) | 0.00  (0.00) | 23.53  (12.06) |
| 5/02-8/02 | 9.45  (1.29) | 2.16  (0.75) | 16.68  (16.36) | 0.00  (0.00) | 8.73  (2.98) | 4.86  (1.69) | 1.97  (0.38) | 38.18  (23.45) |
| 9/02-12/02 | 2.78  (0.43) | 2.89  (0.85) | 40.36  (36.21) | 1.27  (0.24) | 14.64  (4.56) | 13.58  (3.83) | 4.61  (0.85) | 78.70  (46.97) |
| 1/03-4/03 | 1.34  (0.87) | 3.58  (1.16) | 26.82  (20.48) | 1.08  (0.24) | 15.86  (4.62) | 13.14  (3.86) | 7.77  (1.58) | 70.98  (32.82) |
| 5/03-8/03 | 2.73  (0.31) | 0.75  (0.28) | 15.00  (9.69) | 1.69  (0.44) | 8.71  (2.60) | 5.95  (1.85) | 1.76  (0.36) | 34.70  (15.53) |
| **9/03-5/04** | **1.15**  **(0.60)** | **0.74**  **(0.32)** | **7.49**  **(6.80)** | **0.16**  **(0.05)** | **2.29**  **(1.11)** | **1.12**  **(0.43)** | **0.00**  **(0.00)** | **12.93**  **(9.29)** |
| **8/04-2/05** | **1.75**  **(0.73)** | **0.56**  **(0.24)** | **11.38**  **(7.42)** | **0.52**  **(0.16)** | **8.41**  **(2.61)** | **5.07**  **(1.64)** | **3.00**  **(0.68)** | **30.70**  **(13.48)** |
