# Supplementary material for: Epidemiology of Dengue Virus in Iquitos, Peru 1999 to 2005: Interepidemic and Epidemic Patterns of Transmission
Source: PLoS Negl Trop Dis. 2010 May 4;4(5):e670. doi: 10.1371/journal.pntd.0000670 (PMC2864256; doi:10.1371/journal.pntd.0000670)
Supplement: Table S6 — Enrollment and termination dates for 1,846 participants in Active Surveillance School Cohort Sub-Study, carried out from June 2000 to February 2005. (0.07 MB DOC) [file pntd.0000670.s008.doc]

|  | No. Participants by Enrollment and Withdrawal Date (Percent) | | | | | | | | | |
| --- | --- | --- | --- | --- | --- | --- | --- | --- | --- | --- |
| Enroll  Date | Withdrawal/Termination | | | | | | | | | |
|  | 4/00-  5/01 | 6/01-  12/01 | 1/02-  4/02 | 5/02-  8/02 | 9/02-  12/02 | 1/03-  4/03 | 5/03-  8/03 | 9/03-  5/04 | 6/04-  12/05 | Total |
| 4/00-  5/01 | 129 | 35 | 89 | 20 | 20 | 67 | 71 | 254 | 500 | 1181 |
|  | (7.0) | (1.9) | (4.8) | (1.1) | (1.1) | (3.6) | (3.9) | (13.8) | (27.1) | (64.0) |
| 6/01-  12/01 |  | 0 | 5 | 0 | 0 | 3 | 2 | 12 | 29 | 51 |
|  |  | (0.0) | (0.3) | (0.0) | (0.0) | (0.2) | (0.1) | (0.7) | (1.6) | (2.8) |
| 1/02-  4/02 |  |  | 0 | 1 | 0 | 2 | 3 | 7 | 25 | 38 |
|  |  |  | (0.0) | (0.1) | (0.0) | (0.1) | (0.2) | (0.4) | (1.4) | (2.1) |
| 5/02-  8/02 |  |  |  | 0 | 3 | 3 | 4 | 18 | 72 | 100 |
|  |  |  |  | (0.0) | (0.2) | (0.2) | (0.2) | (1.0) | (3.9) | (5.4) |
| 9/02-  12/02 |  |  |  |  | 0 | 2 | 0 | 4 | 17 | 23 |
|  |  |  |  |  | (0.0) | (0.1) | (0.0) | (0.2) | (0.9) | (1.3) |
| 1/03  4/03 |  |  |  |  |  | 0 | 0 | 0 | 0 | 0 |
|  |  |  |  |  |  | (0.0) | (0.0) | (0.0) | (0.1) | (0.1) |
| 5/03  8/03 |  |  |  |  |  |  | 0 | 0 | 1 | 1 |
|  |  |  |  |  |  |  | (0.0) | (0.0) | (0.1) | (0.1) |
| 9/03-  5/04 |  |  |  |  |  |  |  | 0 | 358 | 358 |
|  |  |  |  |  |  |  |  | (0.0) | (19.4) | (19.4) |
| 6/04-  2/05 |  |  |  |  |  |  |  |  | 0 | 90 |
|  |  |  |  |  |  |  |  |  | (0.0) | (4.9) |
| T*ota*l | 129 | 35 | 94 | 21 | 23 | 77 | 80 | 295 | 1092 | 1846 |
|  | (7.0) | (1.9) | (5.1) | (1.1) | (1.3) | (4.2) | (4.3) | (16.0) | (59.2) | (100.0) |
